# Discovery of a transdermally deliverable pentapeptide for activating AdipoR1 to promote hair growth

**DOI:** 10.15252/emmm.202013790

**Published:** 2021-09-06

**Authors:** Jungyoon Ohn, Kyung Wook Been, Jin Yong Kim, Eun Ju Kim, Taeyong Park, Hye‐Jin Yoon, Jeong Seok Ji, Miki Okada‐Iwabu, Masato Iwabu, Toshimasa Yamauchi, Yeon Kyung Kim, Chaok Seok, Ohsang Kwon, Kyu Han Kim, Hyung Ho Lee, Jin Ho Chung

**Affiliations:** ^1^ Department of Translational Medicine Seoul National University College of Medicine Seoul Korea; ^2^ Department of Dermatology Seoul National University College of Medicine Seoul Korea; ^3^ Department of Dermatology Seoul National University Hospital Seoul Korea; ^4^ Institute of Human‐Environment Interface Biology Seoul National University Seoul Korea; ^5^ Department of Chemistry College of Natural Sciences Seoul National University Seoul Korea; ^6^ Department of Diabetes and Metabolic Diseases Graduate School of Medicine The University of Tokyo Tokyo Japan

**Keywords:** adiponectin, adiponectin receptor 1, adiponectin receptor 1 agonist, hair growth, peptide, Pharmacology & Drug Discovery, Skin

## Abstract

Alopecia induced by aging or side effects of medications affects millions of people worldwide and impairs the quality of life; however, there is a limit to the current medications. Here, we identify a small transdermally deliverable 5‐mer peptide (GLYYF; P5) that activates adiponectin receptor 1 (AdipoR1) and promotes hair growth. P5 sufficiently reproduces the biological effect of adiponectin protein via AMPK signaling pathway, increasing the expression of hair growth factors in the dermal papilla cells of human hair follicle. P5 accelerates hair growth *ex vivo* and induces anagen hair cycle in mice *in vivo*. Furthermore, we elucidate a key spot for the binding between AdipoR1 and adiponectin protein using docking simulation and mutagenesis studies. This study suggests that P5 could be used as a topical peptide drug for alleviating pathological conditions, which can be improved by adiponectin protein, such as alopecia.

## Graphical abstract

This study presents an adiponectin‐derived, transdermally deliverable pentapeptide (GLYYF) that promotes hair growth by activating AdipoR1.

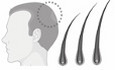

**The paper explained**

**Problem**

Hair loss is a distressing disorder affecting millions of people worldwide. It is caused by various reasons including aging, hormonal dysfunction, and side effect of several medications. Given the limited usage of existing hair growth‐promoting drugs due to concerns of side effects or partial efficacy, new drugs with novel mechanisms of action to treat alopecia are highly demanded.

**Results**

We designed a small peptide (P5; GLYYF) from the original sequence of endogenous adiponectin protein, which is effective in activating adiponectin receptor 1 (AdipoR1) *in vivo* and *in vitro*. P5 sufficiently reproduces the biological effect of adiponectin protein via AMPK signaling pathway, increasing the expression of hair growth factors in the dermal papilla cells of human hair follicle. P5 accelerates hair growth *ex vivo* and induces anagen hair cycle in mice *in vivo*. Furthermore, we elucidate a key spot for the binding between AdipoR1 and adiponectin protein using docking simulation and mutagenesis studies.

**Impact**

This study demonstrates that P5 is a potential peptide agent to promote hair growth by activating AdipoR1. Moreover, our findings of the crucial region in AdipoR1 for binding to P5 pave the way for the development of novel AdipoR1 modulating molecules in the future.

## Introduction

Hair loss is an obvious distressing disorder affecting millions of people worldwide (Adil & Godwin, 2017). It is caused by various reasons including aging, hormonal dysfunction, and side effect of several medications (Price, 1999). Each mammalian hair follicle (HF) consists of cyclic phases of telogen (quiescence), anagen (regeneration), and catagen (degeneration) (Müller‐Röver *et al*, 2001); thus, molecules that regulate the hair cycle to prolong or induce anagen phase by activating HF cells can be utilized as therapeutic agents (Ohn *et al*, 2019).

To date, two representative FDA‐approved drugs, finasteride and minoxidil, are used to treat patients with patterned alopecia by regulating the HF cycle with promoting hair growth. Finasteride inhibits type II 5α‐reductase, suppressing the conversion of testosterone to dihydrotestosterone in HFs (Rittmaster, 1994). Minoxidil is used in topical form, although its exact underlying mode of action in treating alopecia needs to be elucidated (Messenger & Rundegren, 2004). Given their limited usage due to concerns of side effects or partial efficacy, new drugs with novel mechanisms of action to treat alopecia are highly demanded (McClellan & Markham, 1999; Lucky *et al*, 2004; Gupta & Charrette, 2015).

Our previous studies found that adiponectin protein (APN) promotes hair growth *ex vivo* effectively, along with inducing mRNA expressions of key hair growth factors, including insulin‐like growth factor 1 (IGF‐1), vascular endothelial growth factor (VEGF), and hepatocyte growth factor (HGF), which suggests that APN is a promising molecule to treat alopecia (Won *et al*, 2012). However, it is not well understood about the underlying action mechanism of APN on hair growth promotion and how to utilize this endogenous protein as clinically applicable drug forms (Otvos, 2019).

In this study, we designed a small peptide (P5; GLYYF) from the original sequence of endogenous APN to allow transdermal delivery and discovered its role as an effective ligand for activating adiponectin receptor 1 (AdipoR1) *in vivo* and *in vitro*, based on the intensive end‐to‐end approach starting from *in silico* prediction, *in vitro* biochemical studies, and *ex vivo* HF organ culture and *in vivo* genetic mouse model studies, followed by molecular docking simulation. In doing this, we elucidated a key amino acid sequence crucial for the binding between AdipoR1 and APN, which could be applied in a strategy to discover new candidate drug by designing AdipoR1 activating peptides (Bos & Meinardi, 2000).

## Results

### Two potential small 5‐mer peptides are positioned within a highly conserved sequence of APN

**Figure 1:**
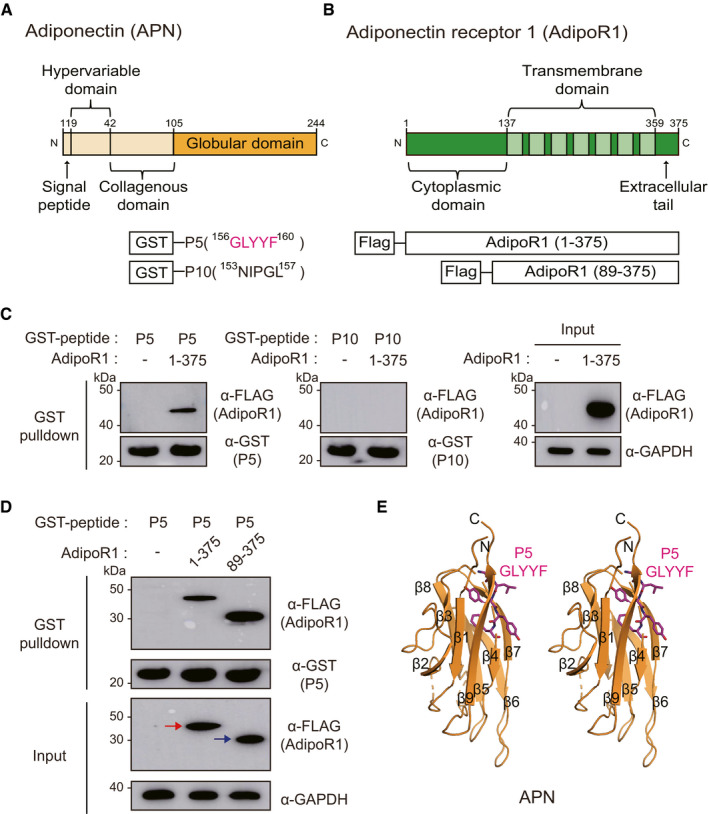
Direct molecular interaction between the P5 and AdipoR1 Domain architectures of APN and the GST‐fused 5‐mer peptides (GLYYF; GST‐P5, or NIPGL; GST‐P10).Domain architectures of AdipoR1 and Flag‐fused full‐length AdipoR1 (residue 1–375) or N‐terminal‐truncated AdipoR1 (residues 89–375). Each construct in the schematic diagram was used for the pulldown experiment.GST pulldown of transiently expressed AdipoR1 (residues 1–375) with immobilized GST‐P5 or GST‐P10 fusion proteins. Pulldown fractions were subjected to Western blotting using Flag and GST antibodies.GST pulldown of AdipoR1 (residues 1–375) or AdipoR1 (residues 89–375) with GST‐P5. The red or blue arrow indicates correct sized bands of AdipoR1 (residues 1–375) or AdipoR1 (residues 89–375), respectively.The position of P5 in APN. A stereo ribbon diagram of APN (PDB ID: 4DOU) is drawn, and P5 is shown in magenta sticks. Source data are available online for this figure.

**Figure EV1:**
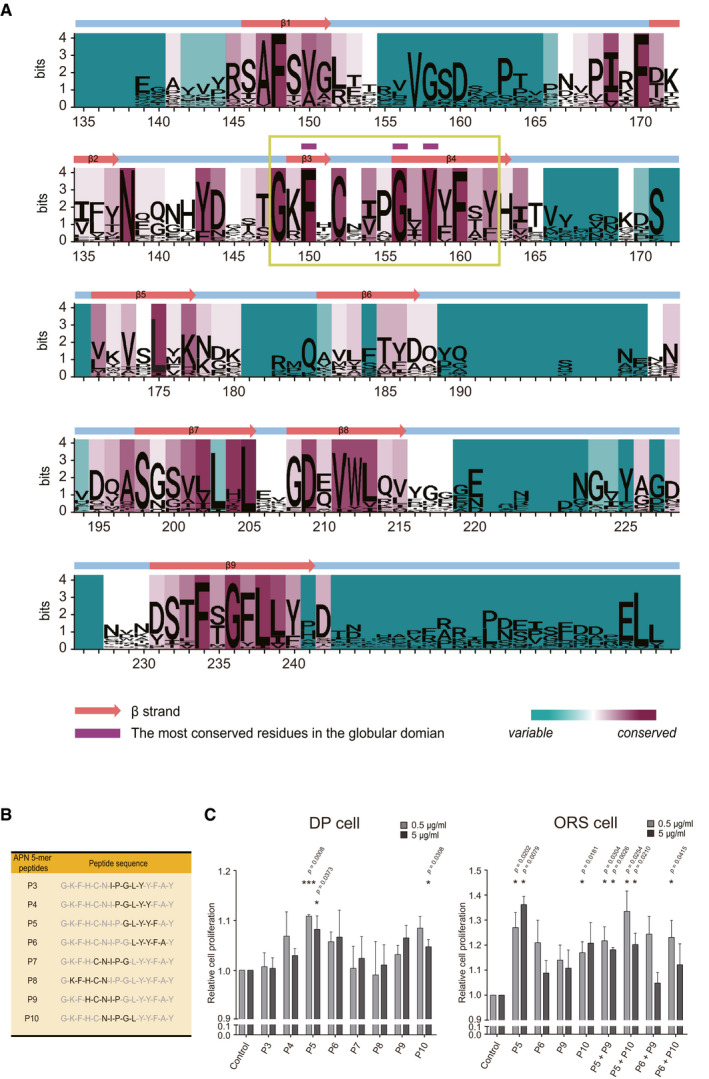
Excavation of the most conserved residues in APN globular domain, and the screening of candidate 5‐mer peptides based on DP and ORS cell proliferation Sequence logo landscape of MSA of APN globular domain. The residue numbers are based on the human APN amino acid sequence. In a given residue, the height of each letter (measured in bits) is proportionally scaled to the amount of information contributed based on Shannon entropy. The background color of each residue is determined by the degree of conservation of physicochemical properties (cyan to magenta). The most conserved residues (marked with purple tags) are located within a highly conserved sequence (designated in a green box).Candidate 5‐mer peptide sequences derived from a highly conserved sequence of APN globular domain. Each sequence is indicated in black.DP and ORS cells are used to screen the candidate 5‐mer peptides in terms of the cell proliferation effect (*n* = 3–5 biological replicates/group). Data are presented as mean ± SEM. Statistical significance was determined using Paired *t*‐test (*P < 0.05 or ***P < 0.001 compared to the control group). Source data are available online for this figure.

Human APN consists of four structural domains: a N‐terminal signal peptide, a hypervariable domain, a collagenous domain, and a C‐terminal globular domain, with the globular domain being biologically active (Fig 1A) (Scherer *et al*, 1995; Shapiro & Scherer, 1998; Takahashi *et al*, 2000; Fruebis *et al*, 2001; Tomas *et al*, 2002; Wong *et al*, 2004; Min *et al*, 2012). As an evolutionally conserved protein, APN shows a high degree of sequence homology in species with similar biological effects (Hotta *et al*, 2001; Berg *et al*, 2002; Straub & Scherer, 2019). We identified the most conserved amino acid sequence of globular domain of APN using sequences from the UniProtKB database (UniProt_Consortium, 2019). We observed several highly conserved residues in APN, including Phe150, Gly156, and Tyr158, based on the degree of conservation of the physicochemical properties (Fig EV1A). Around the three most conserved amino acid residues (Phe150, Gly156, and Tyr158), a relatively highly conserved peptide sequence (^148^GKFHCNIPGLYYFAY^162^) was identified. This 15‐mer sequence partially overlapped with or encompassed the sequence of AdipoRs agonists in previous studies (Otvos *et al*, 2011, 2014; Ma *et al*, 2017; Kim *et al*, 2018; Otvos, 2019).

To design transdermally deliverable AdipoR1 agonist, we further assessed the 15‐mer sequence by cutting it into several 5‐mer peptides (Fig EV1B) (Bos & Meinardi, 2000). Based on the fact that AdipoRs are expressed in HF cells, including dermal papilla (DP) and outer root sheath (ORS) cells (Won *et al*, 2012), we performed a cell proliferation assay on human DP and ORS cells to see if there is any potential biological effect of the 5‐mer peptides on the human HF cells. Among the 5‐mer peptides, ^156^GLYYF^160^ (P5) and ^153^NIPGL^157^ (P10) showed significant cell proliferative effects, providing a rationale to be assessed further (Fig EV1C).

### P5 directly binds with AdipoR1

**Figure EV2:**
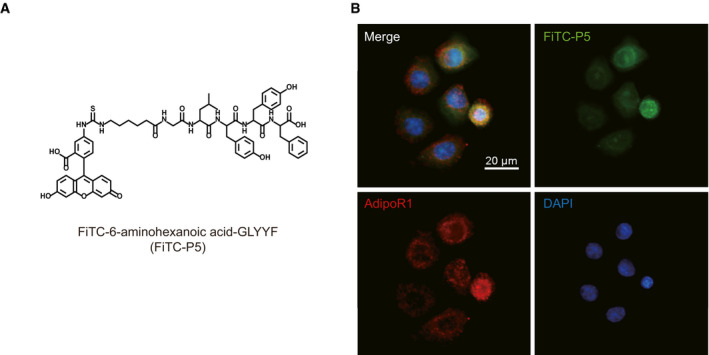
The colocalization of P5 and AdipoR1 *in vitro* Molecular structure of the FiTC‐conjugated P5 (FiTC‐P5).IF staining for AdipoR1 and FiTC‐P5 in cultured FiTC‐P5‐treated ORS cells.

To investigate whether two potential 5‐mer peptides (P5 and P10; Fig 1A) directly bind to AdipoR1, we performed protein‐protein interaction assays using glutathione S‐transferase (GST) pulldown method. After the purified GST‐fused P5 or P10 peptides were immobilized to GST beads, they were mixed with human embryonic kidney (HEK) 293T cell lysate expressing full‐length AdipoR1 (residues 1–375 in Fig 1B), and the pulldown fractions were analyzed by Western blots using Flag and GST antibodies, respectively. Surprisingly, we found that only P5 bound to AdipoR1 whereas P10 did not (Fig 1C). Similar to G protein‐coupled receptors, AdipoRs consist of seven transmembrane helices; however, they oriented oppositely to G protein‐coupled receptors in that the N‐terminal is located in the cytoplasm, while the C‐terminal is located in the extracellular space (Tanabe *et al*, 2015; Vasiliauskaité‐Brooks *et al*, 2017). Therefore, we also checked whether P5 could bind to an N‐terminal‐truncated AdipoR1 (residues 89–375 in Fig 1B), assuming that the N‐terminal part of AdipoR1 does not contribute to the AdipoR1‐P5 complex formation. As expected, both full‐length and N‐terminal‐truncated AdipoR1 (residues 1–375 and 89–375, respectively) bound to P5 (Fig 1D). For further analysis of the molecular interaction between AdipoR1 and P5 in viable cells, P5 conjugated with fluorescein isothiocyanate (FiTC‐P5, Fig EV2A) was treated on human ORS cells *in vitro*. Indeed, FiTC‐P5 was colocalized with AdipoR1 (Fig EV2B), suggesting that the molecular interaction between AdipoR1 and P5 is also present in viable cells *in vitro*.

When we examined the position of P5 in APN structure (PDB ID: 4DOU) (Min *et al*, 2012), P5 is a part of the β sheets in the jelly roll fold, which are arranged in two 5‐strand layers (Fig 1E). We hypothesized that the side chains of P5, which potentially bind to AdipoR1, will be surface‐exposed in APN structure if P5 moiety is hotspot of APN for binding to AdipoR1. Indeed, the second and fourth residues of P5 (Leu and Tyr, respectively) are surface‐exposed. Meanwhile, the third and fifth residues (Tyr and Phe, respectively) form a core interaction within APN, suggesting that they are crucial to maintain the correct folding of APN (Fig 1E).

### P5 activates AMPK signaling pathway through AdipoR1 in ORS cells and DP cells

**Figure 2:**
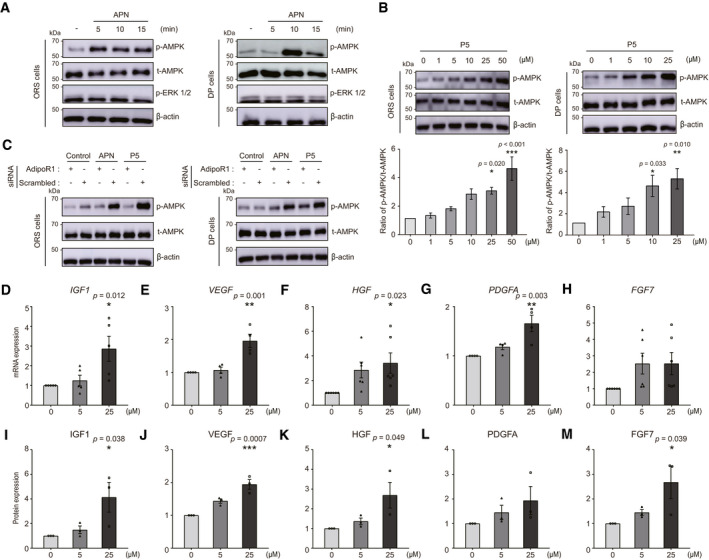
Both APN and P5 activate AMPK signaling pathway through AdipoR1 *in vitro*, and P5 induces hair growth factors in DP cells AAPN‐treated ORS or DP cell lysates were analyzed for p‐AMPK and p‐ERK1/2.BP5‐treated ORS or DP cells lysates were analyzed for p‐AMPK and t‐AMPK. Densitometric analysis for the ratio of p‐AMPK protein to t‐AMPK protein (*n* = 5 (ORS) and 4 (DP) biological replicates/group).CAdipoR1 siRNA‐transfected ORS or DP cells were treated with APN or P5. The cell lysates were analyzed for p‐AMPK.D–HThe relative gene expression levels of growth factors in P5‐treated DP cells (*n* = 4 (VEGF, PDGFA), 5 (IGF‐1), 6 (HGF, FGF7) biological replicates/group).I–MThe relative protein levels of growth factors in P5‐treated DP cells (*n* = 3 biological replicates/group). Data information: In (B and D–M), One‐way ANOVA with Tukey's test compared to each control group. Data are presented as the mean ± SEM. Source data are available online for this figure.

Following the confirmation of the molecular interaction between AdipoR1 and P5, we evaluated the activation of the intracellular signaling pathway by P5. We investigated whether APN treatment on ORS and DP cells could activate AMPK or ERK signaling pathways, which are previously known to be downstream effectors of AdipoR1 (Mao *et al*, 2006; Deepa & Dong, 2009; Shibata *et al*, 2012). The phosphorylation of AMPK was observed after APN treatment on ORS and DP cells while APN had no effect on the phosphorylation of ERK (Fig 2A). Using P5 for treatment on ORS and DP cells, we also found that the concentration‐dependent phosphorylation of AMPK (Fig 2B). Next, for determination of whether both P5 and APN mediates the phosphorylation of AMPK through AdipoR1 in ORS and DP cells, the cells were transfected with AdipoR1 siRNA (Appendix Fig S1). Based on Western blot analysis after the AdipoR1 siRNA transfection, the p‐AMPK intensity was reduced in both transfected ORS and DP cells after P5 or APN treatment (Fig 2C). Collectively, P5 activates AMPK signaling pathway through AdipoR1 in ORS and DP cells *in vitro*.

### P5 induces hair growth factor production in DP cells

Next, we investigated the downstream effects of P5 on hair growth factor genes and proteins in DP cells. We previously showed that APN induces mRNA expressions of key hair growth factor (Won *et al*, 2012). We tested whether P5 can also increase the mRNA levels and proteins of hair growth factors in DP cells: *IGF‐1*, *VEGF*, *HGF*, platelet‐derived growth factor alpha (*PDGFA*), and fibroblast growth factor (*FGF*) 7, which are known to induce or maintain the anagen phase of the hair cycle (Jindo *et al*, 1994; Philpott *et al*, 1994; Yano *et al*, 2001; Jang, 2005; Tomita *et al*, 2006; Iino *et al*, 2007). We found that mRNA expressions of the genes were significantly increased (Fig 2D–H), and their protein expression levels were elevated (Fig 2I–M) in the P5‐treated DP cells. These results suggest that P5 treatment reproduces the biological effects of APN *in vitro*.

### P5 promotes hair growth comparable to APN *ex vivo*

**Figure 3:**
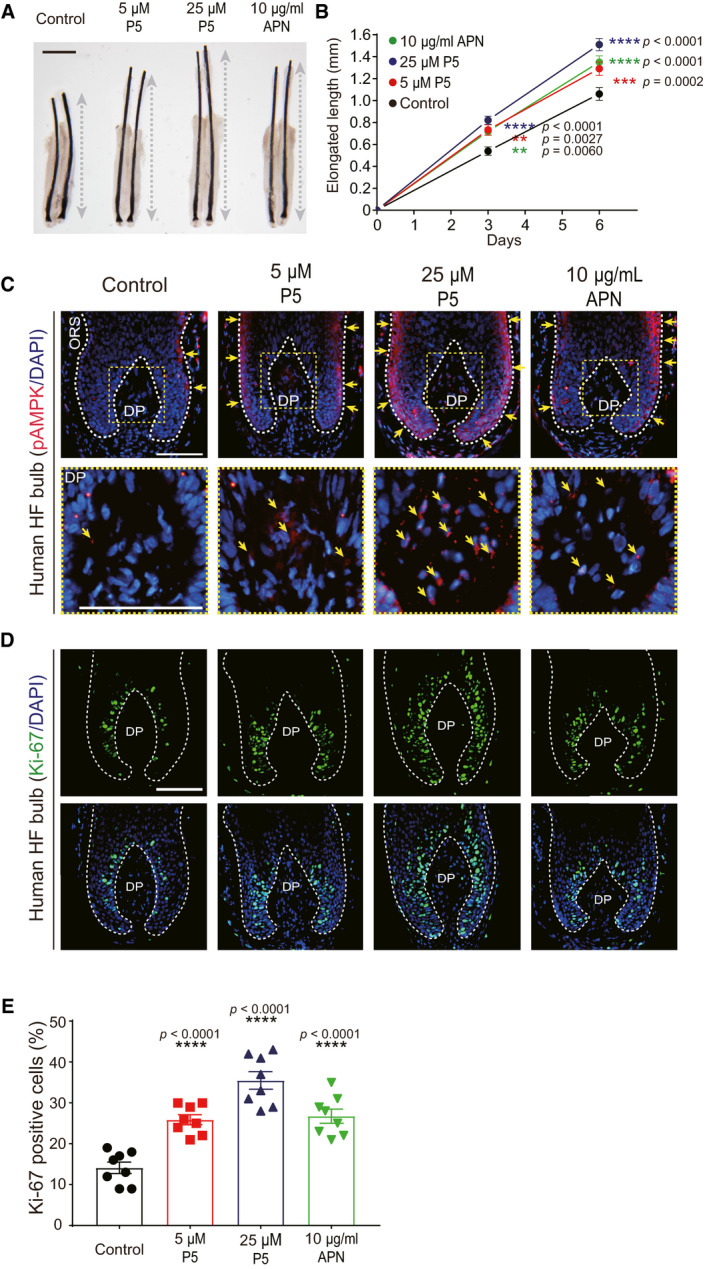
P5 promotes hair growth *ex vivo* *Ex vivo* cultured human HF treated with vehicle, P5, or APN for 6 days; scale bar: 1 mm; dotted gray bar: the length of pigmented hair shaft.The net length of elongated hair shaft is measured and compared to the control group (*n* = 38 HFs from three independent donors/group); Two‐way ANOVA with Dunnett's test compared to the control group.IF staining (p‐AMPK) of the HF bulb area. Yellow arrow indicates p‐AMPK signal; scale bar: 100 µm.IF staining (Ki‐67) of the HF bulb area; scale bar: 100 µm.The numbers of Ki‐67‐positive cells normalized to DAPI‐stained cells (*n* = 8 HFs/group); One‐way ANOVA with Dunnett's test compared to the control group. Data information: In (B and E), data are presented as the mean ± SEM. Source data are available online for this figure.

We next investigated whether P5 can reproduce the effects of APN on hair growth promotion *ex vivo* (Won *et al*, 2012) using *ex vivo* human HF organ culture model. P5 treatment significantly promoted hair shaft growth *ex vivo*, which is comparable to the effects of APN as a positive control (Fig 3A and B). In the immunofluorescence (IF) staining of HF bulb, p‐AMPK was prominently detected in ORS and DP cells of P5‐treated HFs compared to those of vehicle‐treated HFs (Fig 3C). Also, the cell proliferation marker Ki‐67 in matrix cells of *ex vivo* cultured HF bulbs was increased significantly in P5‐treated HFs than in vehicle‐treated HFs (Fig 3D). The Ki‐67‐positive cell portion of the matrix area in the P5‐treated HFs was higher than that in the vehicle‐treated group (Fig 3E). These results clearly demonstrated that P5 can promote hair growth *ex vivo*, which is comparable to APN.

### Topical P5 treatment induced the anagen hair cycle *in vivo*

Based on a series of molecular and biological studies, we confirmed that P5 interacts with AdipoR1 and reproduces the APN‐induced hair growth‐promoting effects *in vitro* and *ex vivo*. Further, we tested whether P5 can accelerate hair growth *in vivo,* using anagen induction assay in mice (Slominski & Paus, 1993; Ohn *et al*, 2019).

**Figure 4:**
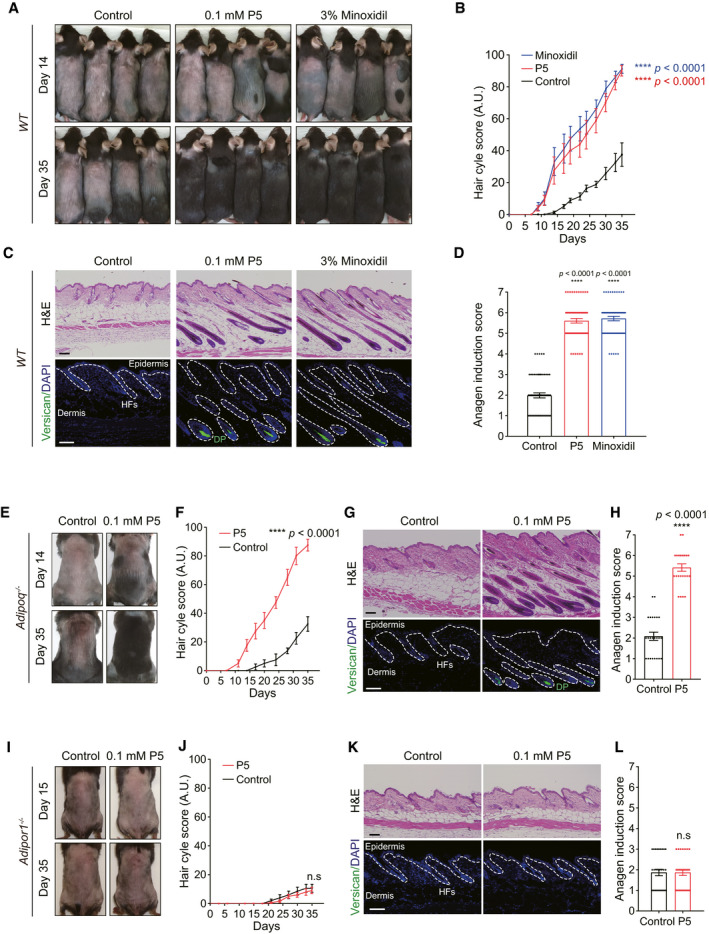
Topical P5 treatment induced the anagen hair cycle through AdipoR1 *in vivo* Gross morphology of vehicle‐, P5‐, or minoxidil‐treated *WT* mice.Hair cycle scores of vehicle‐, P5‐, or minoxidil‐treated *WT* mice (*n* = 16 mice/group); Two‐way ANOVA with Dunnett's test compared to the control group.H&E and IF for versican of skin tissue; scale bars: 100 µm.Anagen induction scores of vehicle‐, P5‐, or minoxidil‐treated *WT* mice (*n* = 4 tissues/mice, four mice/group); One‐way ANOVA with Tukey's test compared to the control group.Gross morphology of vehicle‐, or P5‐treated *Adipoq* ^−/−^ mice.Hair cycle scores of vehicle‐, or P5‐treated *Adipoq* ^−/−^ mice (*n* = 4 mice/group); Two‐way ANOVA.H&E and IF for versican of skin tissue; scale bars: 100 µm.Anagen induction scores of vehicle‐, or P5‐treated *Adipoq* ^−/−^ mice (*n* = 6 tissues/mice, four mice/group); Unpaired *t*‐test.Gross morphology of vehicle‐, or P5‐treated *Adipor1* ^−/−^ mice.Hair cycle scores of vehicle‐, or P5‐treated *Adipor1* ^−/−^ mice (*n* = 5 mice/group); Two‐way ANOVA.H&E and IF for versican of skin tissue; scale bars: 100 µm.Anagen induction scores of vehicle‐, or P5‐treated *Adipor1* ^−/−^ mice (*n* = 6 tissues/mice, five mice/group); Unpaired *t*‐test. Data information: All values are presented as the mean ± SEM. Source data are available online for this figure.

First, we topically applied P5 and minoxidil as a positive control (Messenger & Rundegren, 2004) on wildtype (*WT*) mouse. At day 14, we observed obvious pigmentation and hair in P5‐ or minoxidil‐treated mice dorsum (Fig 4A). Most of P5‐ or minoxidil‐treated mice manifested a black hair coat at day 35; however, the HFs in vehicle‐treated mice had not yet fully entered anagen (Fig 4A). Overall, the hair cycle score was higher in the P5‐treated mice than vehicle‐treated mice (Fig 4B). In histological examination, anagen HFs were noticeable in P5‐ and minoxidil‐treated mice, in contrast to telogen HFs in vehicle‐treated mice (Fig 4C). IF staining showed versican expression in DP cells of P5‐ and minoxidil‐treated mice skin (Fig 4C), suggesting that the HFs are in anagen (Soma *et al*, 2005). Consistently, the anagen induction scores based on the morphology of HFs (Müller‐Röver *et al*, 2001) of P5‐ or minoxidil‐treated mice were significantly higher than those of the vehicle‐treated mice (Fig 4D). Collectively, P5 induces anagen hair cycle *in vivo*.

**Figure EV3:**
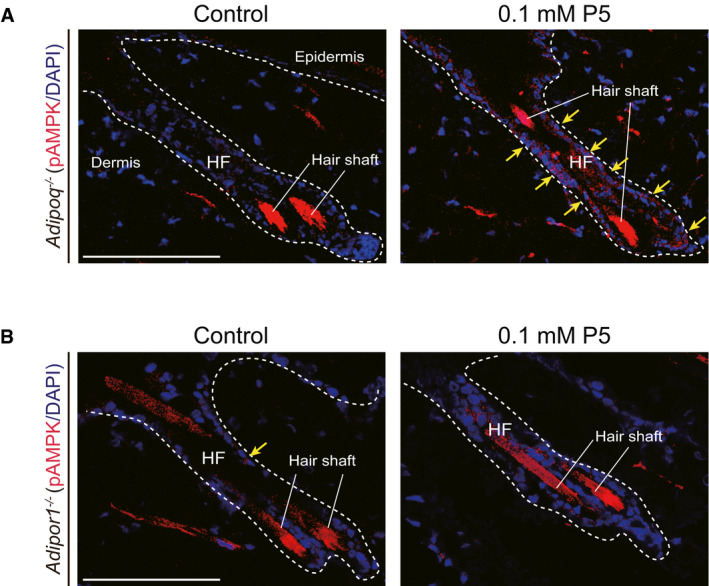
The upregulation of p‐AMPK in HF cells *in vivo* by topical P5 treatment A, Bp‐AMPK in the HF cells of (A) *Adipoq* ^−/−^ mice, and (B) *Adipor1* ^−/−^ mice after P5 treatment. Yellow arrow indicates p‐AMPK signal; scale bar: 100 µm.

We also evaluated P5 effect in *Adipoq* ^−/−^ mouse (Ma *et al*, 2002). After topical P5 treatment, ORS cells showed upregulation of p‐AMPK in telogen HFs of *Adipoq* ^−/−^ mouse (Fig EV3A). Skin pigmentation was prominent in P5‐treated *Adipoq* ^−/−^ mice at day 14 (Fig 4E). In contrast, no obvious pigmentation was observed in vehicle‐treated *Adipoq* ^−/−^ mice (Fig 4E). At day 35, unlike vehicle‐treated *Adipoq* ^−/−^ mice showing little pigmentation, P5‐treated *Adipoq* ^−/−^ mice manifested hairs with pigmentation (Fig 4E). The hair cycle score was significantly different between P5‐ and vehicle‐treated *Adipoq* ^−/−^ mice (Fig 4F). Terminal HFs in anagen were observed in P5‐treated *Adipoq* ^−/−^ mice skin on histological examination, in contrast to in vehicle‐treated *Adipoq* ^−/−^ mice (Fig 4G). The DP cells in P5‐treated *Adipoq* ^−/−^ mice expressed versican (Fig 4G). The anagen induction score was significantly higher in P5‐treated *Adipoq* ^−/−^ mice than vehicle‐treated *Adipoq* ^−/−^ mice (Fig 4H).

To test whether the effect of P5 is mediated through AdipoR1 *in vivo*, we used *Adipor1* ^−/−^ mice (Yamauchi *et al*, 2007; Okada‐Iwabu *et al*, 2013). As a result, p‐AMPK was not increased in HF cells of *Adipor1* ^−/−^ mice after topical P5 treatment (Fig EV3B). Consistently, the anagen induction effects of P5 was abolished in the *Adipor1* ^−/−^ mice (Fig 4I and J), in contrast to in *WT* mouse (Appendix Fig S2). In histological examination, anagen HF was not found in both vehicle‐ and P5‐treated *Adipor1* ^−/−^ mice (Fig 4K and L). Taken together, topical P5 treatment can accelerate hair growth through AdipoR1 *in vivo*.

### Topical P5 can be delivered transdermally in human skin *in vivo*

**Figure EV4:**
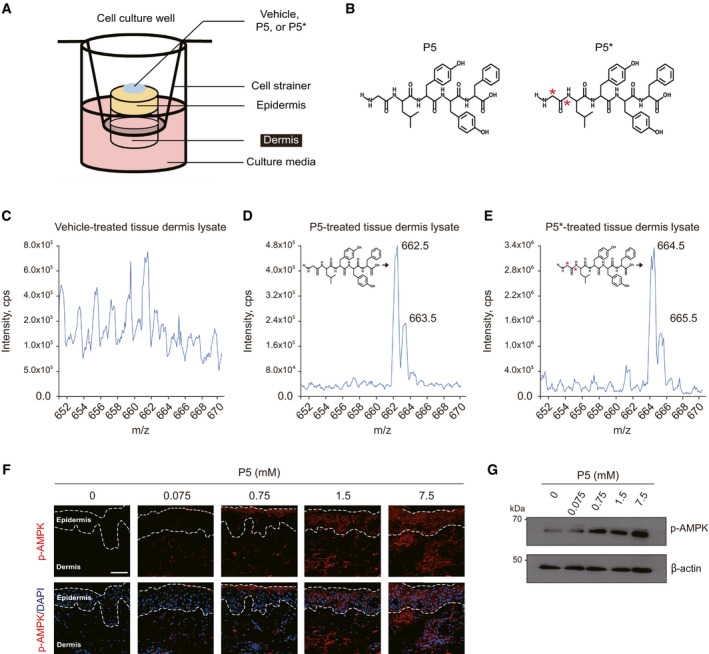
Transdermal delivery of P5 in *ex vivo* and *in vivo* human skin AA schematic illustration of *ex vivo* human skin tissue culture.BThe molecular structure of P5 and isotope‐labeled P5 (P5*); red asterisk indicates isotope carbon (^13^C) atoms.C–EThe qualitative MS data of the dermal lysate from the vehicle‐, P5‐, or P5*‐treated skin tissue, respectively; *m*/*z*, mass‐to‐charge ratio.FIF for p‐AMPK in the P5‐treated human buttock skin tissue *in vivo*; scale bars, 50 µm.GWestern blot for p‐AMPK in the P5‐treated human buttock skin tissue lysate (*n* = 3 biological replicates). Source data are available online for this figure.

Next, we checked whether topically applied P5 can be delivered into human skin using *ex vivo* skin tissue culture model (Fig EV4A) (Haslam *et al*, 2018). After topical application of vehicle or P5 on the *ex vivo* skin tissue, the dermal lysate of each tissue was examined by the qualitative mass spectrometry (MS). P5 was qualitatively detected in the P5‐treated skin dermis at a mass‐to‐charge ratio (*m*/*z*) of 662.5, in contrast, there was no prominent peak in the vehicle‐treated skin dermis (Fig EV4C and D). To trace the origin of P5, we used isotope‐marked P5 (P5*), in which carbon (^12^C) atoms of Gly were substituted with isotope carbon atoms (^13^C) increasing the molecular weight by 2 compared to that of original P5 (Fig EV4B). The qualitative MS using the P5*‐treated skin dermis resulted a high‐intensity peak at *m*/*z* 664.5 (Fig EV4E), which was expected by two isotope carbons in P5*. When P5 was applied on the human skin *in vivo*, it induced the phosphorylation of AMPK in dermis as well as epidermis with a dose‐dependent manner (Fig EV4F), which is also confirmed in the tissue lysates of P5‐treated human skin *in vivo* (Fig EV4G). Collectively, P5 can be transdermally delivered into human skin.

### Structural analysis of the specific binding pocket of AdipoR1 for P5

We next investigated how P5 binds to AdipoR1 at the atomic level by protein‐peptide docking simulation using the GalaxyTongDock (Ko *et al*, 2012; Park *et al*, 2019). To predict the structure of AdipoR1‐P5 complex, the available human AdipoR1 crystal structure (residues 89–364, PDB ID: 3WXV) (Tanabe *et al*, 2015) was used as a starting model. Several models that meet the following three conditions were generated and closely examined: first, both the N‐ and C‐termini of P5 are not buried because P5 is located in the middle of APN; second, the fourth residue Tyr forms a hydrophobic interaction with AdipoR1 because the fourth position is well conserved with aromatic residues; third, the highly conserved residues on the extracellular region of AdipoR1 participate in the binding.

**Figure 5:**
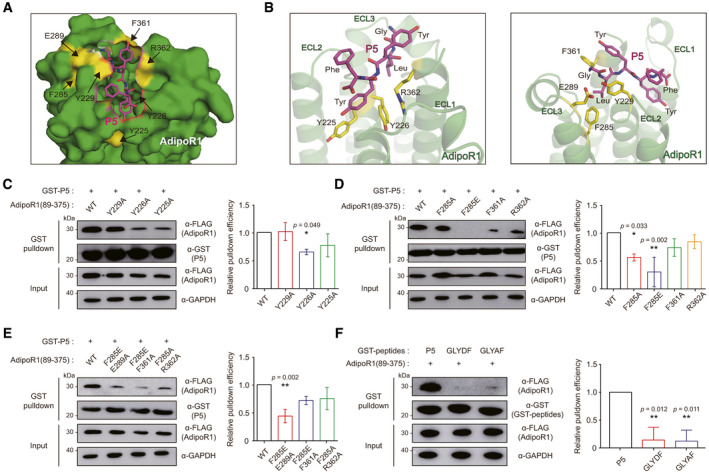
Molecular docking simulation followed by mutational studies AThe most optimal docking model for the AdipoR1‐P5 complex. P5 is shown in magenta sticks. Each amino acid of AdipoR1 in yellow was mutated in the following experiments.BThe magnified view for the detailed AdipoR1‐P5 complex interface (magenta, P5; yellow, amino acids of AdipoR1); ECL, extracellular loop of AdipoR1.C–EPulldown of Flag‐fused AdipoR1 input (residues 89–375) with amino acid mutation(s), as indicated, with GST‐P5. The relative pulldown efficiency is expressed as the ratio of the band intensities of bound AdipoR1 to those of AdipoR1 (residue 89–375) inputs, as indicated (*n* = 3 technical replicates/group).FPulldown of AdipoR1 (residues 89–375) with GST‐P5 or GST fused to two mutant peptides (GLYDF and GLYAF). The relative pulldown efficiency is expressed as the ratio of the band intensities of bound AdipoR1 to those of AdipoR1 (residue 89–375) inputs, as indicated (*n* = 3 technical replicates/group). Data information: In (C–F), data are presented as the mean ± SEM. (One‐way ANOVA with Tukey's test compared to the WT or P5). Source data are available online for this figure.

**Figure EV5:**
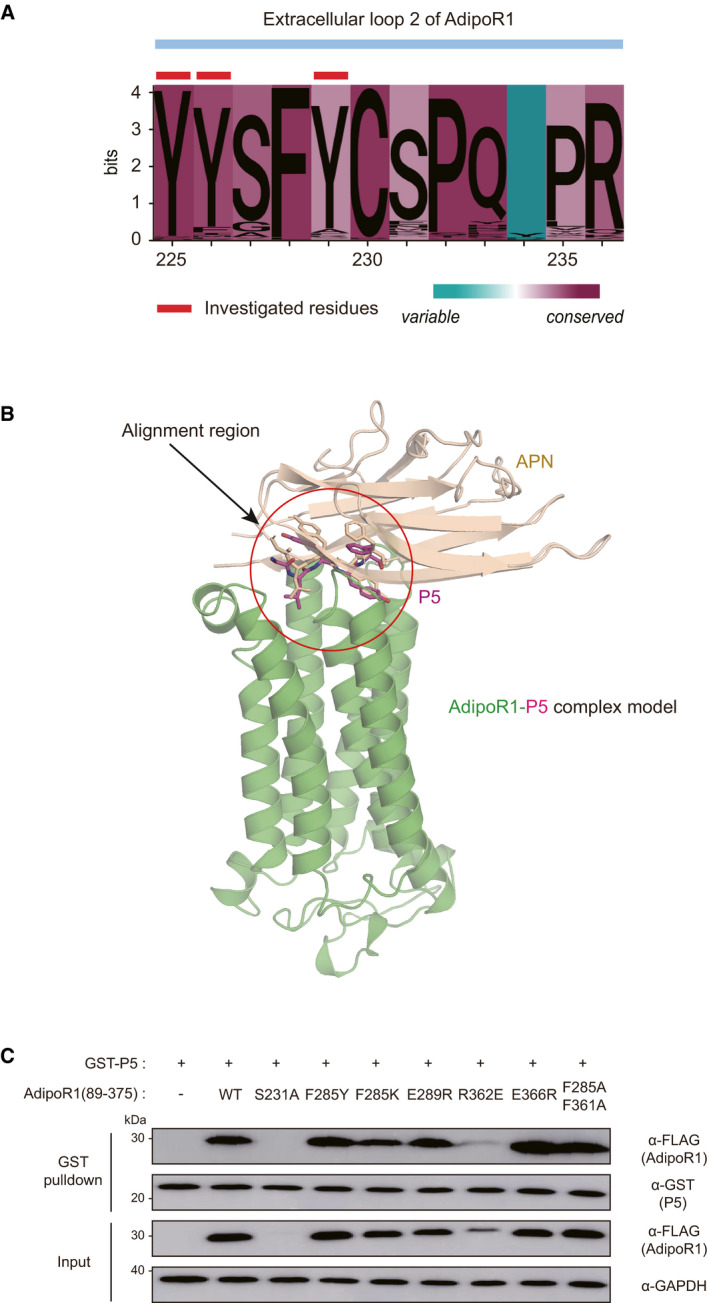
Analysis of the specific binding pocket of AdipoR1 for P5 Sequence logo landscape of MSA of extracellular loop 2 of AdipoR1. The residue numbers are based on the human AdipoR1 amino acid sequence. In a given residue, the height of each letter (measured in bits) is proportionally scaled to the amount of information contributed based on Shannon entropy. The background color of each residue is determined by the degree of conservation in physicochemical properties (cyan to magenta). The investigated residues in the text are marked with red tags (Tyr225, Tyr226, and Tyr229).Cartoon diagram of the superposition of APN with the docking model of the AdipoR1‐P5 complex. The red circle indicates the position of P5. AdipoR1 (PDB ID: 3WXV), APN (PDB ID: 4DOU), and P5 are colored green, wheat, and magenta, respectively.Pulldown of AdipoR1 mutants (residues 89–375) with the GST‐fused P5. Source data are available online for this figure.

Finally, an optimal docking model for the AdipoR1‐P5 complex was selected, in which the protruding side chain of the fourth residue Tyr in P5 is docked to the inner hydrophobic pocket of AdipoR1, forming strong hydrophobic interactions with Tyr225 and Tyr226 in extracellular loop 2 of AdipoR1 protein (Fig 5A and B). Interestingly, these two residues in extracellular loop 2 of AdipoR1 were relatively conserved along with Tyr229, based on the multiple sequence alignment (MSA) results using AdipoR1 sequences (Fig EV5A) (UniProt_Consortium, 2019). Both the N‐ and C‐termini of P5 are surface‐exposed (Fig 5A); thus, APN forms a complex with AdipoR1 without any significant steric clash when the structure of APN was docked in that of the AdipoR1‐P5 complex by superimposing P5. These data confirmed that our AdipoR1‐P5 complex docking model is reasonable (Fig EV5B).

To validate this model, we mutated three residues (Tyr225, Tyr226, and Tyr229) of AdipoR1 and assessed their interactions with P5 by GST pulldown assays. As expected, mutations of the two interfacial residues (Y225A and Y226A) resulted in significant reductions of the pulldown efficiency compared to *WT*, while Y229A mutation showed no reduction (Fig 5C). These results suggest that the hydrophobic pocket of AdipoR1 consisting of Tyr225 and Tyr226 might be important for binding to P5. To examine contributions of other surface‐exposed residues on the extracellular surface of AdipoR1, we generated single or double mutants (F285A, F285E, F361A, R362A, F285E/E289A, F285E/F361A, or F285A/R362A) of AdipoR1 and evaluated their interactions with P5 (Fig 5D and E). Notably, mutations of Phe285 to Ala or Glu significantly reduced the pulldown efficiency, and double mutants of AdipoR1 containing the Phe285 mutation (F285E/E289A, F285E/F361A, or F285A/R362A) also showed significantly reduced pulldown efficiency, implying that Phe285 plays a crucial role in P5 binding (Fig 5D and E). In addition, other two mutants (F361A and R362A) showed mild reduction in their pulldown efficiency for P5 (Fig 5D). However, each pulldown efficiency of P5 for AdipoR1 with the other single mutants (F285Y, F285K, E289R, R362E, or E366R) or double mutants (F285A/F361A) was not decreased (Fig EV5C). For further confirmation of this model, two purified GST‐fused peptides (GLYDF and GLYAF; the fourth Tyr of P5 was mutated into Asp or Ala, respectively) were purified and evaluated for their pulldown efficiency for AdipoR1. As expected, the two GST‐fused peptides showed significantly lower pulldown efficiency for AdipoR1 than P5 (Fig 5F). Taken together, these intensive mutational studies for interfacial residues of AdipoR1 and P5 amply support our docking model.

## Discussion

APN, one of the most important and extensively investigated adipokines (Scherer *et al*, 1995; Hu *et al*, 1996; Maeda *et al*, 1996; Nakano *et al*, 1996; Pajvani *et al*, 2003), binds to the extracellular portion of AdipoR1, activates AMPK signaling pathway, and exerts protective effects on cells (Tomas *et al*, 2002; Yamauchi *et al*, 2002; Kahn *et al*, 2005; Mao *et al*, 2006; Deepa & Dong, 2009; Straub & Scherer, 2019). Low systemic APN level is implicated as a risk factor for many human diseases, such as obesity, obesity‐associated malignancies, metabolic syndrome, cardiovascular disease, and type II diabetes (Berg *et al*, 2002; Chandran *et al*, 2003; Pischon *et al*, 2004; Guerre‐Millo, 2008; Li *et al*, 2009; Straub & Scherer, 2019). In chronic inflammatory skin conditions in humans, including psoriasis, photoaging, and sensitive skin syndrome, the APN level in cutaneous and subcutaneous fat tissue is decreased (Kim *et al*, 2015, 2016; Shibata *et al*, 2015). Taken together, exogenous APN for activating AdipoRs could be beneficial for several human diseases (Brochu‐Gaudreau *et al*, 2010).

However, it is limited to use exogenous APN as an AdipoRs modulator in humans (Otvos, 2019). Instead, many researchers have developed peptide‐based or small molecule‐based AdipoRs activators (Otvos *et al*, 2011, 2014; Okada‐Iwabu *et al*, 2013; Singh *et al*, 2014; Ma *et al*, 2017; Kim *et al*, 2018; Otvos, 2019). An octapeptide identified by screening a sequence‐based library is able to activate AdipoRs (Otvos *et al*, 2011). To improve the stability and solubility, ADP355 consisting of non‐natural residues has been developed (Otvos *et al*, 2014). Beside peptides, agonistic small molecules of AdipoRs, such as AdipoRon and GTDF, have also been developed and investigated widely (Okada‐Iwabu *et al*, 2013; Singh *et al*, 2014). However, it is not elucidated whether the agonists are effective in alleviating alopecia, especially via transdermal delivery route in a topical form. It is crucial that a therapeutic strategy for alopecia should effect only in the intended area locally to minimize unwanted systemic side effects.

In this study, we confirmed that the transdermally deliverable P5 activates AMPK signaling pathway through AdipoR1 increasing hair growth factor expression in human DP *in vitro* and promotes hair growth in *ex vivo* HF organ culture. Furthermore, P5 treatment induced anagen hair cycle in *in vivo* mouse models, which is mediated by AMPK signaling pathway, a downstream effector of AdipoR1 (Mao *et al*, 2006; Deepa & Dong, 2009). Collectively, P5 is a promising drug candidate which is a topically applicable molecule for alleviating alopecia. In further, human clinical trials are necessary to utilize topical P5 application for the treatment of alopecia patients in the clinical setting.

Furthermore, our study is noteworthy in that the agonistic structure of APN binding to AdipoR1 is specified into pentapeptide, along with revealing the key spot of AdipoR1. The fact that the key spot of AdipoR1 for APN has been largely unknown hinders the development of effective drug molecules targeting AdipoR1. Based on our docking studies followed by extensive mutational studies, the inner hydrophobic cleft of AdipoR1 consisting of Tyr225 and Tyr226 is suggested to be a crucial region for activating AdipoR1 upon the agonist binding. This newly identified key spot might be useful to further design AdipoR1 activating molecules for various biological beneficial consequences, given that upregulating AdipoR1 would result positive impacts on aforementioned human diseases.

In summary, we successively narrowed down the sequences of globular APN into the pentapeptide (GLYYF: P5), which can bind to and activate AdipoR1. Based on the comprehensive molecular and biological evidences, we showed its efficacy on hair growth promotion through AdipoR1 signaling pathway after topical application. Moreover, our findings of the crucial region in AdipoR1 for binding to P5 pave the way for the development of novel AdipoR1 modulating molecules in the future.

## Materials and Methods

### Ethics statement

All human research protocols conformed to the ethical principles of the WMA Declaration of Helsinki, and written informed consent was obtained from all human subjects. The protocols were approved by the Institutional Review Board of the Seoul National University Hospital (2003‐031‐1109 and 1603‐114‐750). The experiments conformed to the principles set out in the Department of Health and Human Services Belmont Report. All animal procedures were performed under the American Association for the Accreditation of Laboratory Animal Care guidelines and approved by the Institutional Animal Care and Use Committee of the Seoul National University Hospital (20‐0037).

### Protein sequence database, MSA, conserved score calculation, and structure visualization

The amino acid sequence data of APN (372 amino acid sequences from 132 species) and AdipoR1 (213 amino acid sequences from 177 species) were obtained from UniProtKB through the website https://www.uniprot.org/ (UniProt_Consortium, 2019). MSA of the protein sequences was created using Clustal Omega (Sievers *et al*, 2011). The sequence logos were illustrated using Ggseqlogo (Wagih, 2017). The degree of conservation of the physicochemical properties was calculated based on the previous literature (Livingstone & Barton, 1993), with background colored by conservation degree according to conditional formatting percentile rank among APN globular domain or AdipoR1 sequences. All structures of APN and AdipoR1 were visualized using PyMOL (PyMOL Molecular Graphics System, https://pymol.org/).

### Pulldown assay

For analysis of the interaction of P5 to AdipoR1, the cDNAs encoding full‐length AdipoR1 (residues 1–375) and N‐terminal‐truncated AdipoR1 (residues 89–375) were cloned into the p3×Flag_CMV vector. Transfected HEK293T cells were lysed in lysis buffer (Tris–HCl pH 8.0 [20 mM], NaCl [0.2 M], Triton X‐100 [1%], PMSF [1 mM], aprotinin [4 µg/ml], leupeptin [10 µg/ml], and pepstatin [1 µg/ml]). GST‐fused P5 and P10 were expressed in *Escherichia coli* strain BL21 (DE3) cells. For the interaction analysis, individually purified GSTfused P5 or P10 was incubated with glutathione–Sepharose beads (20 µl), AdipoR1‐overexpressing cells were added to the beads, and the mixtures were gently rotated at 4°C overnight. After the beads were washed three times with phosphate‐buffered saline containing Tween‐20 (PBST) buffer (Na_2_HPO_4_ [10 mM], KH_2_PO_4_ [1.8 mM], KCl [2.7 mM], NaCl [137 mM], Tween‐20 [pH 7.4, 0.05% v/v]), the bound protein was eluted with 20 µl of PBST buffer supplemented with glutathione (20 mM). Input and eluate samples were separated by SDS–PAGE, analyzed by Western blot using specific antibodies; DYKDDDDK tag mouse mAb (#8146, 1:1,000; Cell Signaling) and GST mouse mAb (#2624, 1:1,000; Cell Signaling) as primary antibody. Anti‐mouse IgG HRP‐linked antibody (#7076, 1:5,000; Cell Signaling) was used for secondary antibody. The membranes were visualized with enhanced chemiluminescent detection (#34580; Thermo Fisher). All constructs used in pulldown experiments were made by site‐directed mutagenesis (Appendix Table S1).

### Molecular docking of P5 to AdipoR1

We used GalaxyTongDock for docking the P5 to AdipoR1 (Ko *et al*, 2012; Park *et al*, 2019). GalaxyTongDock performs an exhaustive sampling of docking poses treating both protein and peptide as rigid structures by a fast Fourier transformation with improved energy parameters. The rigid body docking approach was employed here, assuming that the binding mode of P5 to AdipoR1 is similar to that of APN to the same receptor. The starting structure of AdipoR1 was obtained from the Protein Data Bank (PDB ID: 3WXV) (Tanabe *et al*, 2015), with deletion of the flexible C‐terminal region at 365–373. P5 structure was extracted from that of APN (PDB ID: 4DOU) (Min *et al*, 2012). The transmembrane and cytoplasmic regions of AdipoR1 were excluded using the block option of GalaxyTongDock. The predicted docking poses were then relaxed by GalaxyRefineComplex (Heo *et al*, 2016).

### Isolation of human HFs and HF constituent cells

Human scalp tissue (sized 1.0 × 1.5 cm) was obtained from the occipital area of healthy volunteers without any cutaneous disease history of the scalp. Human HFs were isolated from the scalp tissue and dissected into single follicular units with No. 20 scalpel blades under a dissecting stereomicroscope (Olympus). Micro‐dissected human HFs in anagen stage VI were used for the HF organ culture, primary DP cell, and ORS cell culture and subsequent analysis.

### Cell culture of HEK293T, ORS, and DP cells

HEK293T cells (Riken) were cultured in Dulbecco's modified Eagle's medium (DMEM; Caisson) containing 5% fetal bovine serum (FBS; Welgene), l‐glutamine (20 mM), and penicillin‐streptomycin (1%, Thermo Fisher). The isolation of human ORS and DP cells was performed as previous study (Messenger, 1984; Limat & Noser, 1986). In brief, for ORS cell culture, the bulge region of the dissected human HFs was cutoff to prevent contamination with other cells. The trimmed bulge region was immersed in DMEM (#LM 001‐05, Welgene) supplemented with 20% FBS. On the third day of culture, the medium was changed to Epilife medium (#MEPI500CA, Gibco) supplemented with human keratinocyte growth supplement (#S0015, Gibco). For DP cell culture, the proximal parts of the HF (bulb area) were removed using a needle, and DP was isolated from the tip of hair bulbs. The DPs were immersed in DMEM supplemented with 20% FBS. The explanted DPs were left for 14 days. Then, the medium (DMEM supplemented with 10% FBS) was changed every two days. All cells were cultured at 37°C in a 5% CO_2_ atmosphere.

### 3‐(4,5‐dimethylthiazol‐2‐yl)‐2,5‐diphenyltetrazolium bromide (MTT assay)

Dermal papilla cells and ORS cells were seeded into a 96‐well plate (1.0 × 10^4^ cells/well) with serum‐free culture media for 24 h and then treated with each candidate 5‐mer peptide (in 0.5 μg/ml or 5 μg/ml) for 48 h. After the addition of MTT solution (20 μl, 5 mg/ml) to each well, the cells were incubated for 4 h at 37°C in the dark room and then incubated with dimethyl sulfoxide (DMSO, 200 μl) for 20 min at room temperature. The samples were assessed by measuring the absorbance at 570 nm with an absorbance microplate reader (VersaMax).

### RNA interference for AdipoR1

All transfections were performed using Lipofectamine 2000 following the manufacturer’s instructions (#11668019; Thermo Fisher). Human DP and ORS cells were cultured without antibiotics until reaching 50% confluency. AdipoR1 or scrambled siRNA (Bioneer) was used. The culture medium was changed 6 h after transfection, and the cells were incubated for another 24 h before subsequent analyses.

### Western blot analysis

Human ORS and DP cells or skin tissue were lysed (RIPA lysis buffer; #20‐188; Merck Millipore). The obtained protein was then separated by 8% SDS–PAGE and transferred to a polyvinylidene fluoride membrane (Amersham). The protein transferred membranes were incubated overnight with the specific antibodies. The following antibodies were used: β‐actin (#MA5‐15739, 1:5,000; Thermo Fisher), phospho‐ERK1/2 (#9101, 1:1,000; Cell Signaling), total ERK1/2 (#9102, 1:1,000; Cell Signaling), phospho‐AMPK (#2535, 1:1,000; Cell Signaling), and total AMPK (#2532, 1:1,000; Cell Signaling). Then, the membranes were washed and incubated with anti‐rabbit IgG or anti‐mouse IgG antibodies (horseradish peroxidase‐conjugated, GTX213110, GTX213111, 1:10,000; GeneTex) at 25°C for 1 h. Antibody‐antigen complexes detected by enhanced chemiluminescent substrate (Thermo Fisher) were captured and quantified by Amersham imager 680 systems (GE Healthcare).

### Quantitative real‐time reverse transcription–PCR analysis

Total RNA was isolated from P5‐treated DP cells using RNAiso Plus (#9109; TaKaRa). We used the total RNA (1 µg) for cDNA synthesis using a RevertAid First Strand cDNA Synthesis Kit (Thermo Fisher) according to the manufacturer's instructions. The mRNA expression levels were quantified on a 7500 Real‐time PCR System (Thermo Fisher) using TB Green Premix Ex Taq (TaKaRa Bio): human *IGF‐1, VEGF, HGF, PDGFA, FGF7*, and glyceraldehyde 3‐phosphate dehydrogenase (*GAPDH*) (Appendix Table S2). The relative expression levels of each gene were determined by the $2^{‐\Delta \Delta \text{CT}}$ method.

### Growth factor protein quantitation

Human DP cells were grown on a 100‐mm cell culture dish to 100% confluency. After washed by PBS three times, the cells were cultured with serum‐free DMEM and treated with P5. After 48 h, the medium was collected and filtered by a 0.22‐mm filter. The filtrate was then concentrated using a centrifugal concentrator (Vivaspin 6, 10‐kDa molecular weight cutoff, centrifuged 2,600 *g* at 4°C for 13 min, Sartorius Stedim Biotech). The signals of each protein in the concentrated media were detected and quantified, based on the enzyme‐linked immunosorbent assay (Human Cytokine Antibody Array C2000, RayBiotech).

### Human HF organ culture

The microdissected full‐length scalp HFs in anagen VI were cut at the level of sebaceous duct and then *ex vivo* cultured for 6 days at 37°C in a 5% CO_2_ atmosphere in Williams E medium (#12551032, Gibco) supplemented with hydrocortisone (10 ng/ml), insulin (10 µg/ml), l‐glutamine (2 mM), and penicillin‐streptomycin solution (1×). To avoid any potential bias in the length measurement from pseudo‐catagen progression, the total length of pigmented hair shaft of each HF was measured every three days to calculate the net length of elongated hair shaft and photographed in culture day 6 using a stereomicroscope (Olympus).

### Histology and IF staining

Paraffin‐embedded mouse cutaneous back tissue was sectioned (4 μm thickness) and stained with hematoxylin and eosin. For IF staining, human ORS cells cultured in FiTC‐P5, sectioned paraffin‐embedded human HFs (7 μm thickness), and sectioned paraffin‐embedded mouse cutaneous dorsal tissue or human skin tissue (5 μm thickness) were incubated at 4°C overnight with the following primary antibodies diluted in the diluent reagent (Invitrogen): phospho‐AMPK (#2535, 1:100; Cell Signaling), Ki‐67 (#M7240, 1:200; Dako), versican (#ab177480, 1:200; Abcam), or AdipoR1 antibody (#ab126611, 1:200; Abcam). After three washes with PBS, the slides were incubated with the secondary Alexa Fluor 488‐labeled anti‐mouse or rabbit IgG antibody (#ab150077, #ab150113, 1:200; Invitrogen), or Alexa Fluor 594‐labeled anti‐rabbit IgG antibody (#a11012, 1:200; Invitrogen) at 25°C for 1 h. Nuclei were counterstained with 4′,6‐diamidino‐2‐phenylindole (DAPI; #D1306, 1:1,000; Invitrogen). Microscopic observations were performed with a Nikon Eclipse Ci‐L microscope (Nikon). IF intensity was observed and recorded with a Nikon Eclipse Ni‐E fluorescence microscope (Nikon).

### Mouse hair cycle modulation

For the hair cycle modulation experiment, *Adipoq* ^−/−^ (stock #008195; JAX) (Ma *et al*, 2002), *Adipor1* ^−/−^ (Yamauchi *et al*, 2007; Okada‐Iwabu *et al*, 2013), or C57BL/6 (KOATECH) female mice was used (Müller‐Röver *et al*, 2001). They were housed (up to four animals per cage; 23 ± 2°C, 8:00–20:00, 12 h/12 h light/dark cycle). Mice were fed a standard chow diet and provided food and water *ad libitum*. Mice were randomly allocated and shaved dorsally at 7.5 weeks of age. Only mice in telogen were included for the further experiments (Müller‐Röver *et al*, 2001). From 8 weeks of age, the shaved dorsal area was treated with vehicle control (ethanol and polyethylene glycol, 30% and 70% v/v), P5 (0.1 mM), or minoxidil (3%) every day. The mouse hair growth score (a value from 0 to 100 based on the skin pigmentation and hair shaft density) was monitored and documented at designated days with the experimenters being blind to the conditions, following a previous study (Chai *et al*, 2019). A higher score corresponds to a transition from telogen to anagen. On day 35, we obtained dorsal cutaneous tissue for histological analysis: anagen induction scores (calculated using an assigned arbitrary score; telogen = 1, anagen I–VI = 2–7) (Müller‐Röver *et al*, 2001).

### *Ex vivo* culture of human skin tissue

Topical application of peptides on *ex vivo* skin study was performed as described previously (Haslam *et al*, 2018). Briefly, full thickness skin tissue from cadavers within 48 h after death was acquired and biopsied using a 6 mm punch. The skin tissue was acclimatized in supplemented Williams’ E media with l‐glutamine (2 mM), penicillin‐streptomycin solution (1×), hydrocortisone (10 ng/ml), and insulin (100 ng/ml) overnight at 37°C in a 5% CO_2_ atmosphere. After acclimatization, the skin tissue was carefully pulled through a cell strainer with the epidermis exposed to the air, leaving the dermis submerged in the culture media. Vehicle (DMSO), P5 (50 mM), or P5* (50 mM) was placed on the center of the skin sample daily and cultured for 5 days. Then, the dermal layer of the cultured skin tissue was carefully isolated and homogenized in 50% methanol, which resulted in the dermal lysate. The lysate was qualitatively analyzed by a MS system (API 4000 Q‐trap; AB SCIEX).

### Topical application of P5 on human *in vivo*

Each designated concentration of P5 (0.075, 0.75, 1.5, or 7.5 mM) was prepared in vehicle (30% ethanol and 70% polyethylene glycol, v/v). The vehicle and four concentrations of P5 were topically applied on the left buttock of human and occluded. After 24 h, we acquired full thickness skin tissue using a 6 mm punch for subsequent analysis.

### Statistical analysis

Statistical analysis was performed using *t*‐test, one‐way analysis of variance (ANOVA) with a *post hoc* test, or two‐way ANOVA with a *post hoc* test as designated. All tests were two‐tailed, and *P*‐value < 0.05 was considered significant. The animals/samples were randomly allocated into experimental groups. The data evaluation from animal experiment was performed by the researchers being blind to the conditions.

## Author contributions

Conceptualization: JYK, JO, KWB, OK, JHC, and HHL; Methodology and investigation: EJK, JYK, JO, KWB, CS, MO‐I, MI, TY, OK, JHC, and HHL); Data curation and formal analysis: JYK, KWB, JO, TP, H‐JY, JSJ, MO‐I, MI, TY, YKK, KHK, JHC, and HHL; Software: JSJ, CS; Writing – original draft: KWB, JO, JYK, JHC, and HHL; Visualization: KWB, JO; Writing – review & editing: KWB, JO, JYK, EJK, JHC, and HHL; Supervision, project administration and funding acquisition: JHC and HHL.

## Conflict of interest

The authors declare that they have no conflict of interest.

## For more information

OMIM website of ADIPOQ at https://www.omim.org/entry/605441

## Supporting information



Appendix

Expanded View Figures PDF

Source Data for Expanded View/Appendix

Source Data for Figure 1

Source Data for Figure 2

Source Data for Figure 3

Source Data for Figure 4

Source Data for Figure 5

## Data Availability

This study includes no data deposited in external repositories. The data that support the findings of this study are available from the corresponding author upon reasonable request.
