# Supplementary material for: Discovery of a transdermally deliverable pentapeptide for activating AdipoR1 to promote hair growth
Source: EMBO Mol Med. 2021 Sep 6;13(10):e13790. doi: 10.15252/emmm.202013790 (PMC8495455; doi:10.15252/emmm.202013790)
Supplement: Supplementary file 1 — Appendix [file EMMM-13-e13790-s006.pdf]

Table of contents

Appendix Figure S1 - siRNA efficiency for *AdipoR1*

Appendix Figure S2 - Topical P5 treatment on *Adipor1*$^{-/-}$ and *WT* mice

Appendix Table S1 - Sequences for site-directed mutagenesis

Appendix Table S2 - Primer sequences for RT-PCR

Appendix Figure

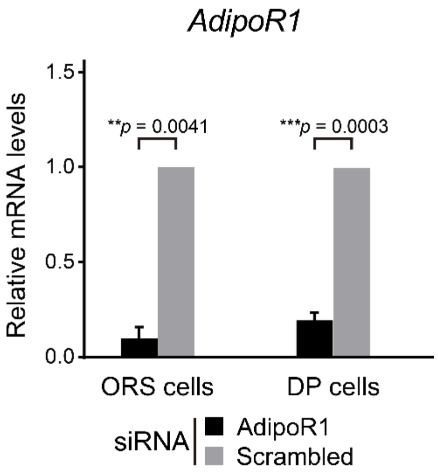

Appendix Figure S1 - siRNA efficiency for *AdipoR1*

The siRNA efficiency for *AdipoR1* in ORS cells ($n = 3$) and DP cells ($n = 4$)

Data information: All values are presented as the mean ± SEM. Statistical significance was determined using Welch's t test (** $p < 0.01$ and ***$p < 0.001$ compared to each scrambled siRNA treated group).

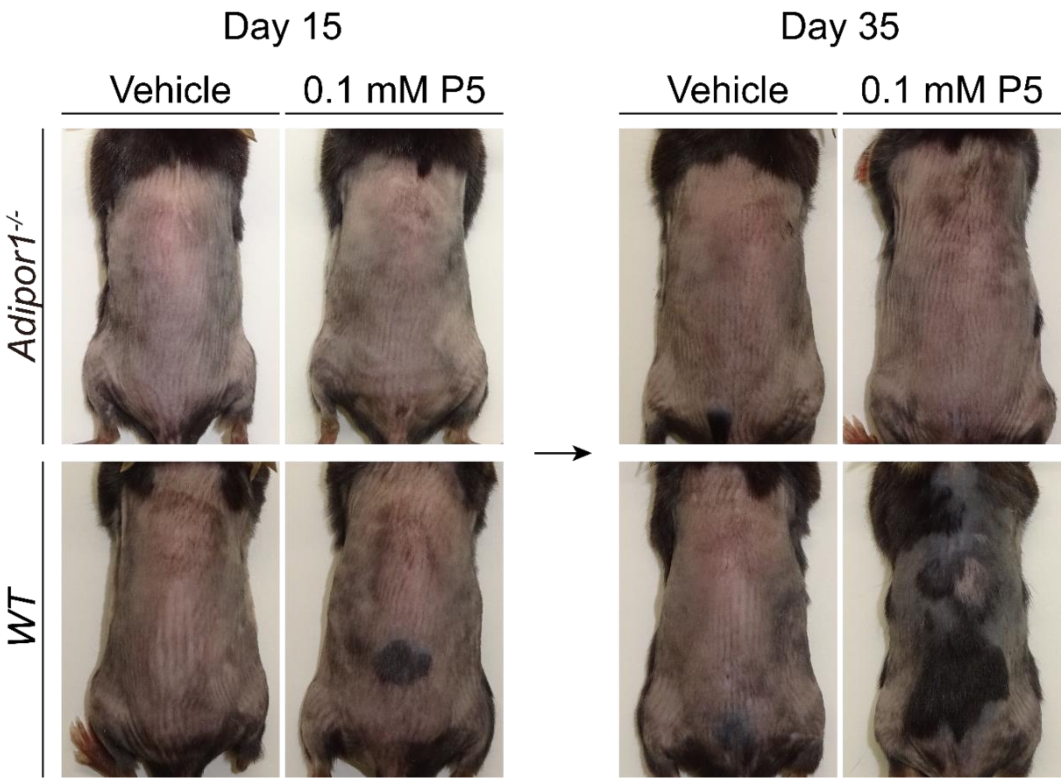

Appendix Figure S2 - Topical P5 treatment on *Adipor1*$^{-/-}$ and *WT* mice

In *Adipor1*$^{-/-}$ mice, anagen hair cycle is not induced by P5 treatment.

Appendix Table S1 - Sequences for site-directed mutagenesis

**AdipoR1 (S231A)**	
forward	TCTATTATTCCTTCTACTGCGCTCCACAGCCACGGCTCATCTA
reverse	TAGATGAGCCGTGGCTGTGGAGCGCAGTAGAAGGAATAATAGA
**AdipoR1 (Y225A)**	
Forward	GGAGCTTTGTCCCCTGGCTCGCTTATTCCTTCTACTGCTCCCC
Reverse	GGGGAGCAGTAGAAGGAATAAGCGAGCCAGGGGACAAAGCTCC
**AdipoR1 (Y226A)**	
Forward	GCTTTGTCCCCTGGCTCTATGCTTCCTTCTACTGCTCCCCACA
Reverse	TGTGGGGAGCAGTAGAAGGAAGCATAGAGCCAGGGGACAAAGC
**AdipoR1 (Y229A)**	
Forward	CCTGGCTCTATTATTCCTTCGCTTGCTCCCCACAGCCACGGCT
Reverse	AGCCGTGGCTGTGGGGAGCAAGCGAAGGAATAATAGAGCCAGG
**AdipoR1 (F285A)**	
Forward	GCGTCGTGCCCACCATGCACGCTACTATCGCTGAGGGCTTTGT
Reverse	ACAAAGCCCTCAGCGATAGTAGCGTGCATGGTGGGCACGACGC
**AdipoR1 (F285E)**	
Forward	GCGTCGTGCCCACCATGCACGAGACTATCGCTGAGGGCTTTGT
Reverse	ACAAAGCCCTCAGCGATAGTCTCGTGCATGGTGGGCACGACGC
**AdipoR1 (F285Y)**	
forward	GCGTCGTGCCCACCATGCACTACACTATCGCTGAGGGCTTTGT
reverse	ACAAAGCCCTCAGCGATAGTGTAGTGCATGGTGGGCACGACGC
**AdipoR1 (F285K)**	
forward	GCGTCGTGCCCACCATGCACAAGACTATCGCTGAGGGCTTTGT
reverse	ACAAAGCCCTCAGCGATAGTCTTGTGCATGGTGGGCACGACGC
**AdipoR1 (E289A)**	
forward	CCATGCACTTTACTATCGCTGCTGGCTTTGTCAAGGCCACCAC
reverse	GTGGTGGCCTTGACAAAGCCAGCAGCGATAGTAAAGTGCATGG
**AdipoR1 (E289R)**	
forward	CCATGCACTTTACTATCGCTCGCGGCTTTGTCAAGGCCACCAC
reverse	GTGGTGGCCTTGACAAAGCCGCGAGCGATAGTAAAGTGCATGG
**AdipoR1 (F361A)**	
forward	GAGTCTCCAACCTTCAGGAAGCTCGTTACGGCCTAGAAGGCGG
reverse	CCGCCTTCTAGGCCGTAACGAGCTTCCTGAAGGTTGGAGACTC
**AdipoR1 (R362A)**	
forward	TCTCCAACCTTCAGGAATTCGCTTACGGCCTAGAAGGCGGCTG
reverse	CAGCCGCCTTCTAGGCCGTAAGCGAATTCCTGAAGGTTGGAGA
**AdipoR1 (R362E)**	
forward	TCTCCAACCTTCAGGAATTCGAGTACGGCCTAGAAGGCGGCTG
reverse	CAGCCGCCTTCTAGGCCGTACTCGAATTCCTGAAGGTTGGAGA
**AdipoR1 (E366A)**	
forward	AGGAATTCCGTTACGGCCTAGCTGGCGGCTGTACTGATGACAC
reverse	GTGTCATCAGTACAGCCGCCAGCTAGGCCGTAACGGAATTCCT
**AdipoR1 (F285A/E289A)**	
forward	GCGTCGTGCCCACCATGCACGCTACTATCGCTGCTGGCTTTGT
reverse	ACAAAGCCAGCAGCGATAGTAGCGTGCATGGTGGGCACGACGC
**AdipoR1 (F285E/E289A)**	
forward	GCGTCGTGCCCACCATGCACGAGACTATCGCTGCTGGCTTTGT
reverse	ACAAAGCCAGCAGCGATAGTCTCGTGCATGGTGGGCACGACGC

Appendix Table S2 - Primer sequences for RT-PCR

**Human IGF-1**	
forward	TTC AAC AAG CCC ACA GGG
reverse	GGT GCG CAA TAC ATC TCC
**Human VEGFA**	
forward	ACT TCT GGG CTG TTC TCG
reverse	TCC TCT TCC TTC TCT TCT TC
**Human HGF**	
forward	CGC AGC TAC AAG GGA ACA GT
reverse	TCC TGT AGG TCT TTA CCC CGA
**Human PDGFA**	
forward	GCC CAT TCG GAG GAA GAG
reverse	TTG GCC ACC TTG ACG CTG CG
**Human FGF-7**	
forward	TTG TGG CAA TCA AAG GGG TG
reverse	CCT CCG TTG TGT GTC CAT TT
