# Supplementary material for: Discovery of a transdermally deliverable pentapeptide for activating AdipoR1 to promote hair growth
Source: EMBO Mol Med. 2021 Sep 6;13(10):e13790. doi: 10.15252/emmm.202013790 (PMC8495455; doi:10.15252/emmm.202013790)
Supplement: Supplementary file 2 — Expanded View Figures PDF [file EMMM-13-e13790-s007.pdf]

Expanded View Figures

Figure EV1. Excavation of the most conserved residues in APN globular domain, and the screening of candidate 5-mer peptides based on DP and ORS cell proliferation.

A Sequence logo landscape of MSA of APN globular domain. The residue numbers are based on the human APN amino acid sequence. In a given residue, the height of each letter (measured in bits) is proportionally scaled to the amount of information contributed based on Shannon entropy. The background color of each residue is determined by the degree of conservation of physicochemical properties (cyan to magenta). The most conserved residues (marked with purple tags) are located within a highly conserved sequence (designated in a green box).

B Candidate 5-mer peptide sequences derived from a highly conserved sequence of APN globular domain. Each sequence is indicated in black.

C DP and ORS cells are used to screen the candidate 5-mer peptides in terms of the cell proliferation effect (n = 3–5 biological replicates/group).

Data are presented as mean ± SEM. Statistical significance was determined using Paired t-test (*$P < 0.05$ or ***$P < 0.001$ compared to the control group).
Source data are available online for this figure.

© 2021 The Authors

A

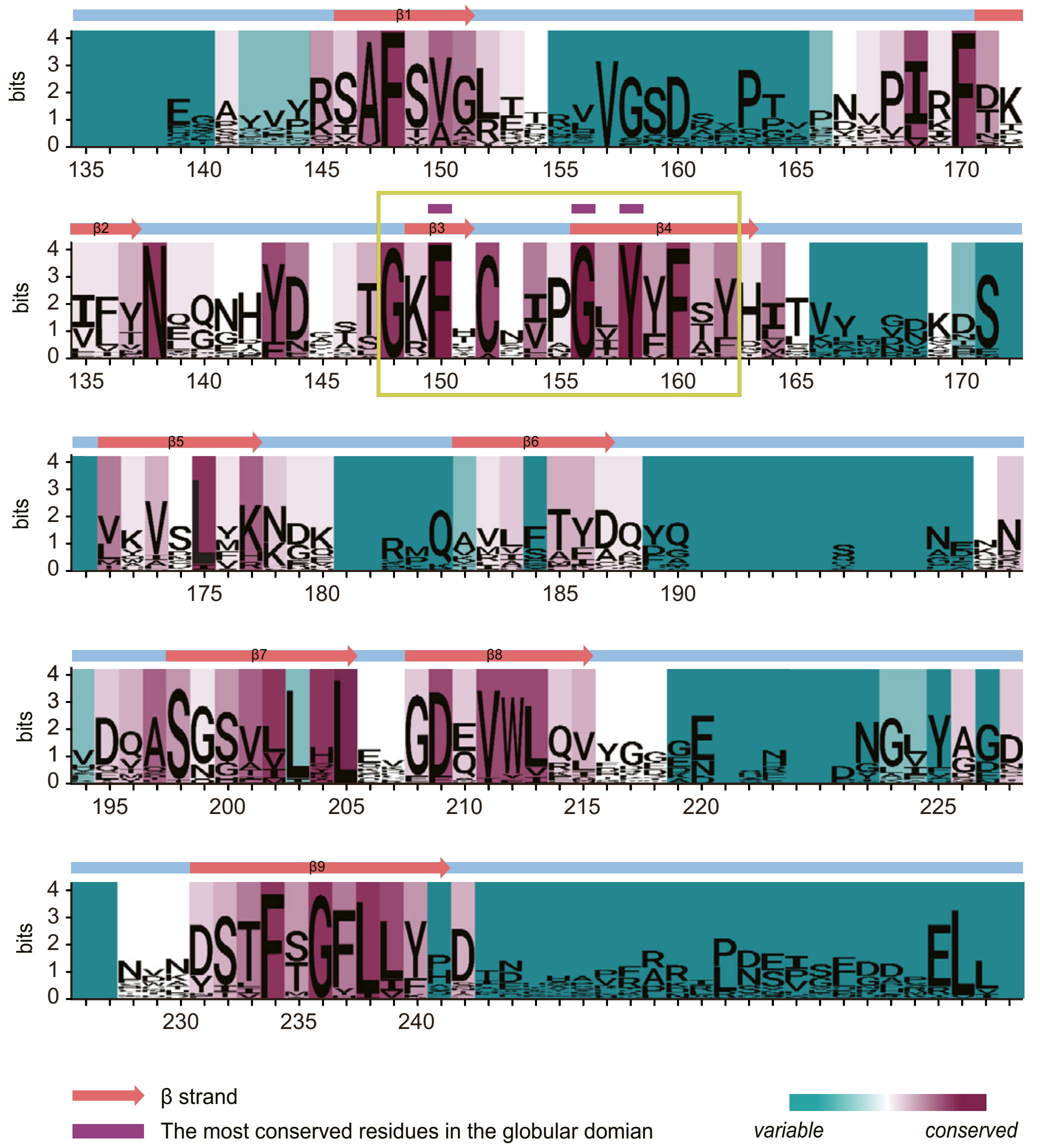

B

APN 5-mer peptides	Peptide sequence
P3	G-K-F-H-C-N-I-P-G-L-Y-Y-F-A-Y
P4	G-K-F-H-C-N-I-P-G-L-Y-Y-F-A-Y
P5	G-K-F-H-C-N-I-P-G-L-Y-Y-F-A-Y
P6	G-K-F-H-C-N-I-P-G-L-Y-Y-F-A-Y
P7	G-K-F-H-C-N-I-P-G-L-Y-Y-F-A-Y
P8	G-K-F-H-C-N-I-P-G-L-Y-Y-F-A-Y
P9	G-K-F-H-C-N-I-P-G-L-Y-Y-F-A-Y
P10	G-K-F-H-C-N-I-P-G-L-Y-Y-F-A-Y

C

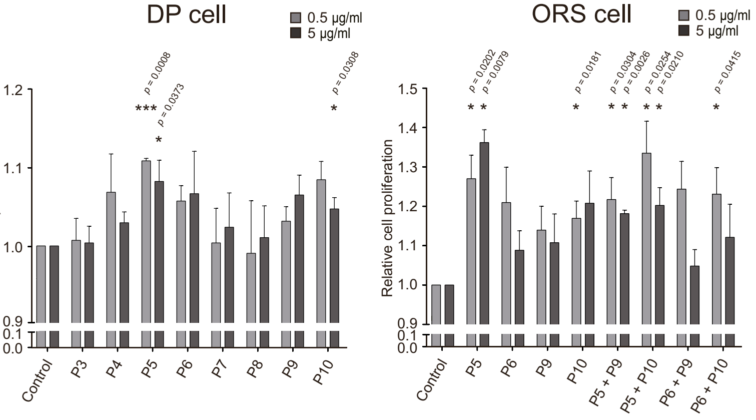

Figure EV1.

© 2021 The Authors

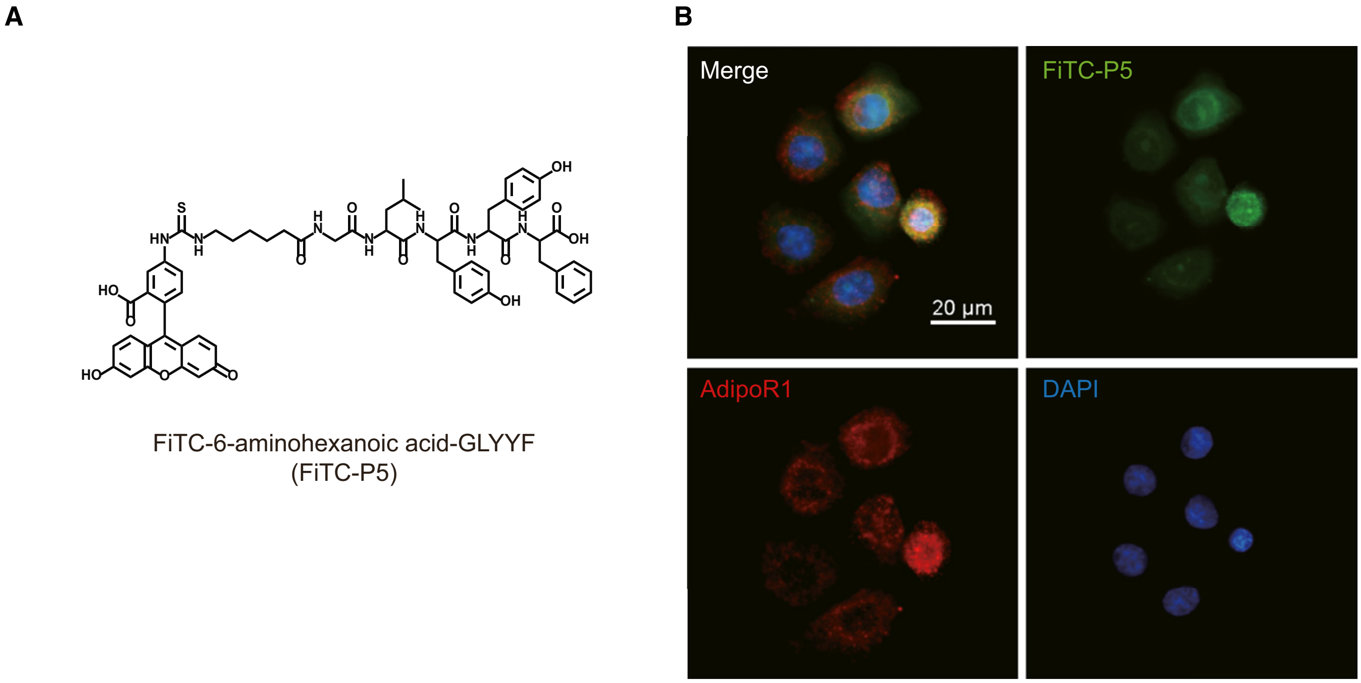

Figure EV2. The colocalization of P5 and AdipoR1 *in vitro*.

A Molecular structure of the FiTC-conjugated P5 (FiTC-P5).
B IF staining for AdipoR1 and FiTC-P5 in cultured FiTC-P5-treated ORS cells.

© 2021 The Authors

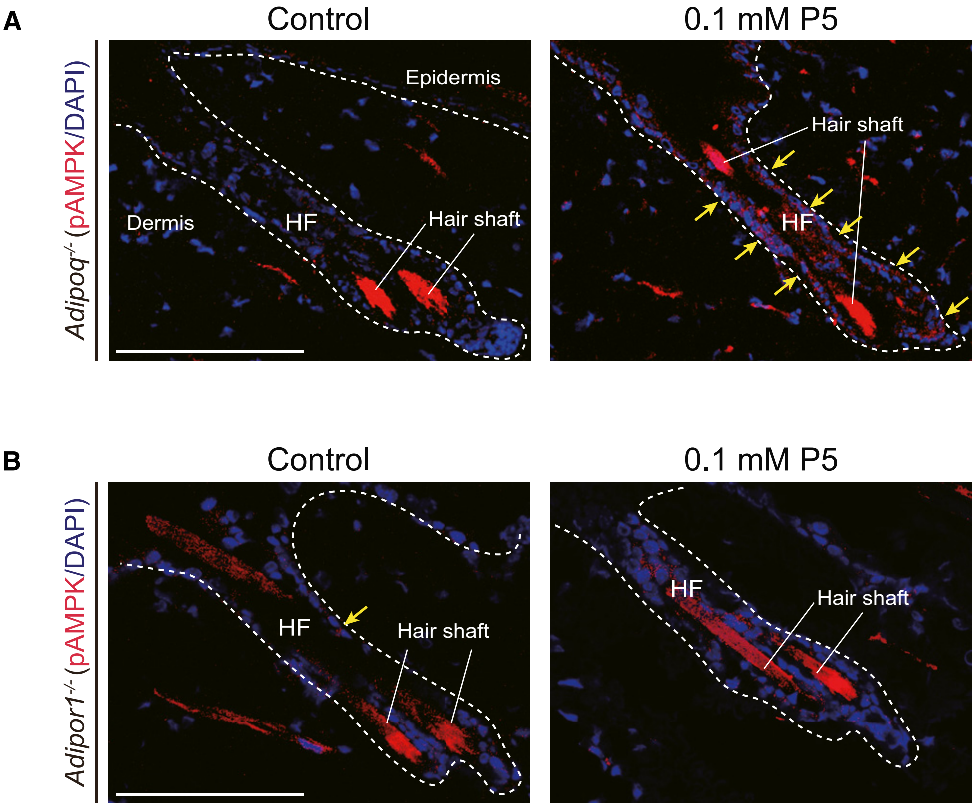

Figure EV3. The upregulation of p-AMPK in HF cells *in vivo* by topical P5 treatment.

A, B p-AMPK in the HF cells of (A) *Adipoq*$^{-/-}$ mice, and (B) *Adipor1*$^{-/-}$ mice after P5 treatment. Yellow arrow indicates p-AMPK signal; scale bar: 100 μm.

© 2021 The Authors

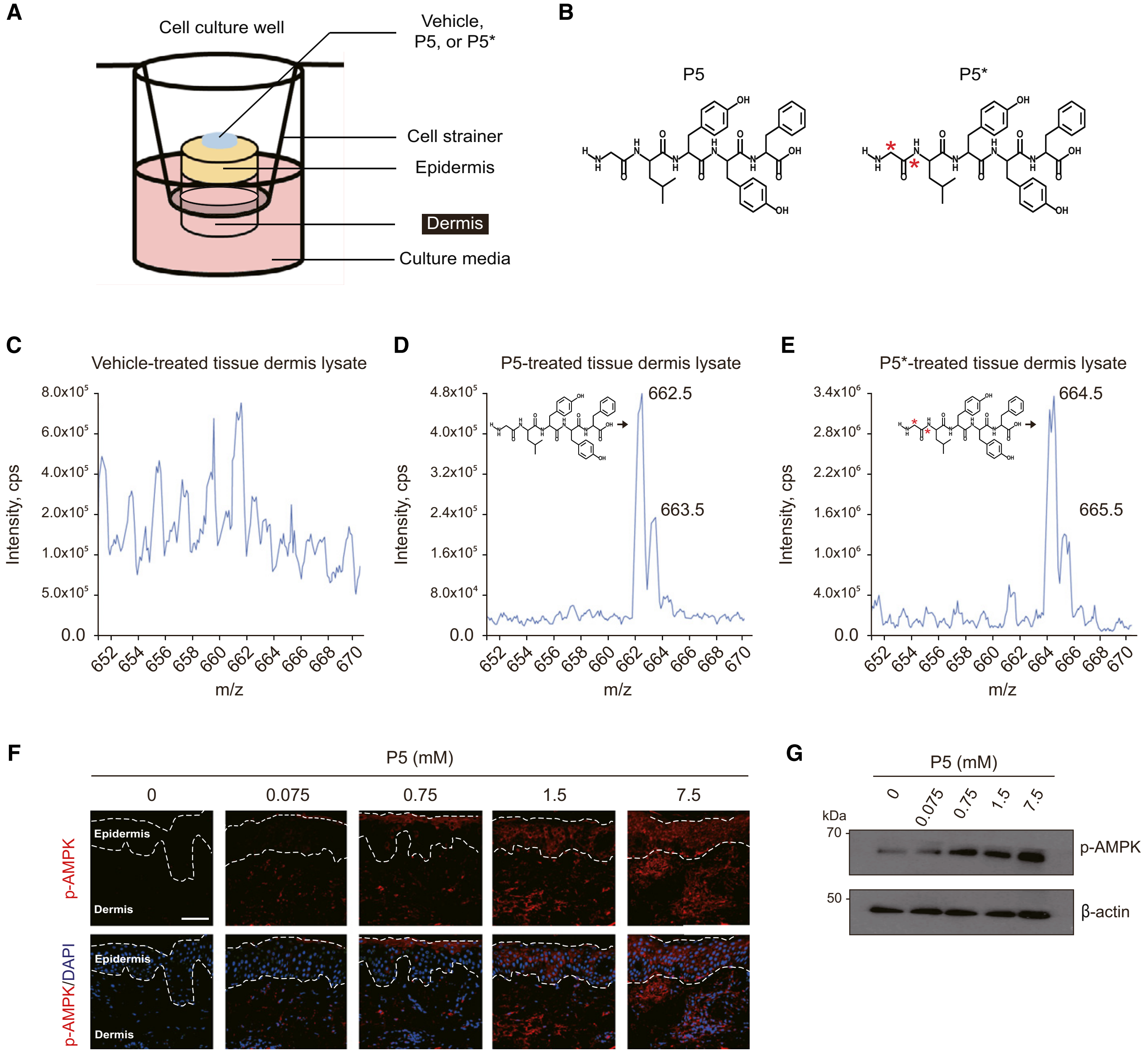

Figure EV4. Transdermal delivery of P5 in *ex vivo* and *in vivo* human skin.

A A schematic illustration of *ex vivo* human skin tissue culture.

B The molecular structure of P5 and isotope-labeled P5 (P5*); red asterisk indicates isotope carbon (^{13}C) atoms.

C–E The qualitative MS data of the dermal lysate from the vehicle-, P5-, or P5*-treated skin tissue, respectively; *m/z*, mass-to-charge ratio.

F IF for p-AMPK in the P5-treated human buttock skin tissue *in vivo*; scale bars, 50 μm.

G Western blot for p-AMPK in the P5-treated human buttock skin tissue lysate ($n = 3$ biological replicates).

Source data are available online for this figure.

© 2021 The Authors

Figure EV5. Analysis of the specific binding pocket of AdipoR1 for P5.

A Sequence logo landscape of MSA of extracellular loop 2 of AdipoR1. The residue numbers are based on the human AdipoR1 amino acid sequence. In a given residue, the height of each letter (measured in bits) is proportionally scaled to the amount of information contributed based on Shannon entropy. The background color of each residue is determined by the degree of conservation in physicochemical properties (cyan to magenta). The investigated residues in the text are marked with red tags (Tyr225, Tyr226, and Tyr229).

B Cartoon diagram of the superposition of APN with the docking model of the AdipoR1-P5 complex. The red circle indicates the position of P5. AdipoR1 (PDB ID: 3WXV), APN (PDB ID: 4DOU), and P5 are colored green, wheat, and magenta, respectively.

C Pulldown of AdipoR1 mutants (residues 89–375) with the GST-fused P5.

Source data are available online for this figure.

© 2021 The Authors

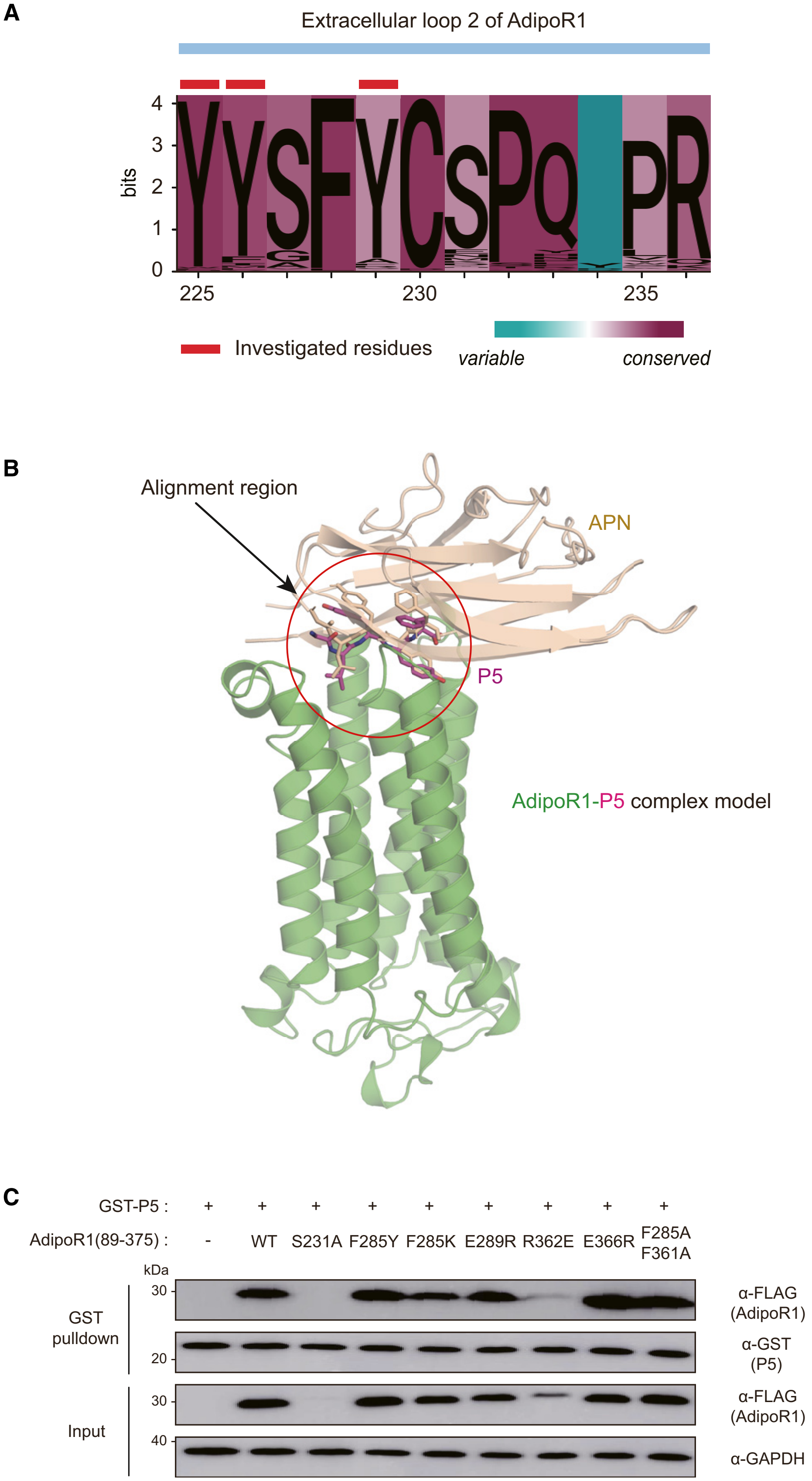
A
Extracellular loop 2 of AdipoR1
bits
4
3
2
1
0
225
230
235
Investigated residues
variable
conserved
B
Alignment region
APN
P5
AdipoR1-P5 complex model
C
GST-P5 :
+
+
+
+
+
+
+
+
+
AdipoR1(89-375) :
-
WT
S231A
F285Y
F285K
E289R
R362E
E366R
F285A F361A
kDa
30
20
30
40
GST pulldown
Input
α-FLAG (AdipoR1)
α-GST (P5)
α-FLAG (AdipoR1)
α-GAPDH

Figure EV5.
