# Supplementary figures and images for: Discovery of a transdermally deliverable pentapeptide for activating AdipoR1 to promote hair growth
Source: EMBO Mol Med. 2021 Sep 6;13(10):e13790. doi: 10.15252/emmm.202013790 (PMC8495455; doi:10.15252/emmm.202013790)

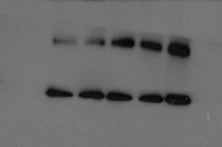

Supplement: Supplementary file 3 — Source Data for Expanded View/Appendix [file EMMM-13-e13790-s002.zip › Source Data for Expanded View and Appendix figures/Fig EV4/FIgEV4G.tif]

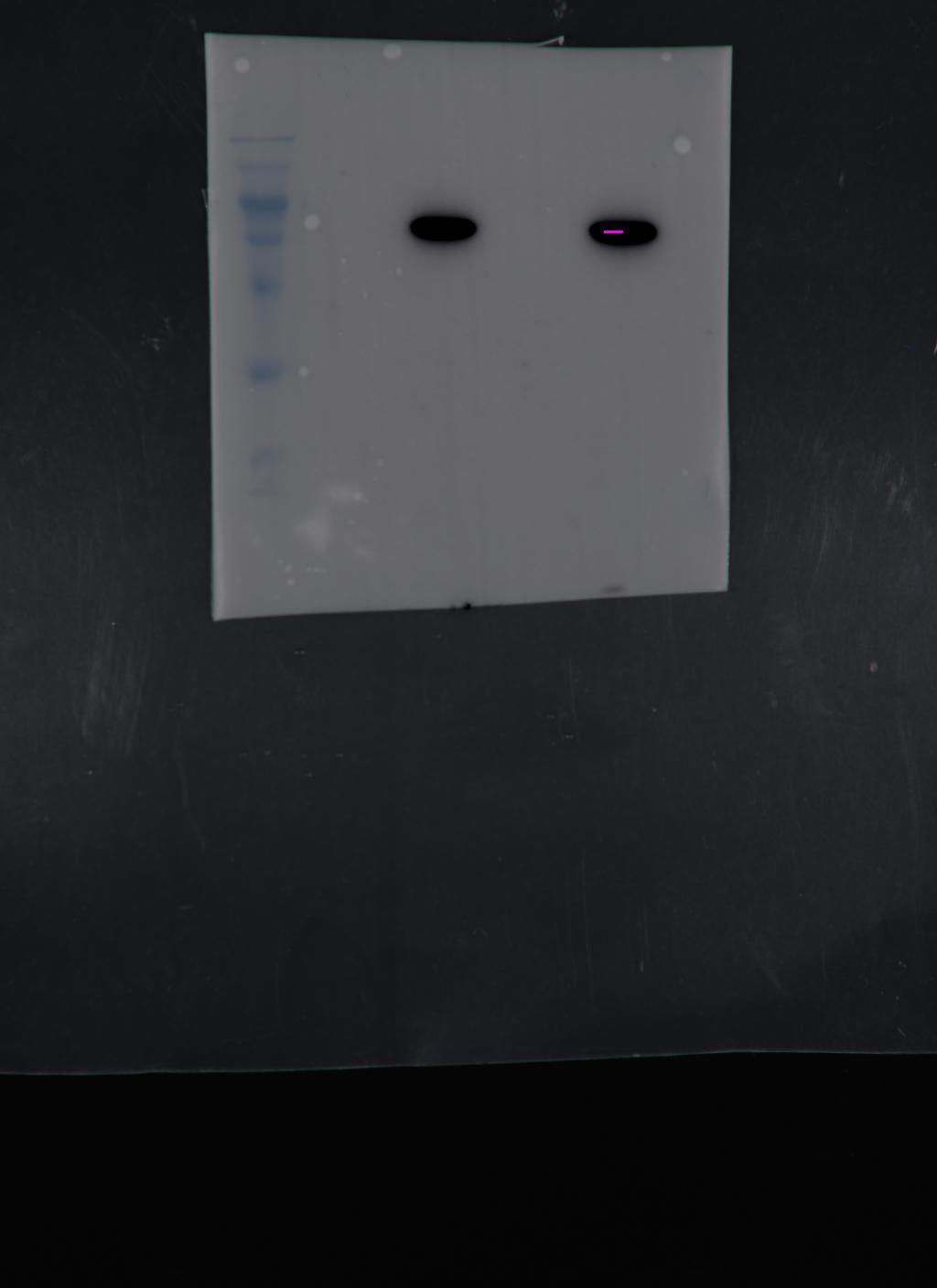

Supplement: Supplementary file 4 — Source Data for Figure 1 [file EMMM-13-e13790-s003.zip › EMM-2020-13790_SourceDataForFigure1/Figure1C/AdipoR1(1-375)_Input_Flag.jpg]

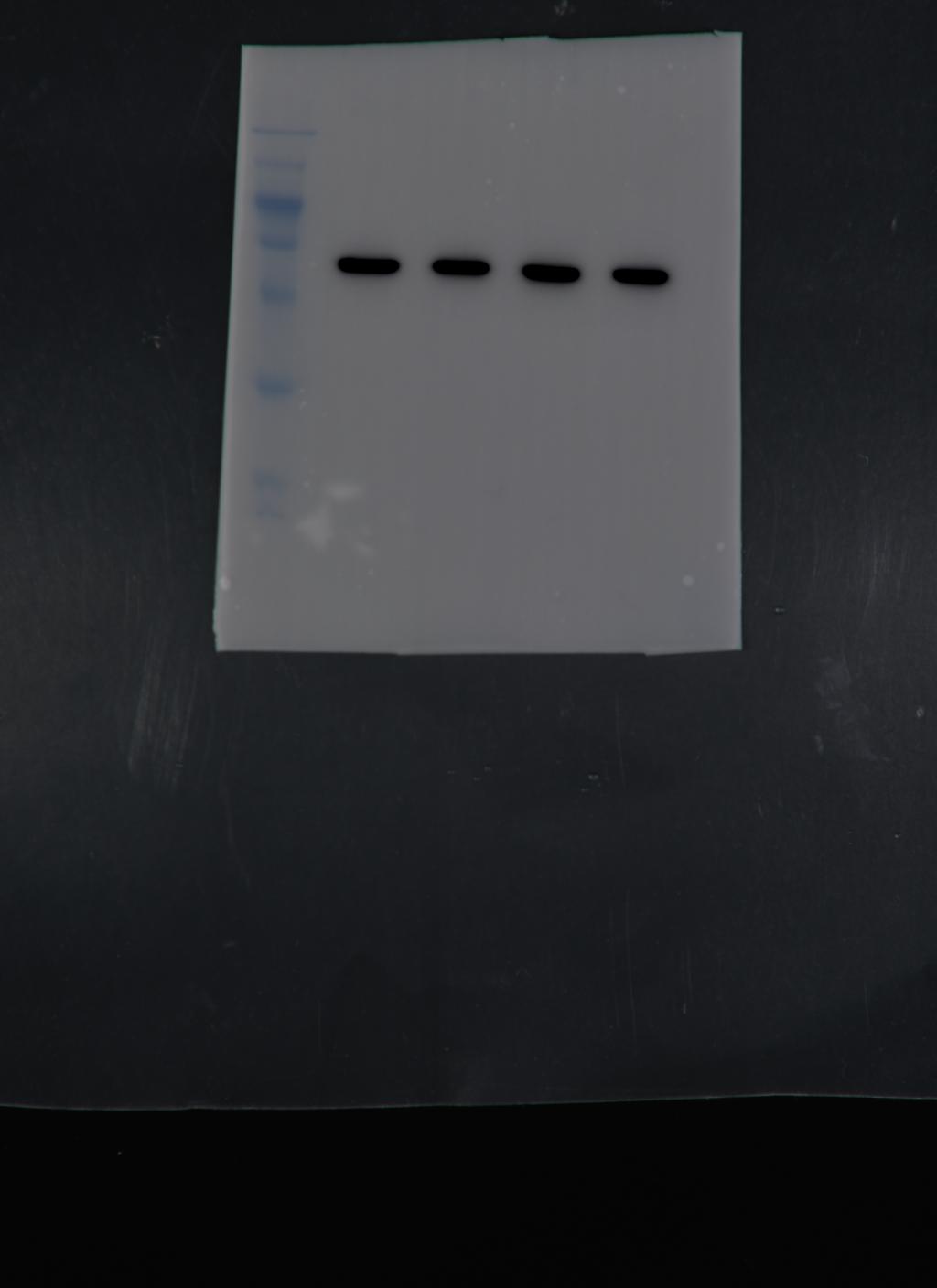

Supplement: Supplementary file 4 — Source Data for Figure 1 [file EMMM-13-e13790-s003.zip › EMM-2020-13790_SourceDataForFigure1/Figure1C/AdipoR1(1-375)_Input_GAPDH.jpg]

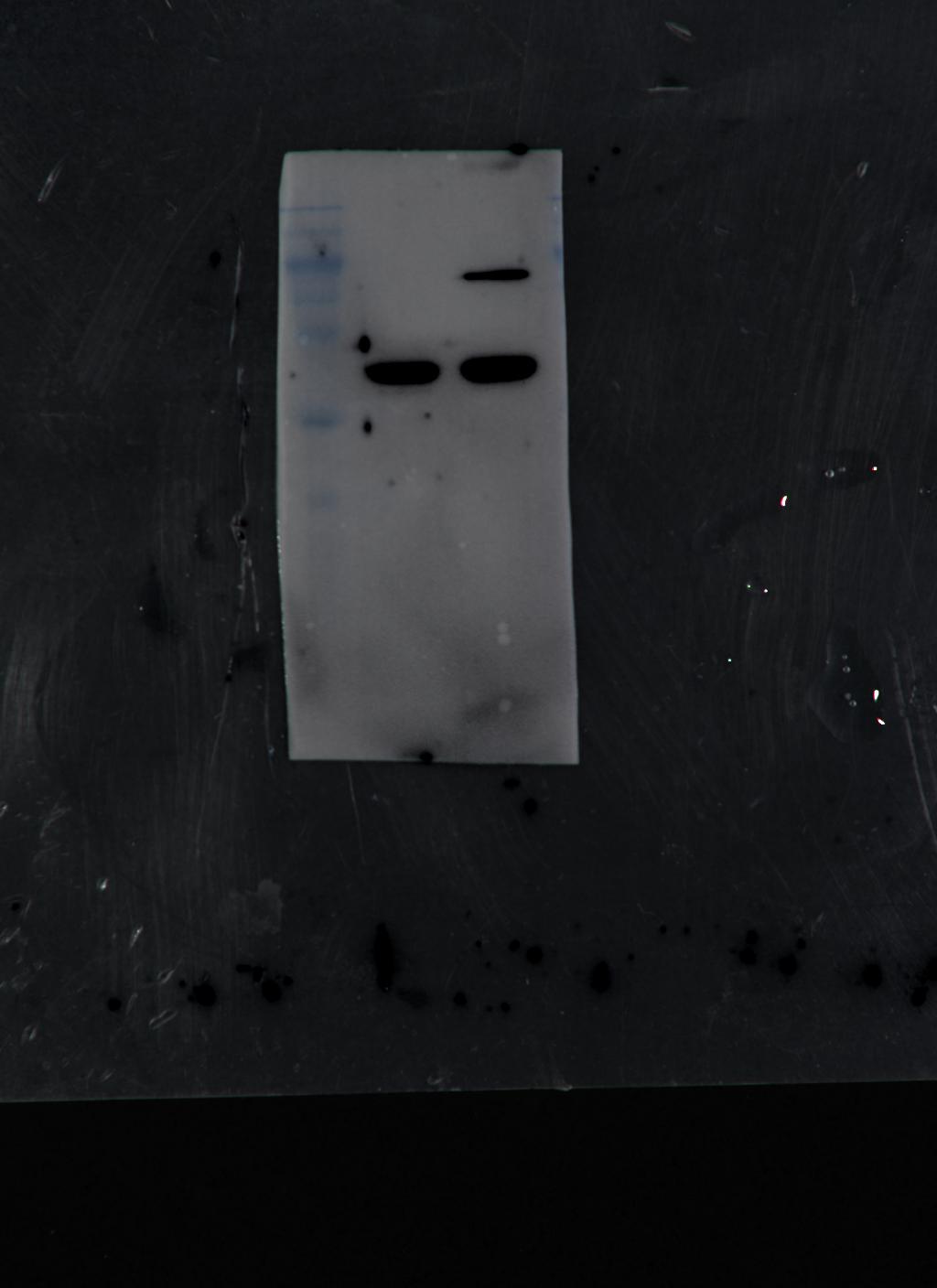

Supplement: Supplementary file 4 — Source Data for Figure 1 [file EMMM-13-e13790-s003.zip › EMM-2020-13790_SourceDataForFigure1/Figure1C/AdipoR1(1-375)_PD_Flag1.jpg]

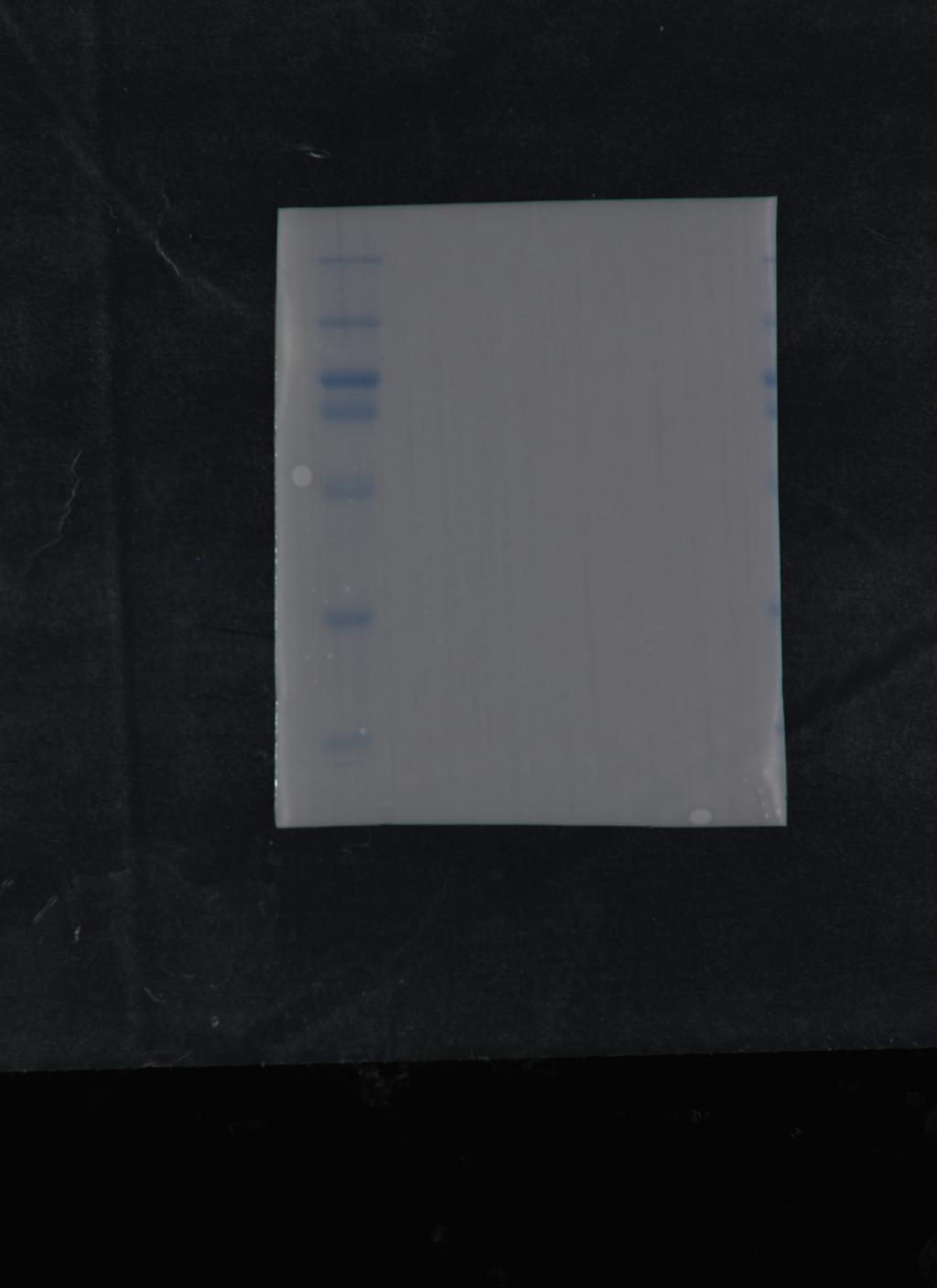

Supplement: Supplementary file 4 — Source Data for Figure 1 [file EMMM-13-e13790-s003.zip › EMM-2020-13790_SourceDataForFigure1/Figure1C/AdipoR1(1-375)_PD_flag2.jpg]

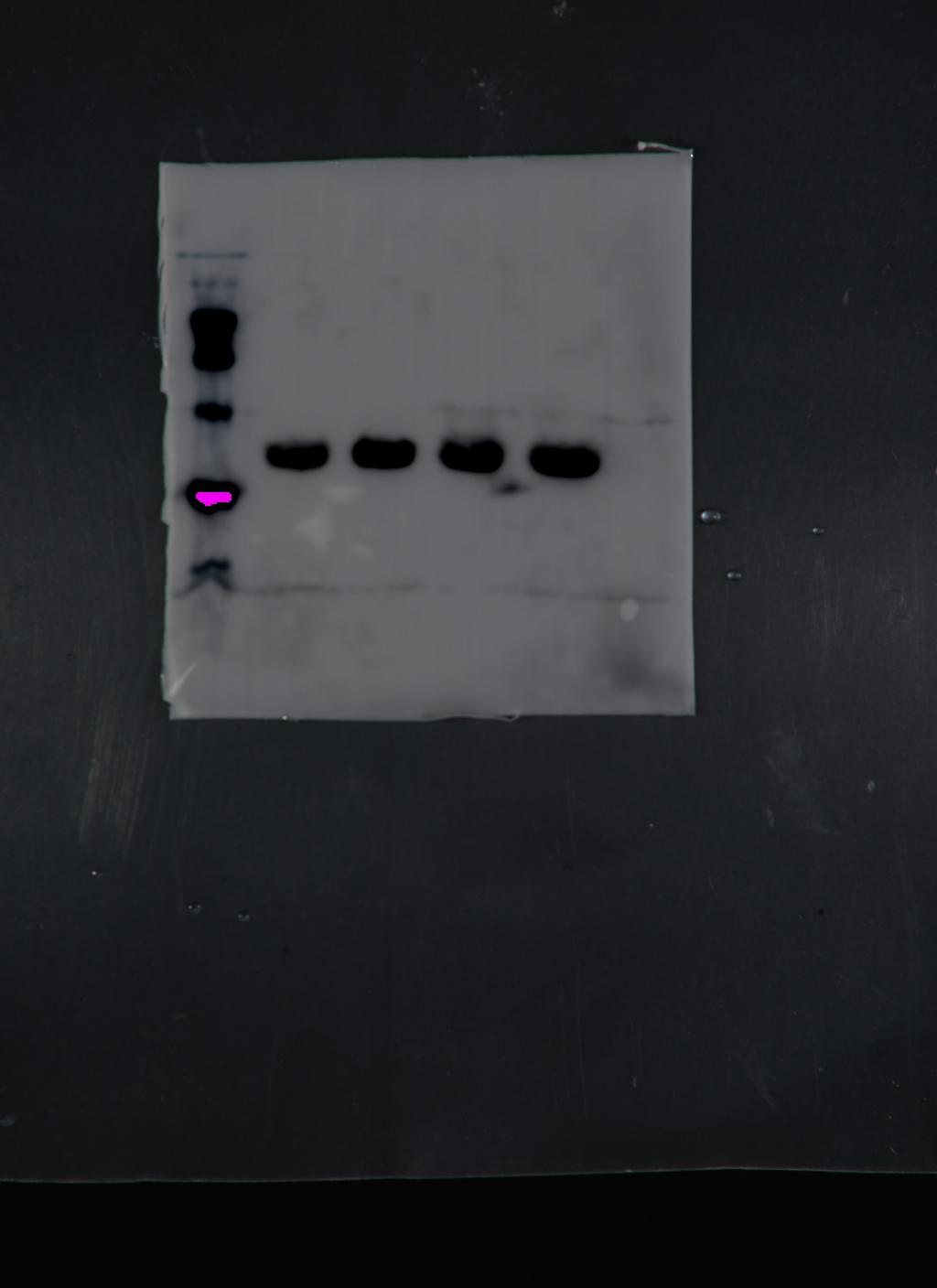

Supplement: Supplementary file 4 — Source Data for Figure 1 [file EMMM-13-e13790-s003.zip › EMM-2020-13790_SourceDataForFigure1/Figure1C/AdipoR1(1-375)_PD_GST.jpg]

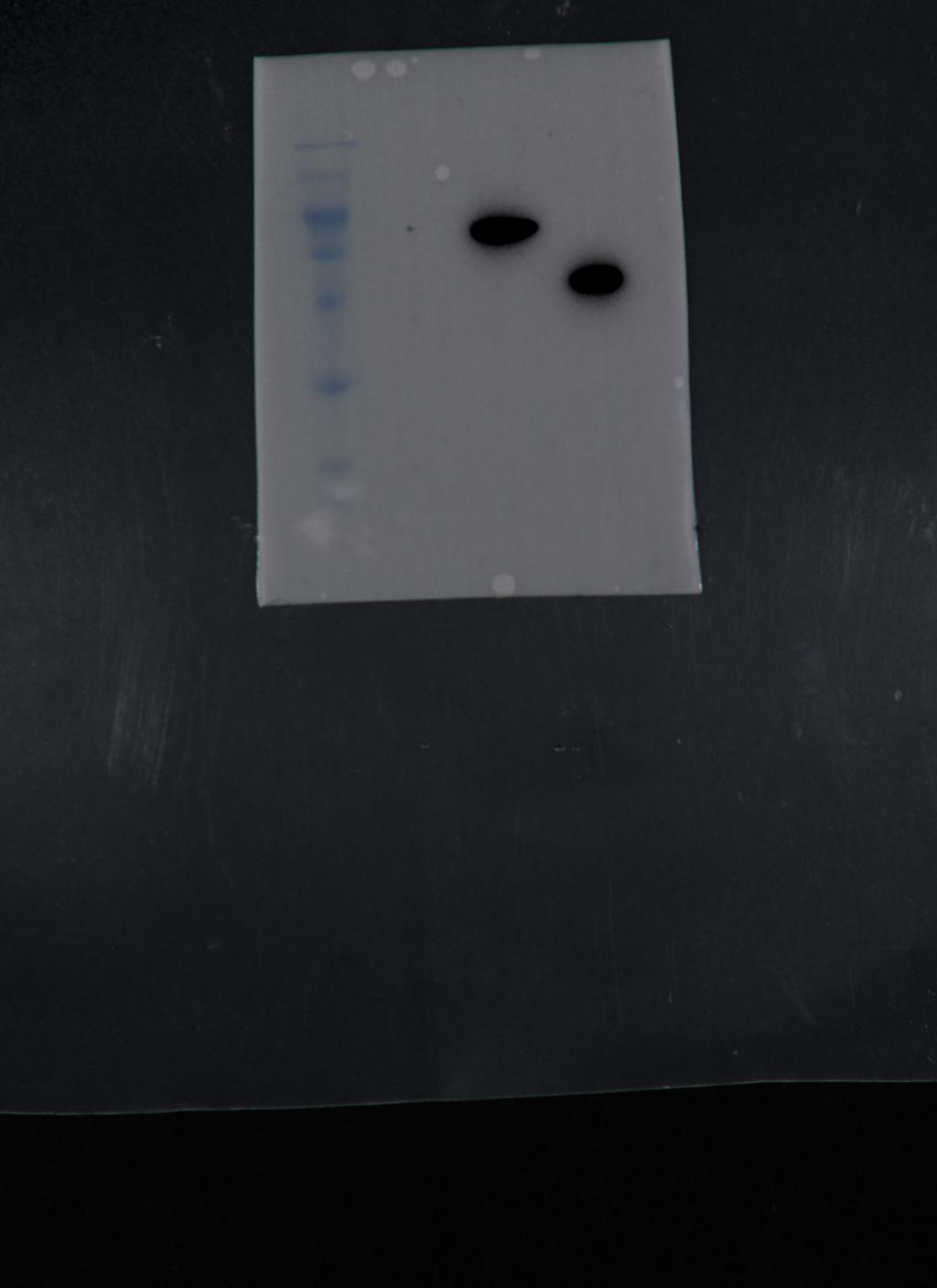

Supplement: Supplementary file 4 — Source Data for Figure 1 [file EMMM-13-e13790-s003.zip › EMM-2020-13790_SourceDataForFigure1/Figure1D/AdipoR1(1-375,89-375)_Input_Flag.jpg]

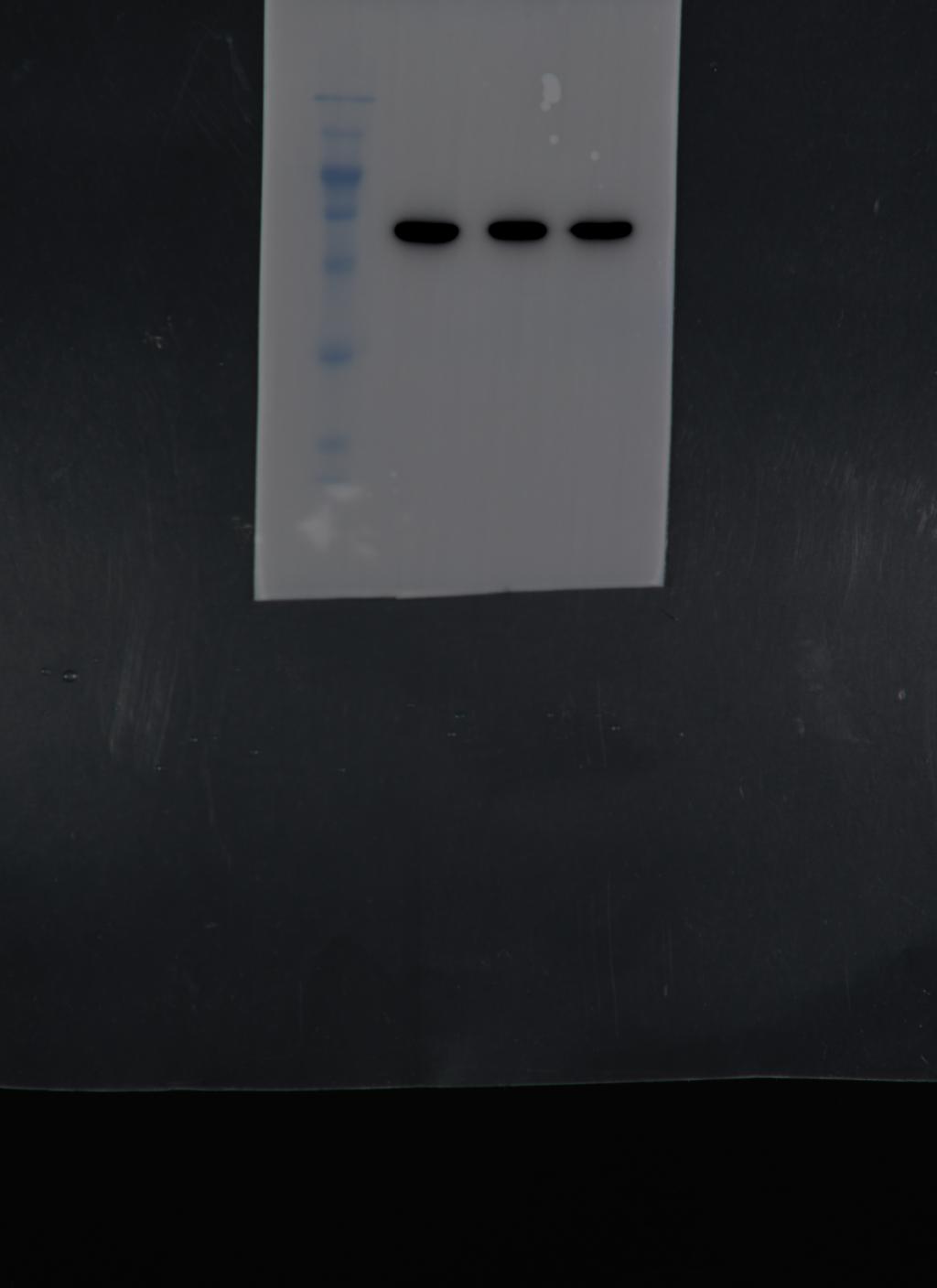

Supplement: Supplementary file 4 — Source Data for Figure 1 [file EMMM-13-e13790-s003.zip › EMM-2020-13790_SourceDataForFigure1/Figure1D/AdipoR1(1-375,89-375)_Input_GAPDH.jpg]

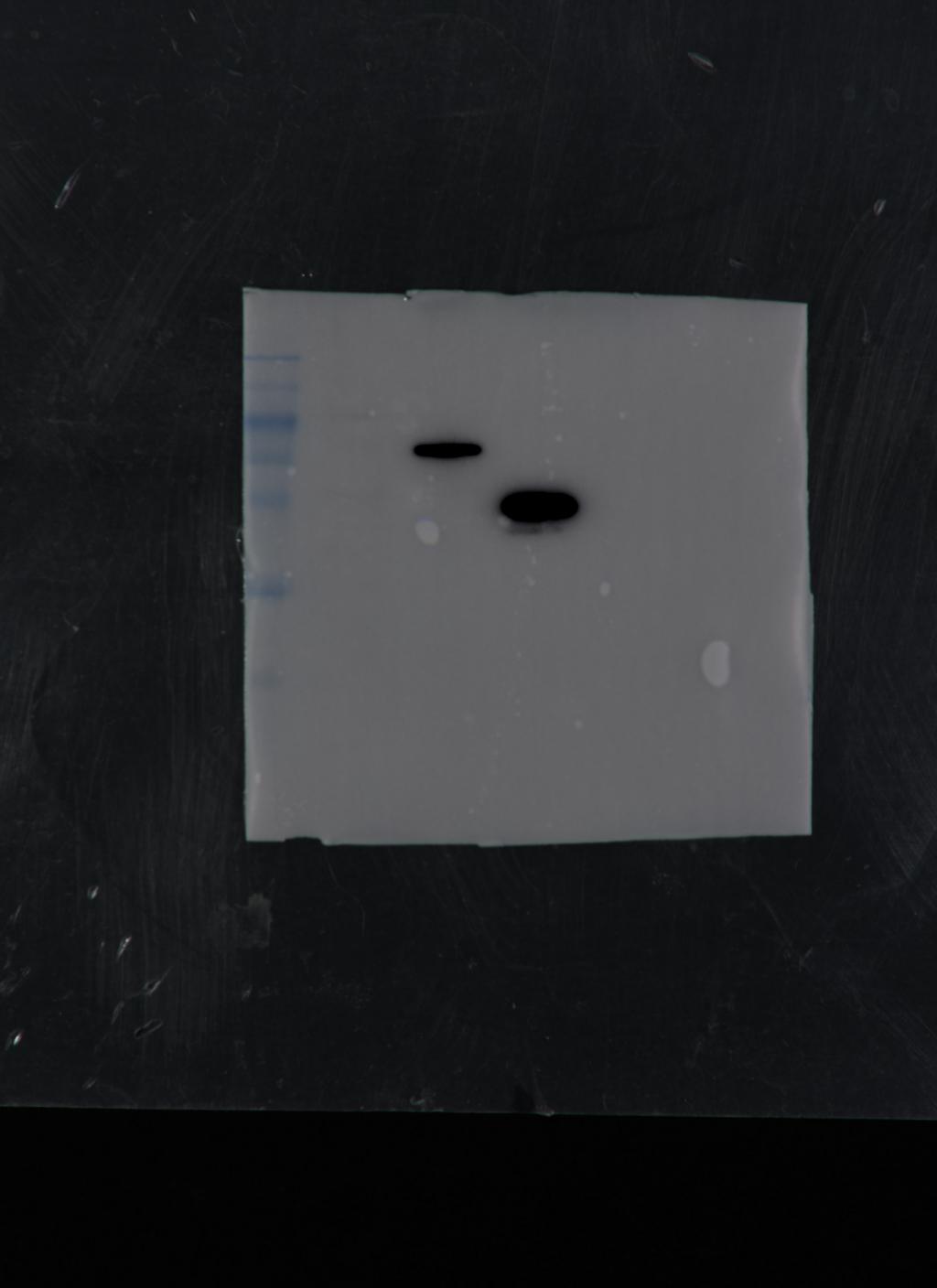

Supplement: Supplementary file 4 — Source Data for Figure 1 [file EMMM-13-e13790-s003.zip › EMM-2020-13790_SourceDataForFigure1/Figure1D/AdipoR1(1-375,89-375)_PD_Flag.jpg]

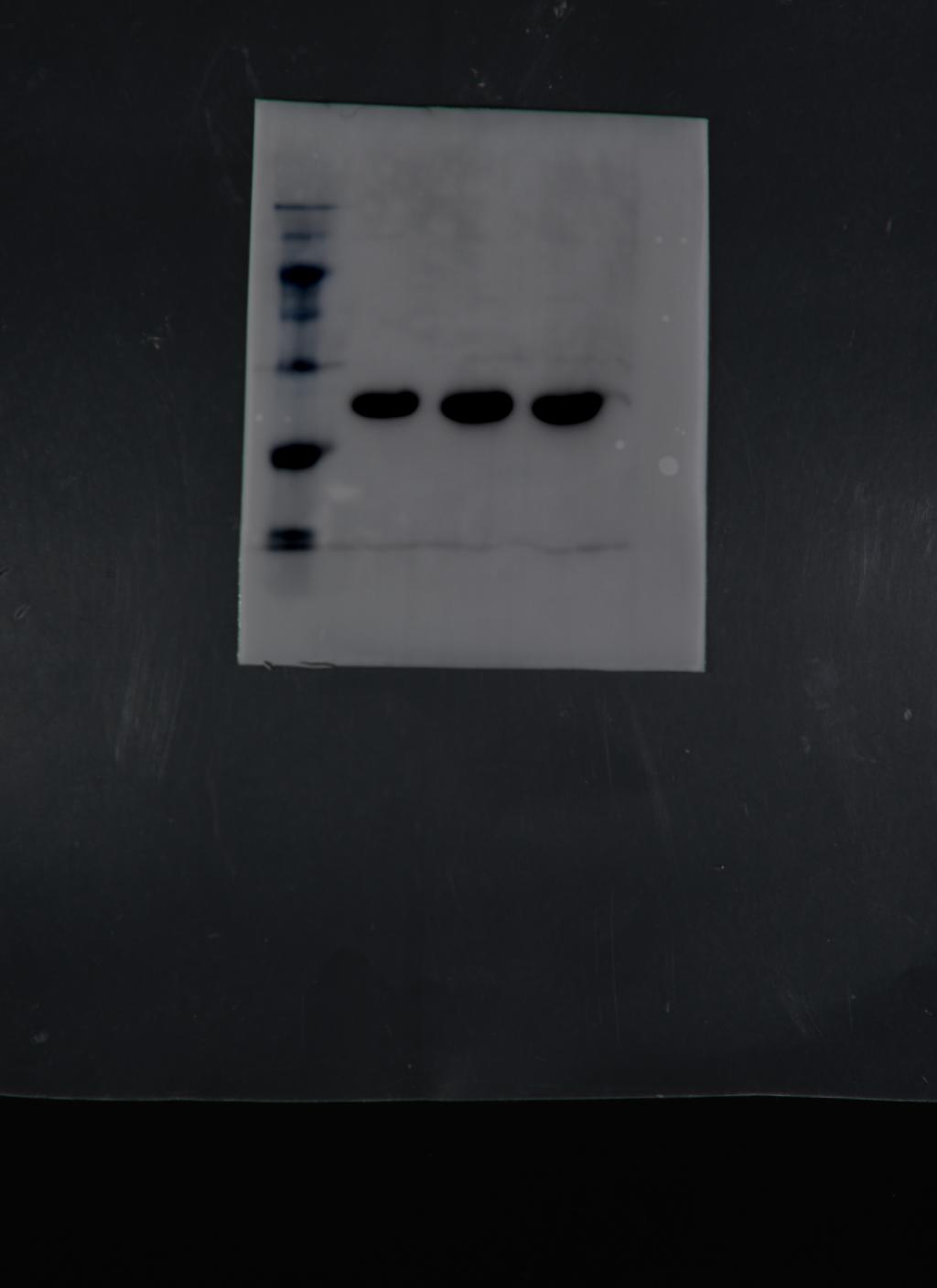

Supplement: Supplementary file 4 — Source Data for Figure 1 [file EMMM-13-e13790-s003.zip › EMM-2020-13790_SourceDataForFigure1/Figure1D/AdipoR1(1-375,89-375)_PD_GST.jpg]

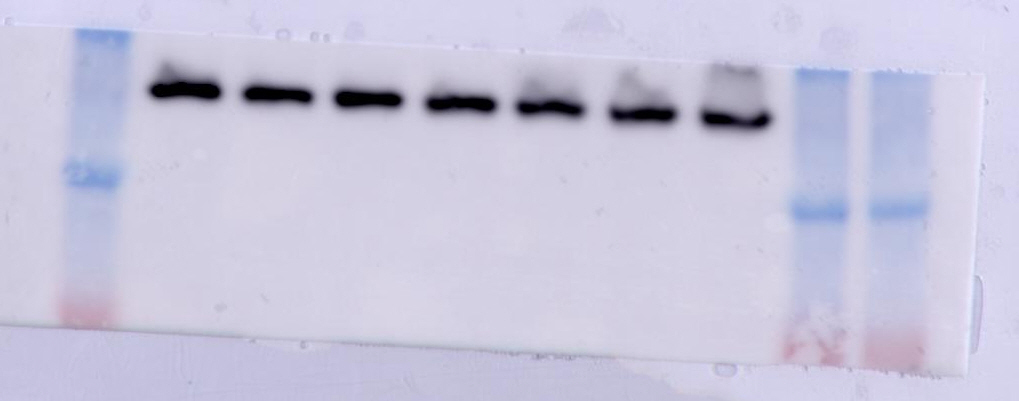

Supplement: Supplementary file 5 — Source Data for Figure 2 [file EMMM-13-e13790-s005.zip › EMM-2020-13790_SourceDataForFigure2/Fig 2A/Fig2A_DP_b-actin.jpg]

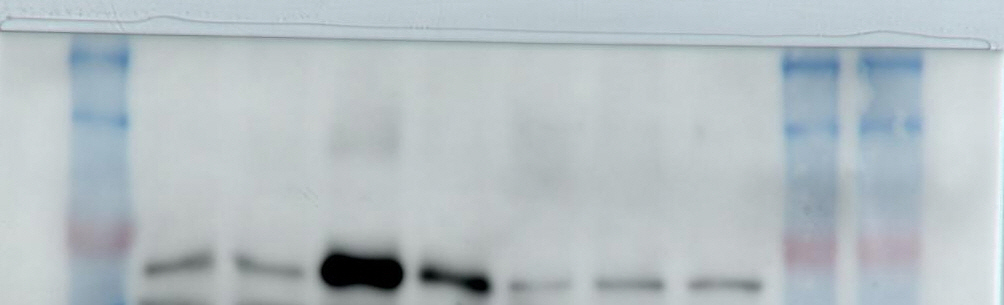

Supplement: Supplementary file 5 — Source Data for Figure 2 [file EMMM-13-e13790-s005.zip › EMM-2020-13790_SourceDataForFigure2/Fig 2A/Fig2A_DP_p-AMPK.jpg]

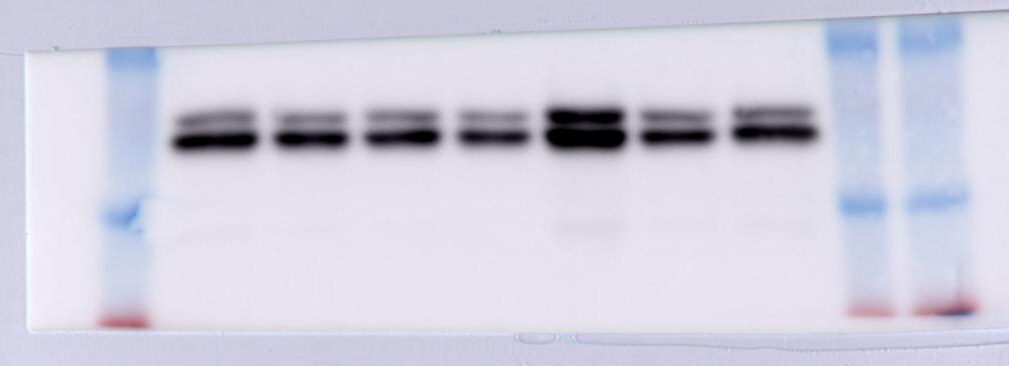

Supplement: Supplementary file 5 — Source Data for Figure 2 [file EMMM-13-e13790-s005.zip › EMM-2020-13790_SourceDataForFigure2/Fig 2A/Fig2A_DP_p-ERK12.jpg]

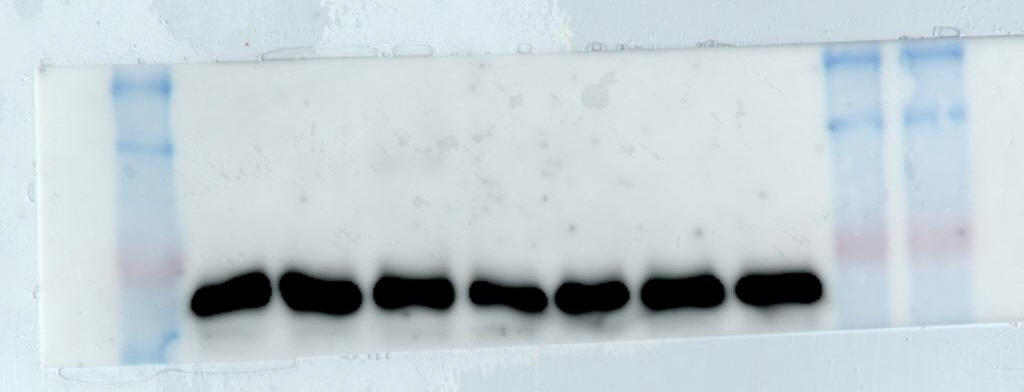

Supplement: Supplementary file 5 — Source Data for Figure 2 [file EMMM-13-e13790-s005.zip › EMM-2020-13790_SourceDataForFigure2/Fig 2A/Fig2A_DP_t-AMPK.jpg]

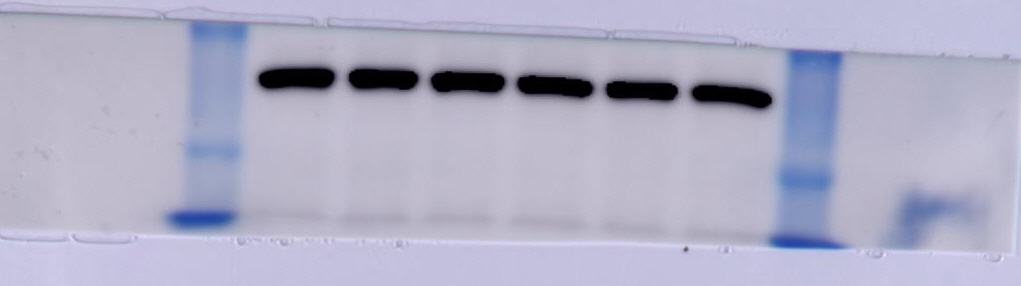

Supplement: Supplementary file 5 — Source Data for Figure 2 [file EMMM-13-e13790-s005.zip › EMM-2020-13790_SourceDataForFigure2/Fig 2A/Fig2A_ORS_b-actin.jpg]

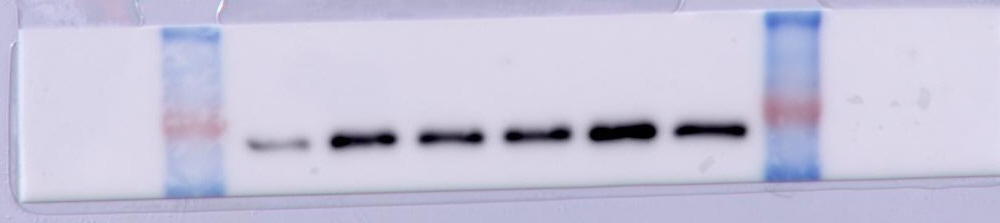

Supplement: Supplementary file 5 — Source Data for Figure 2 [file EMMM-13-e13790-s005.zip › EMM-2020-13790_SourceDataForFigure2/Fig 2A/Fig2A_ORS_p-AMPK.jpg]

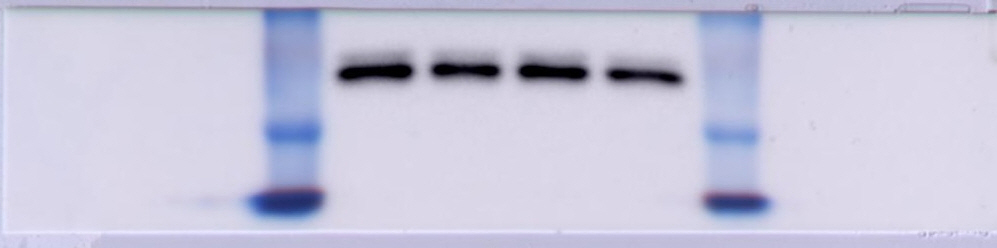

Supplement: Supplementary file 5 — Source Data for Figure 2 [file EMMM-13-e13790-s005.zip › EMM-2020-13790_SourceDataForFigure2/Fig 2A/Fig2A_ORS_p-ERK12.jpg]

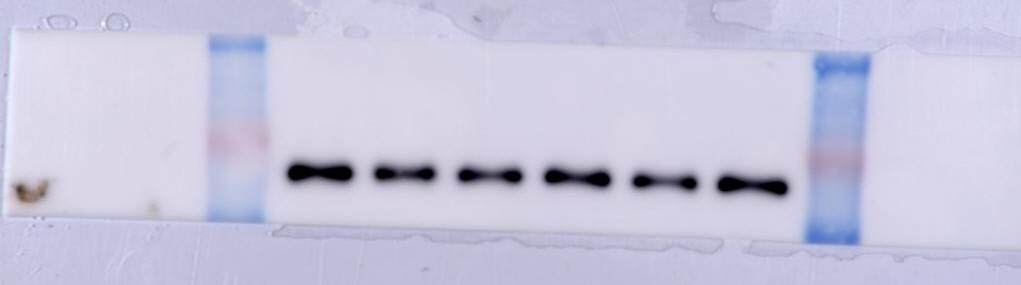

Supplement: Supplementary file 5 — Source Data for Figure 2 [file EMMM-13-e13790-s005.zip › EMM-2020-13790_SourceDataForFigure2/Fig 2A/Fig2A_ORS_t-AMPK.jpg]

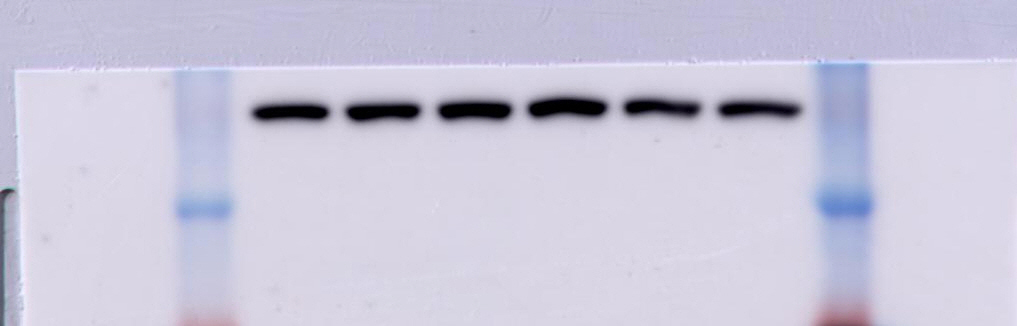

Supplement: Supplementary file 5 — Source Data for Figure 2 [file EMMM-13-e13790-s005.zip › EMM-2020-13790_SourceDataForFigure2/Fig 2B/Fig2B_DP_b-actin.jpg]

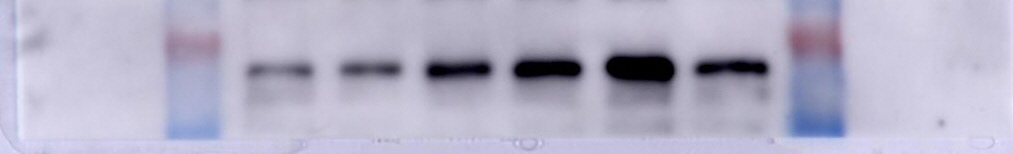

Supplement: Supplementary file 5 — Source Data for Figure 2 [file EMMM-13-e13790-s005.zip › EMM-2020-13790_SourceDataForFigure2/Fig 2B/Fig2B_DP_p-AMPK.jpg]

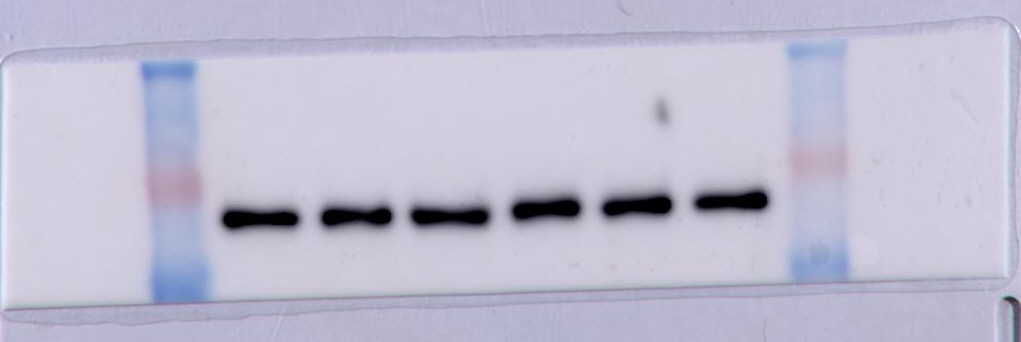

Supplement: Supplementary file 5 — Source Data for Figure 2 [file EMMM-13-e13790-s005.zip › EMM-2020-13790_SourceDataForFigure2/Fig 2B/Fig2B_DP_t-AMPK.jpg]

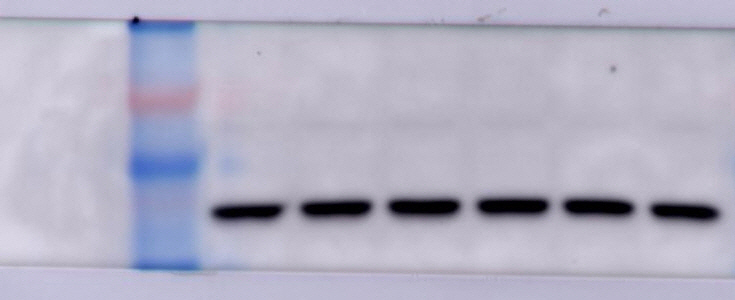

Supplement: Supplementary file 5 — Source Data for Figure 2 [file EMMM-13-e13790-s005.zip › EMM-2020-13790_SourceDataForFigure2/Fig 2B/Fig2B_ORS_b-actin.jpg]

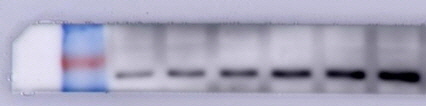

Supplement: Supplementary file 5 — Source Data for Figure 2 [file EMMM-13-e13790-s005.zip › EMM-2020-13790_SourceDataForFigure2/Fig 2B/Fig2B_ORS_p-AMPK.jpg]

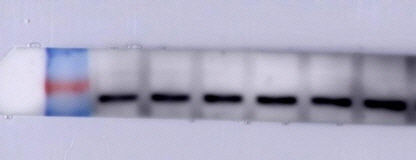

Supplement: Supplementary file 5 — Source Data for Figure 2 [file EMMM-13-e13790-s005.zip › EMM-2020-13790_SourceDataForFigure2/Fig 2B/Fig2B_ORS_t-AMPK_.jpg]

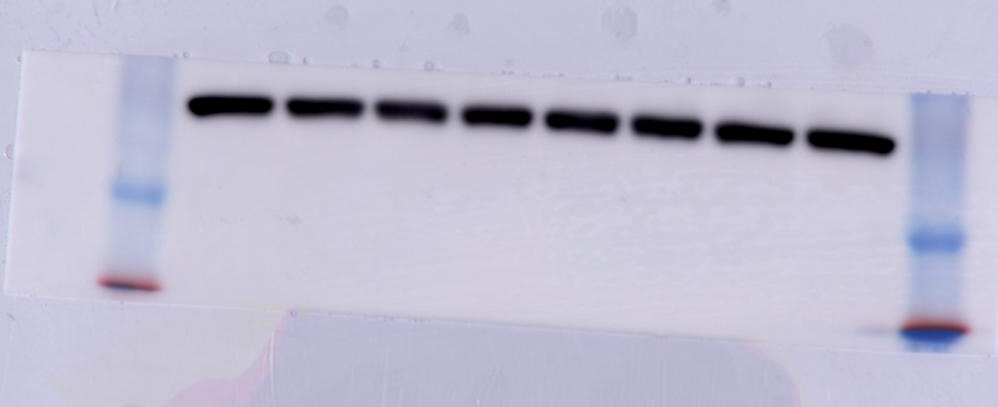

Supplement: Supplementary file 5 — Source Data for Figure 2 [file EMMM-13-e13790-s005.zip › EMM-2020-13790_SourceDataForFigure2/Fig 2C/Fig2C_DP_b-actin.jpg]

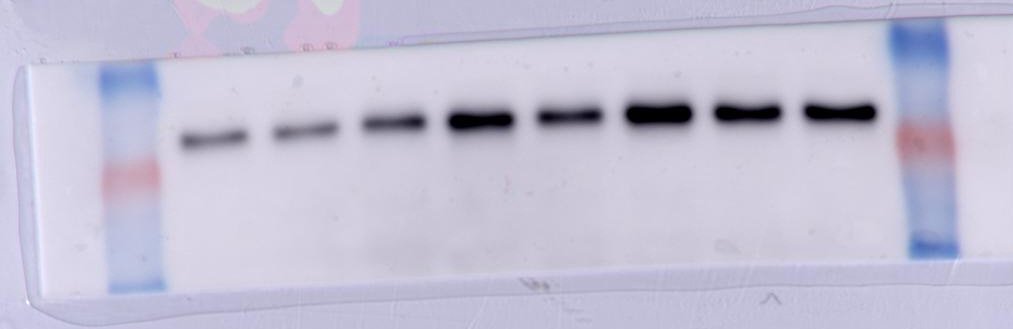

Supplement: Supplementary file 5 — Source Data for Figure 2 [file EMMM-13-e13790-s005.zip › EMM-2020-13790_SourceDataForFigure2/Fig 2C/Fig2C_DP_p-AMPK.jpg]

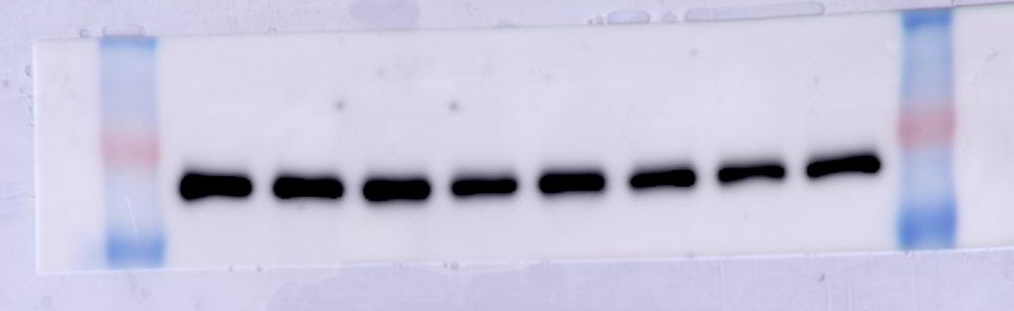

Supplement: Supplementary file 5 — Source Data for Figure 2 [file EMMM-13-e13790-s005.zip › EMM-2020-13790_SourceDataForFigure2/Fig 2C/Fig2C_DP_t-AMPK.jpg]

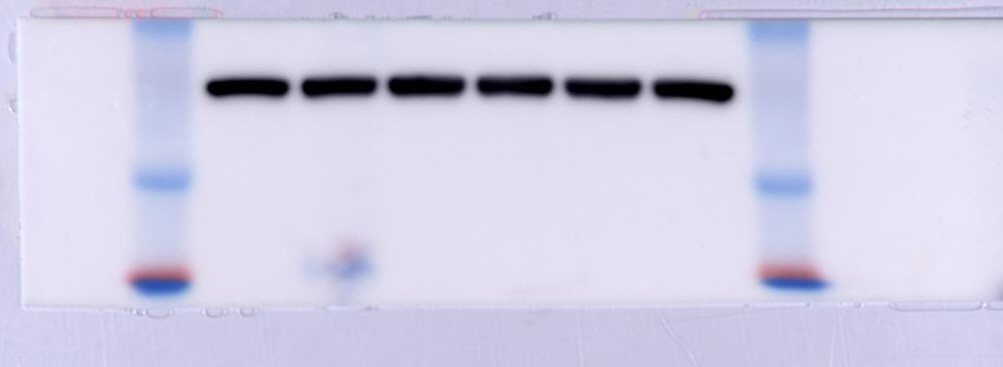

Supplement: Supplementary file 5 — Source Data for Figure 2 [file EMMM-13-e13790-s005.zip › EMM-2020-13790_SourceDataForFigure2/Fig 2C/Fig2C_ORS_b-actin.jpg]

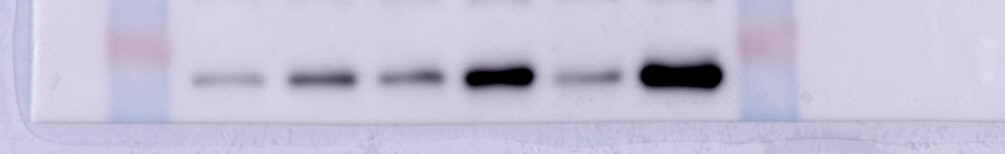

Supplement: Supplementary file 5 — Source Data for Figure 2 [file EMMM-13-e13790-s005.zip › EMM-2020-13790_SourceDataForFigure2/Fig 2C/Fig2C_ORS_p-AMPK.jpg]

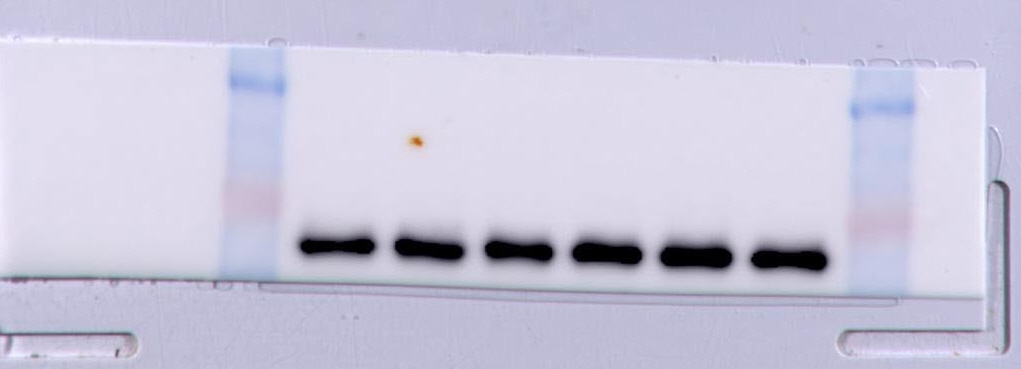

Supplement: Supplementary file 5 — Source Data for Figure 2 [file EMMM-13-e13790-s005.zip › EMM-2020-13790_SourceDataForFigure2/Fig 2C/Fig2C_ORS_t-AMPK.jpg]

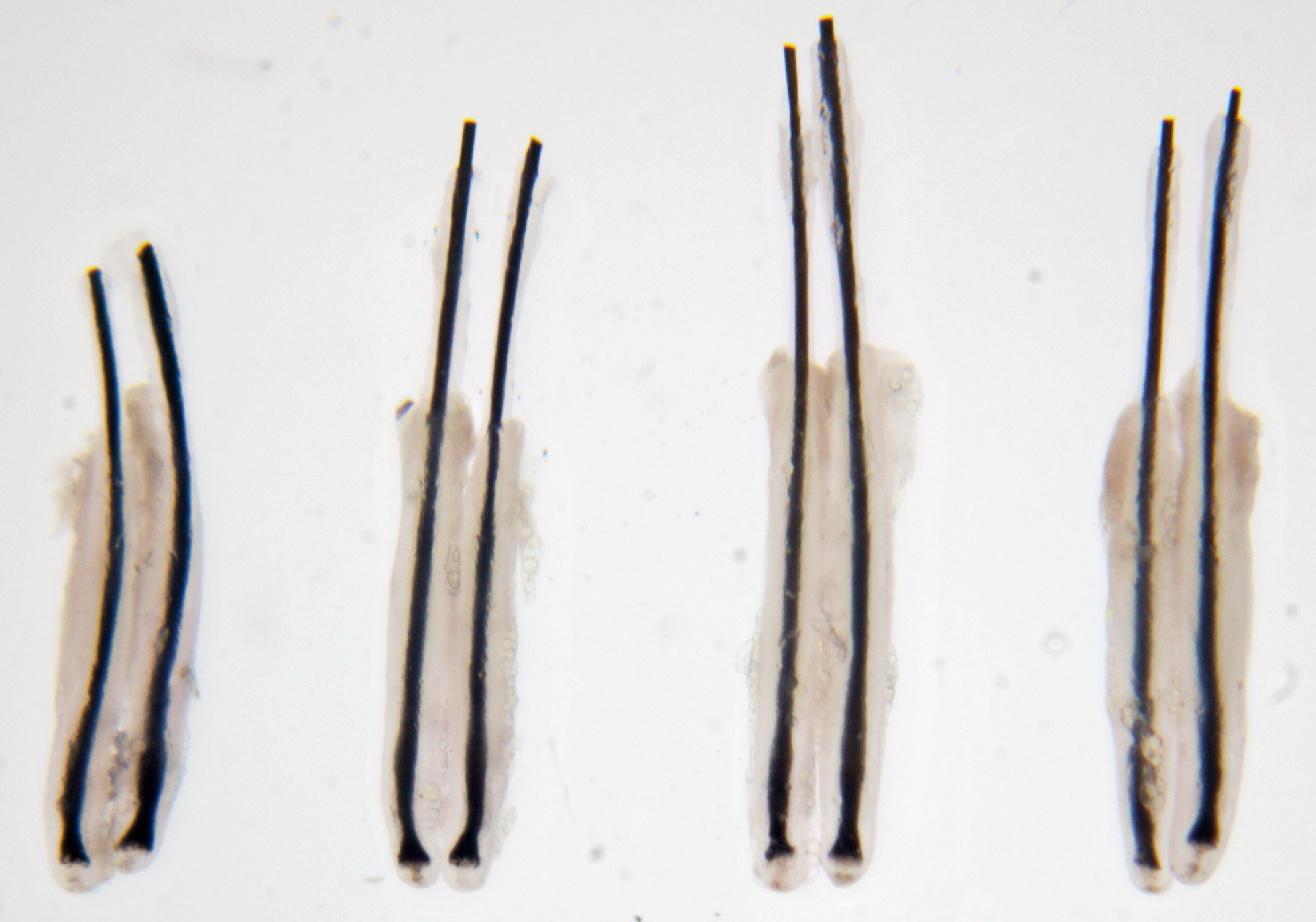

Supplement: Supplementary file 6 — Source Data for Figure 3 [file EMMM-13-e13790-s001.zip › EMM-2020-13790_SourceDataForFigure3/Fig 3A.jpg]

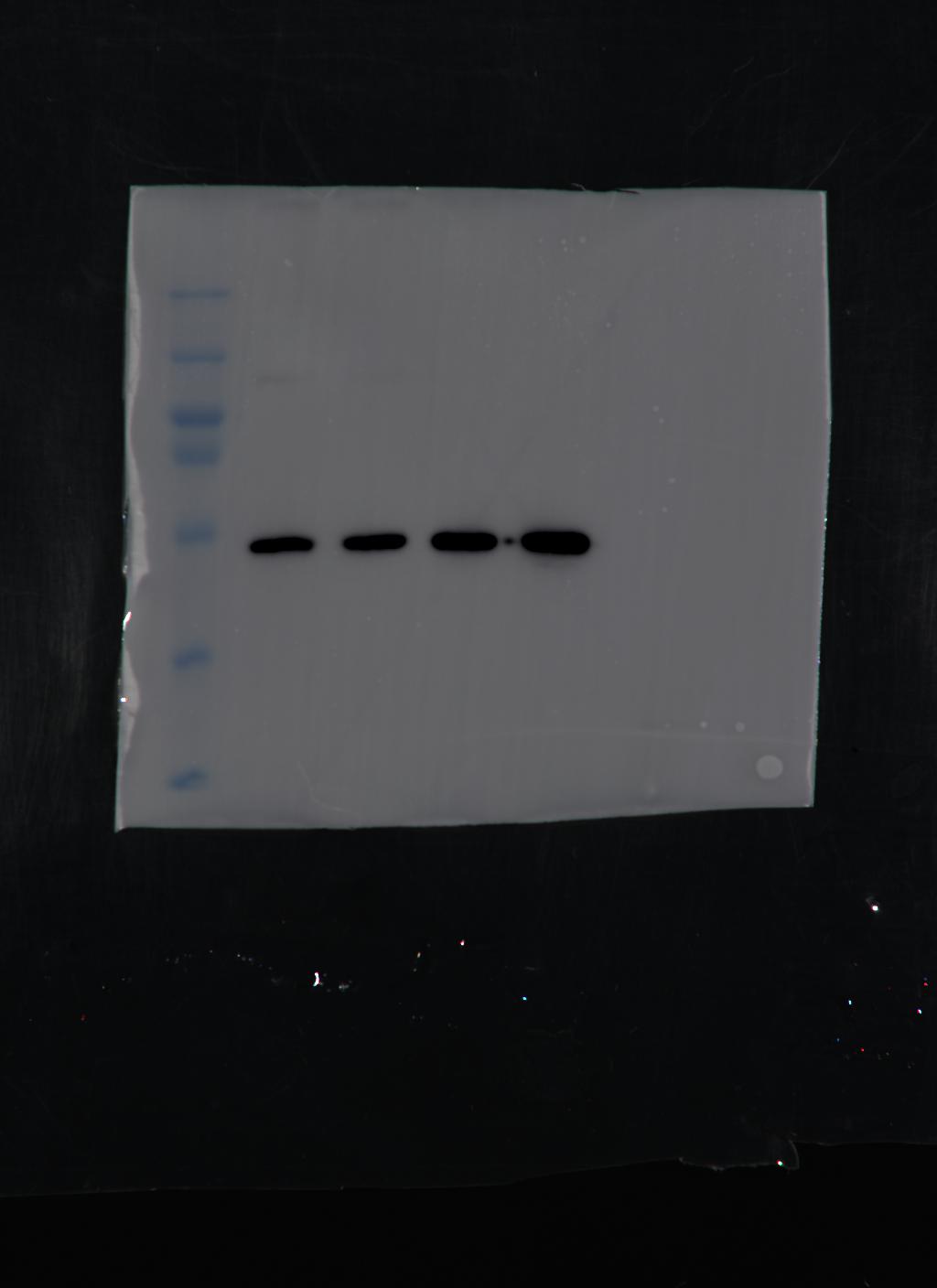

Supplement: Supplementary file 8 — Source Data for Figure 5 [file EMMM-13-e13790-s008.zip › EMM-2020-13790_SourceDataForFigure5/Fig 5C/Y_input_Flag.jpg]

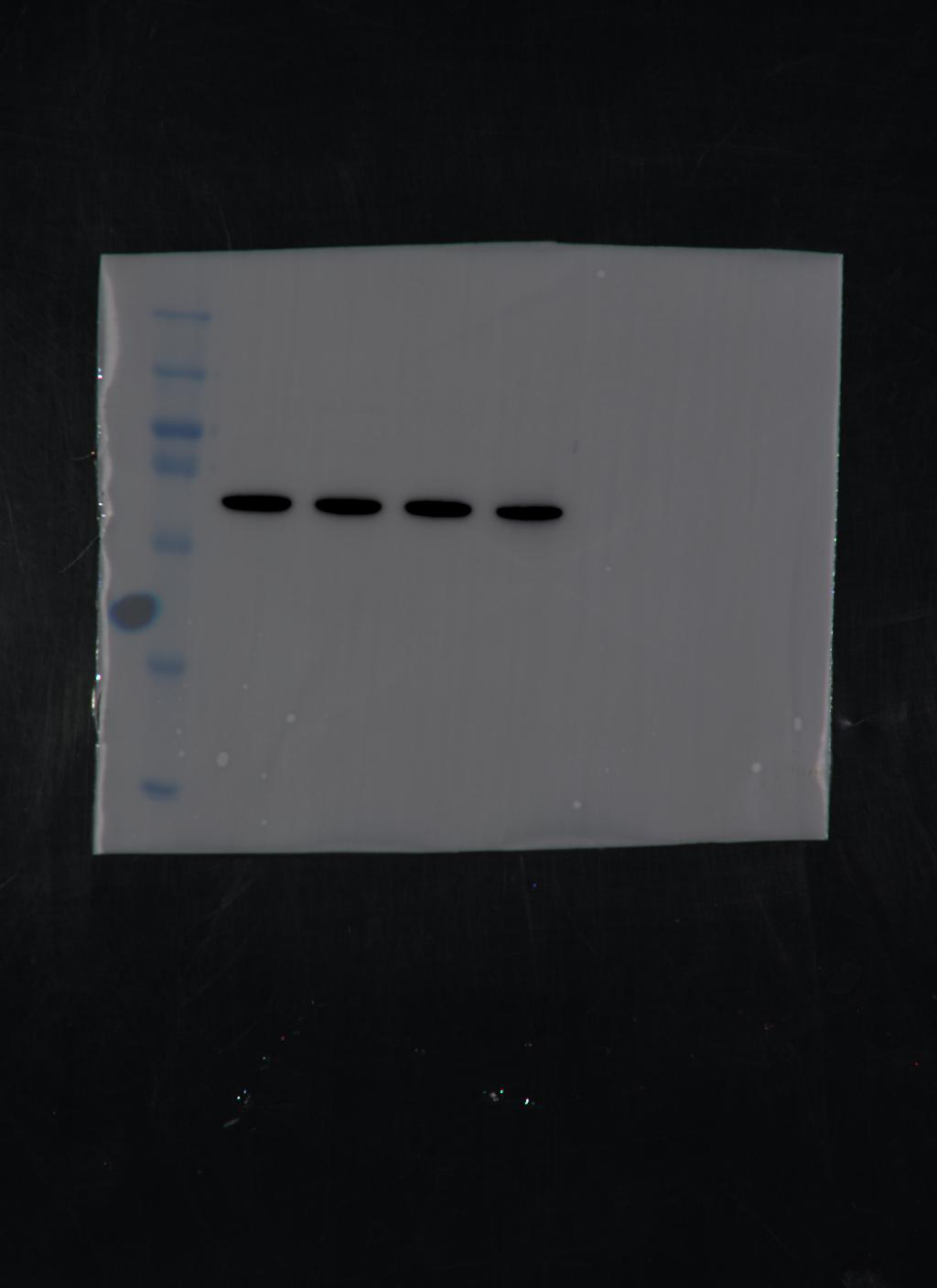

Supplement: Supplementary file 8 — Source Data for Figure 5 [file EMMM-13-e13790-s008.zip › EMM-2020-13790_SourceDataForFigure5/Fig 5C/Y_input_GAPDH.jpg]

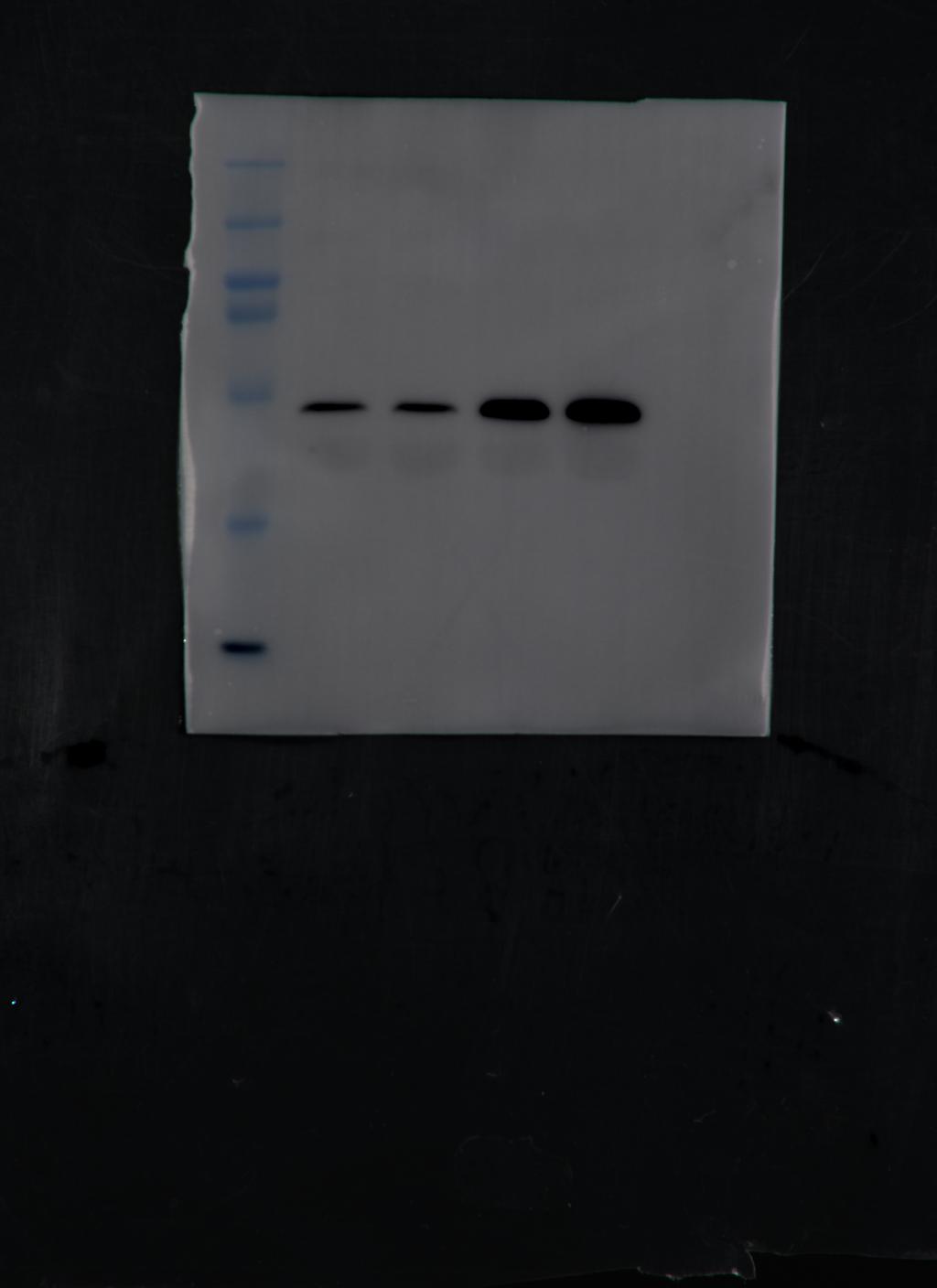

Supplement: Supplementary file 8 — Source Data for Figure 5 [file EMMM-13-e13790-s008.zip › EMM-2020-13790_SourceDataForFigure5/Fig 5C/Y_PD_Flag.jpg]

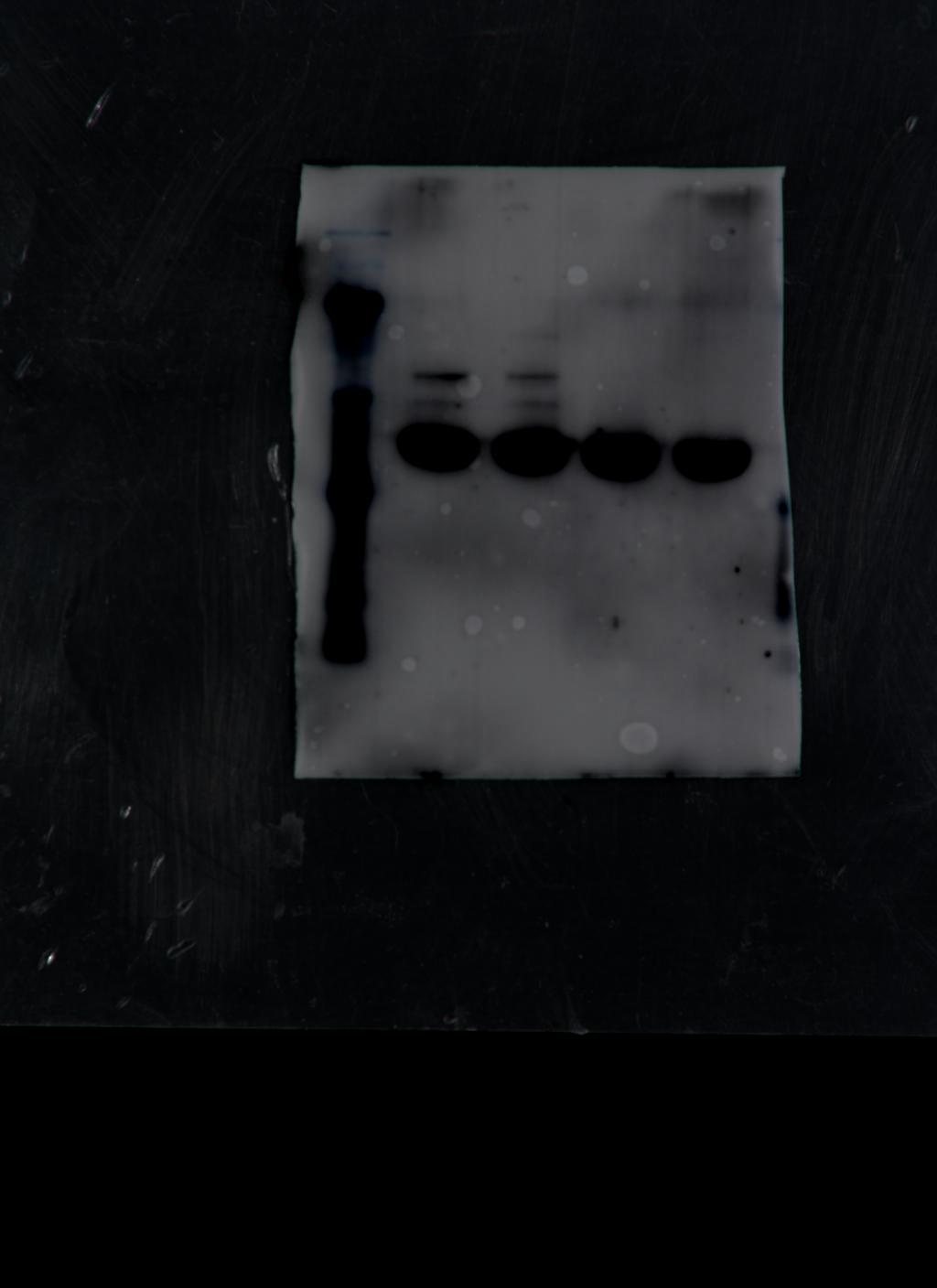

Supplement: Supplementary file 8 — Source Data for Figure 5 [file EMMM-13-e13790-s008.zip › EMM-2020-13790_SourceDataForFigure5/Fig 5C/Y_PD_GST.jpg]

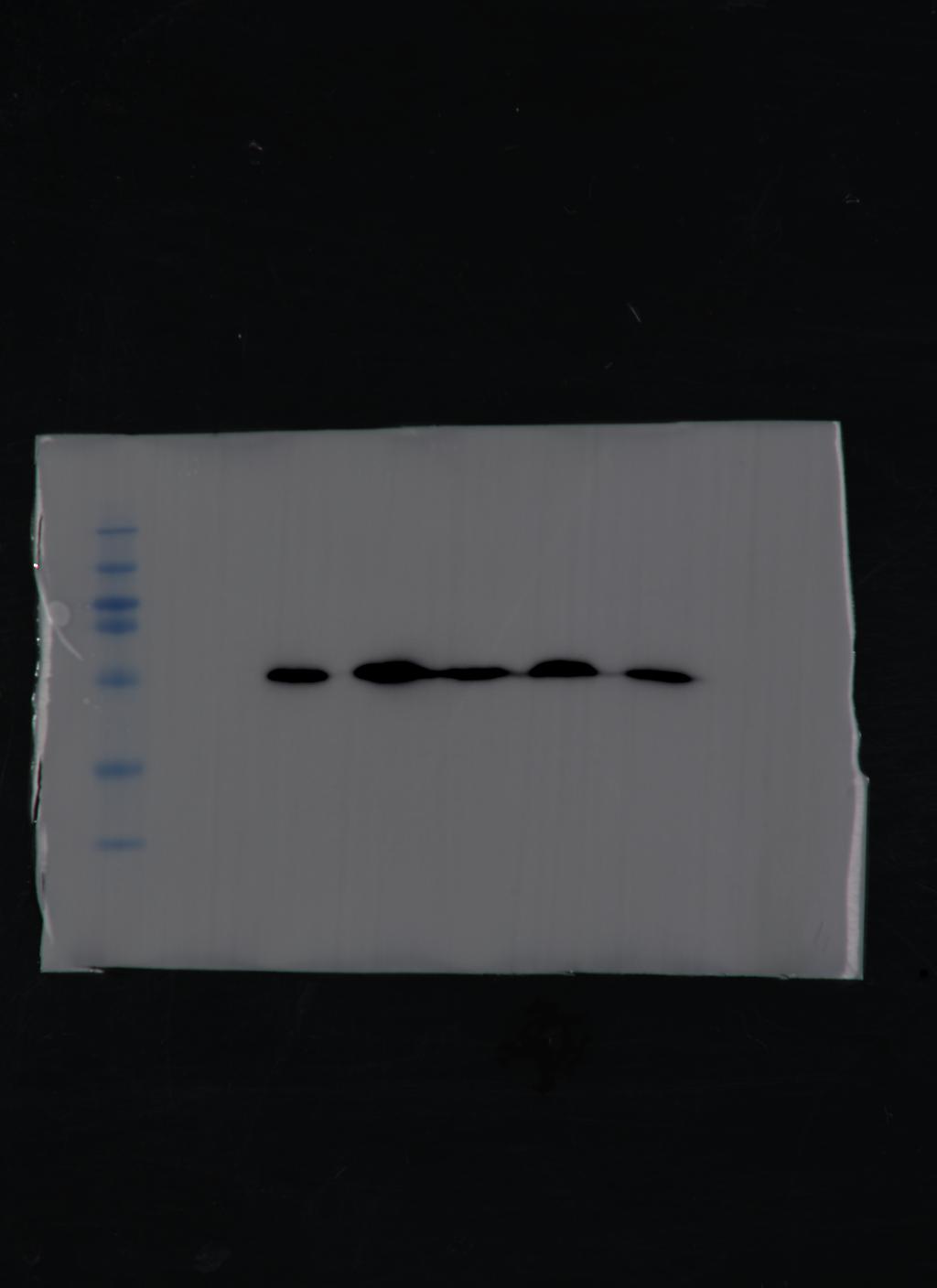

Supplement: Supplementary file 8 — Source Data for Figure 5 [file EMMM-13-e13790-s008.zip › EMM-2020-13790_SourceDataForFigure5/Fig 5D/single_Input_Flag.jpg]

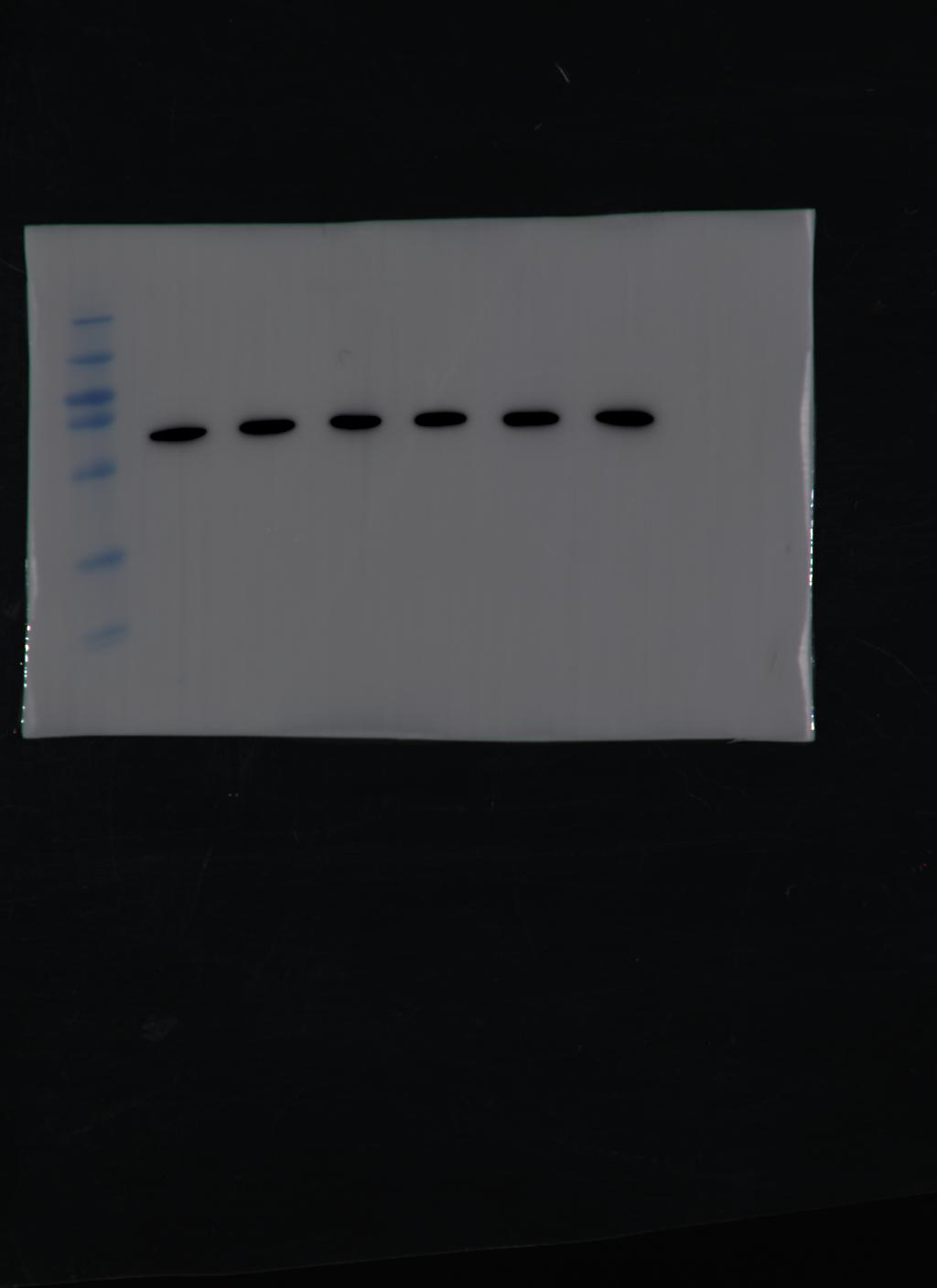

Supplement: Supplementary file 8 — Source Data for Figure 5 [file EMMM-13-e13790-s008.zip › EMM-2020-13790_SourceDataForFigure5/Fig 5D/single_Input_GAPDH.jpg]

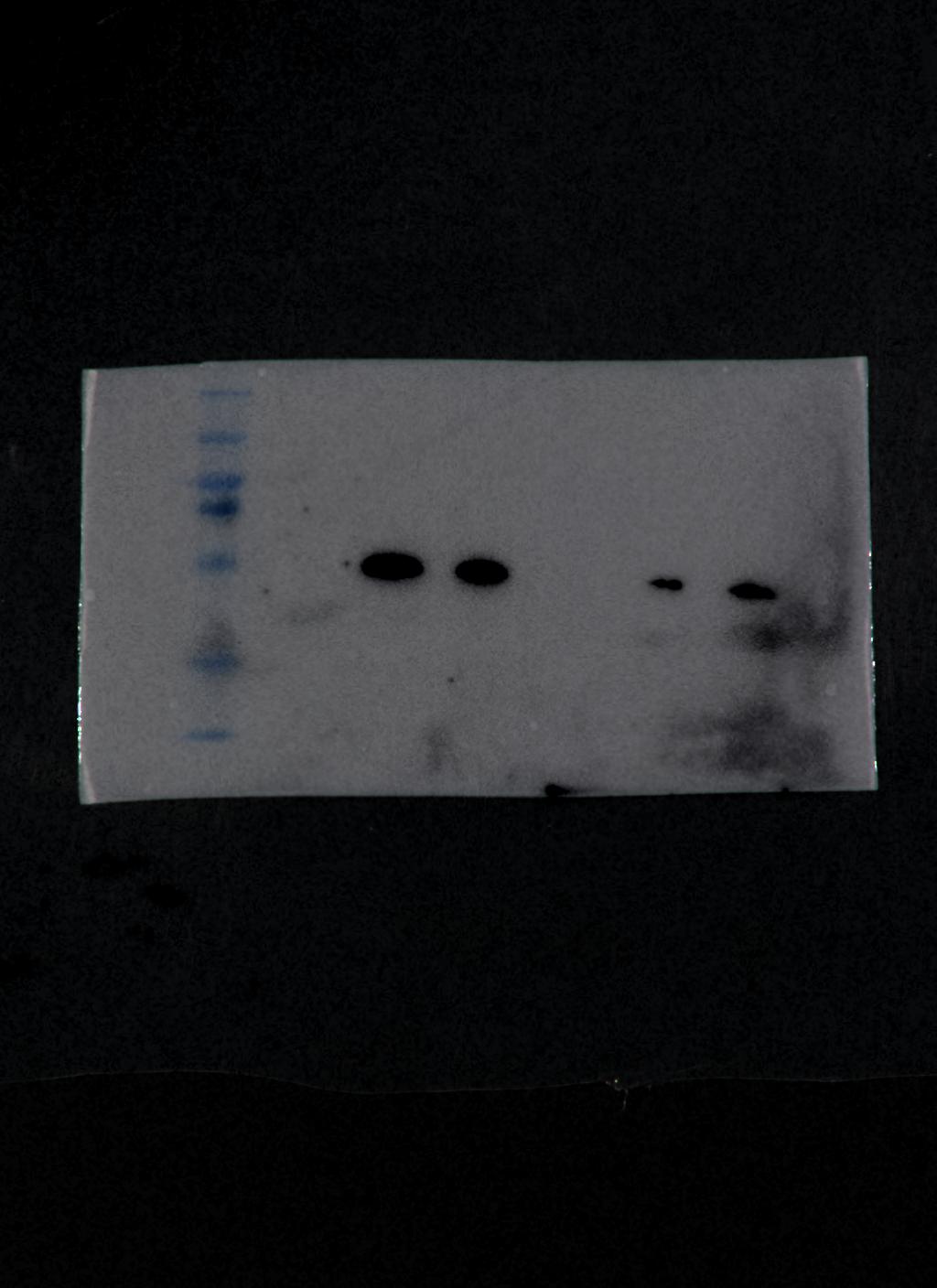

Supplement: Supplementary file 8 — Source Data for Figure 5 [file EMMM-13-e13790-s008.zip › EMM-2020-13790_SourceDataForFigure5/Fig 5D/single_PD_Flag.jpg]

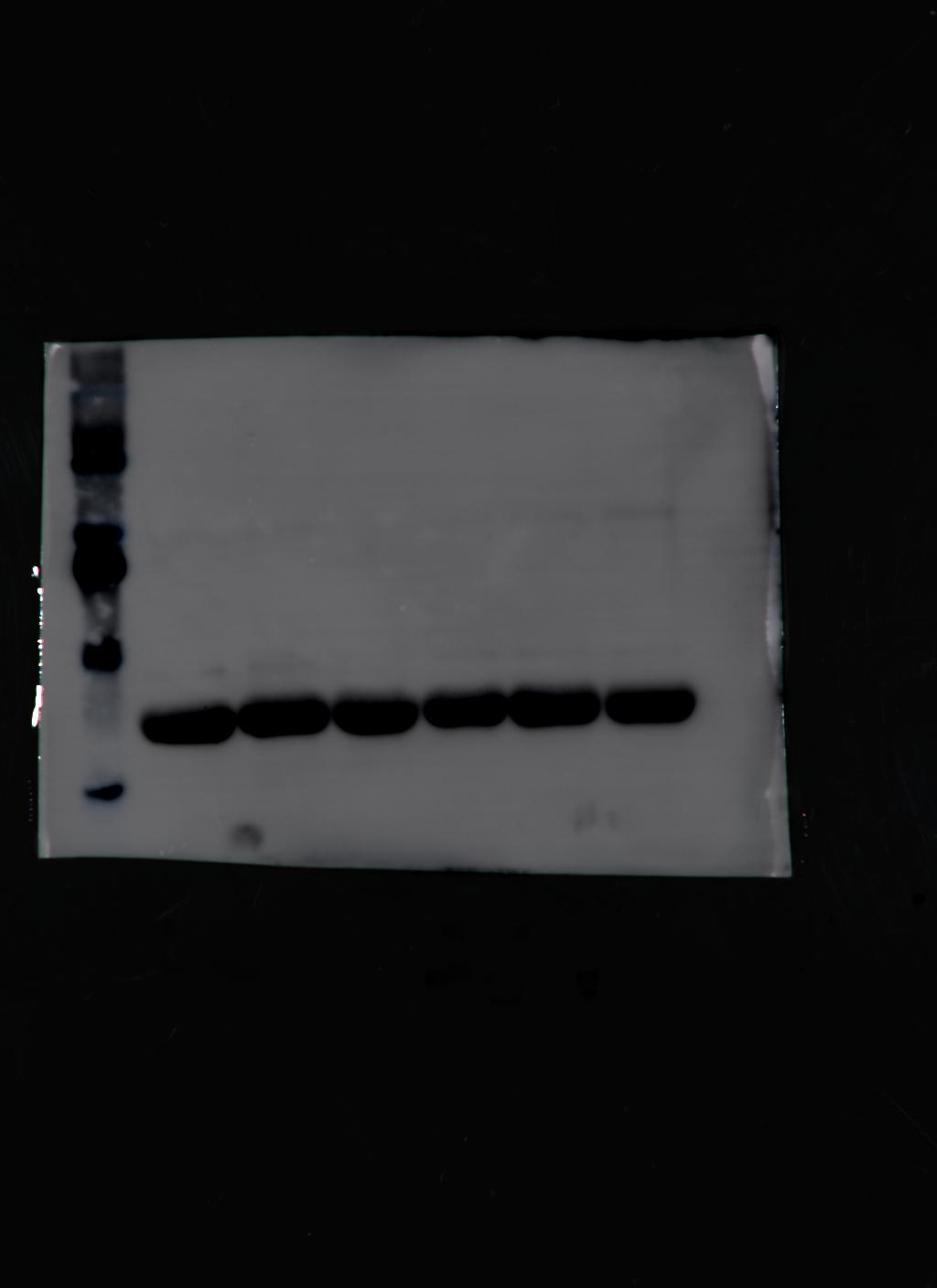

Supplement: Supplementary file 8 — Source Data for Figure 5 [file EMMM-13-e13790-s008.zip › EMM-2020-13790_SourceDataForFigure5/Fig 5D/single_PD_GST.jpg]

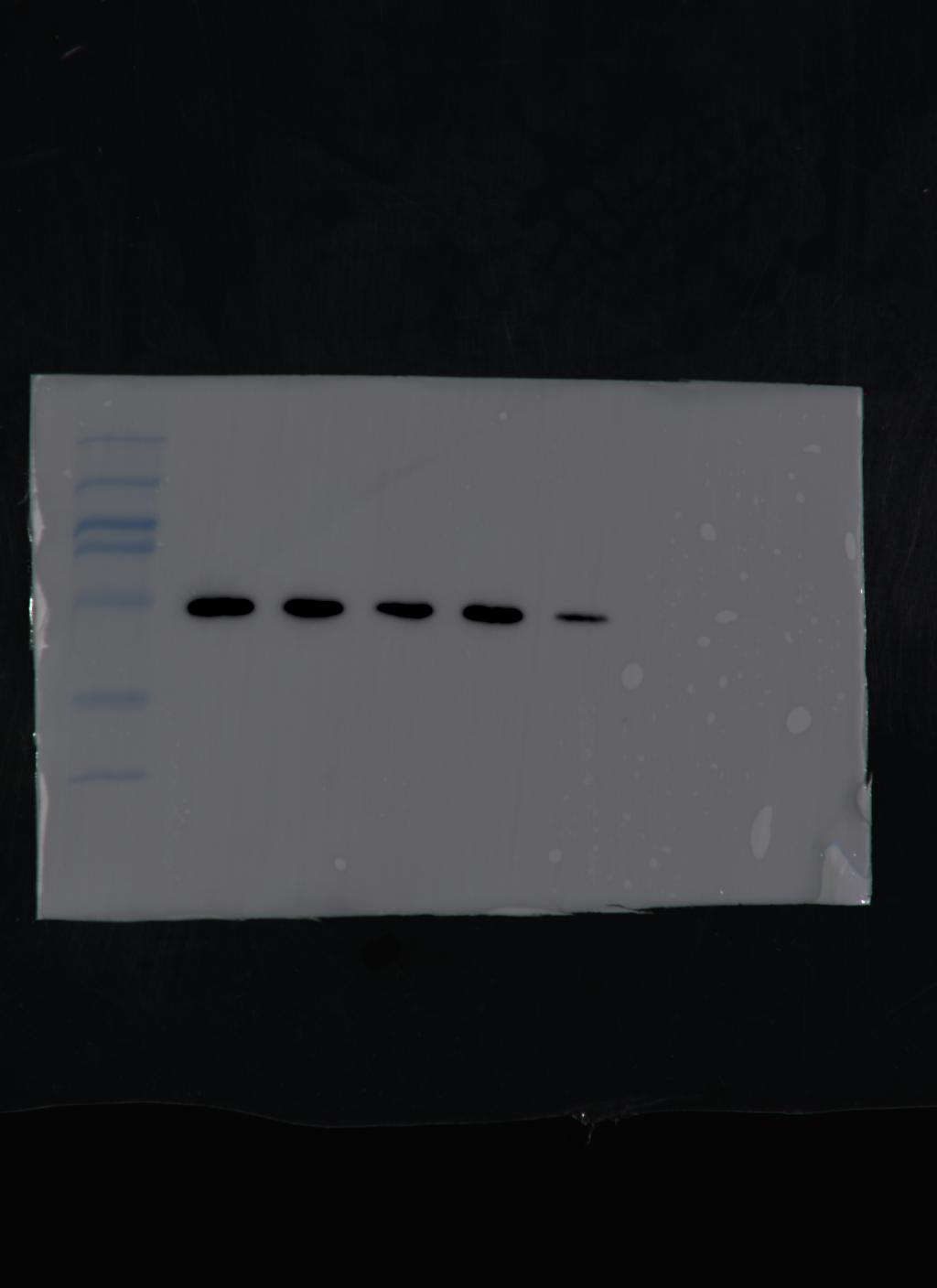

Supplement: Supplementary file 8 — Source Data for Figure 5 [file EMMM-13-e13790-s008.zip › EMM-2020-13790_SourceDataForFigure5/Fig 5E/Double_Input_Flag.jpg]

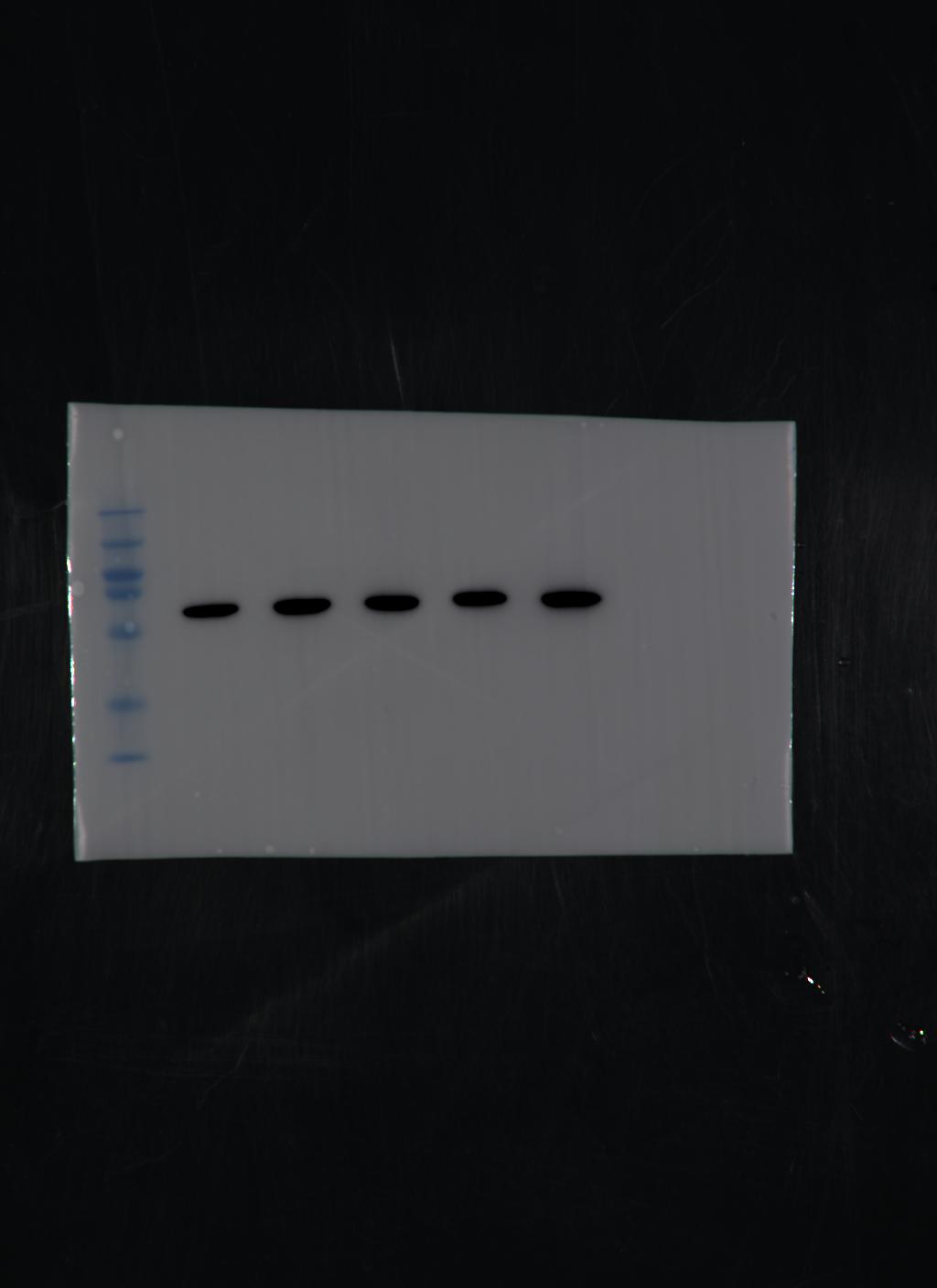

Supplement: Supplementary file 8 — Source Data for Figure 5 [file EMMM-13-e13790-s008.zip › EMM-2020-13790_SourceDataForFigure5/Fig 5E/Double_Input_GAPDH.jpg]

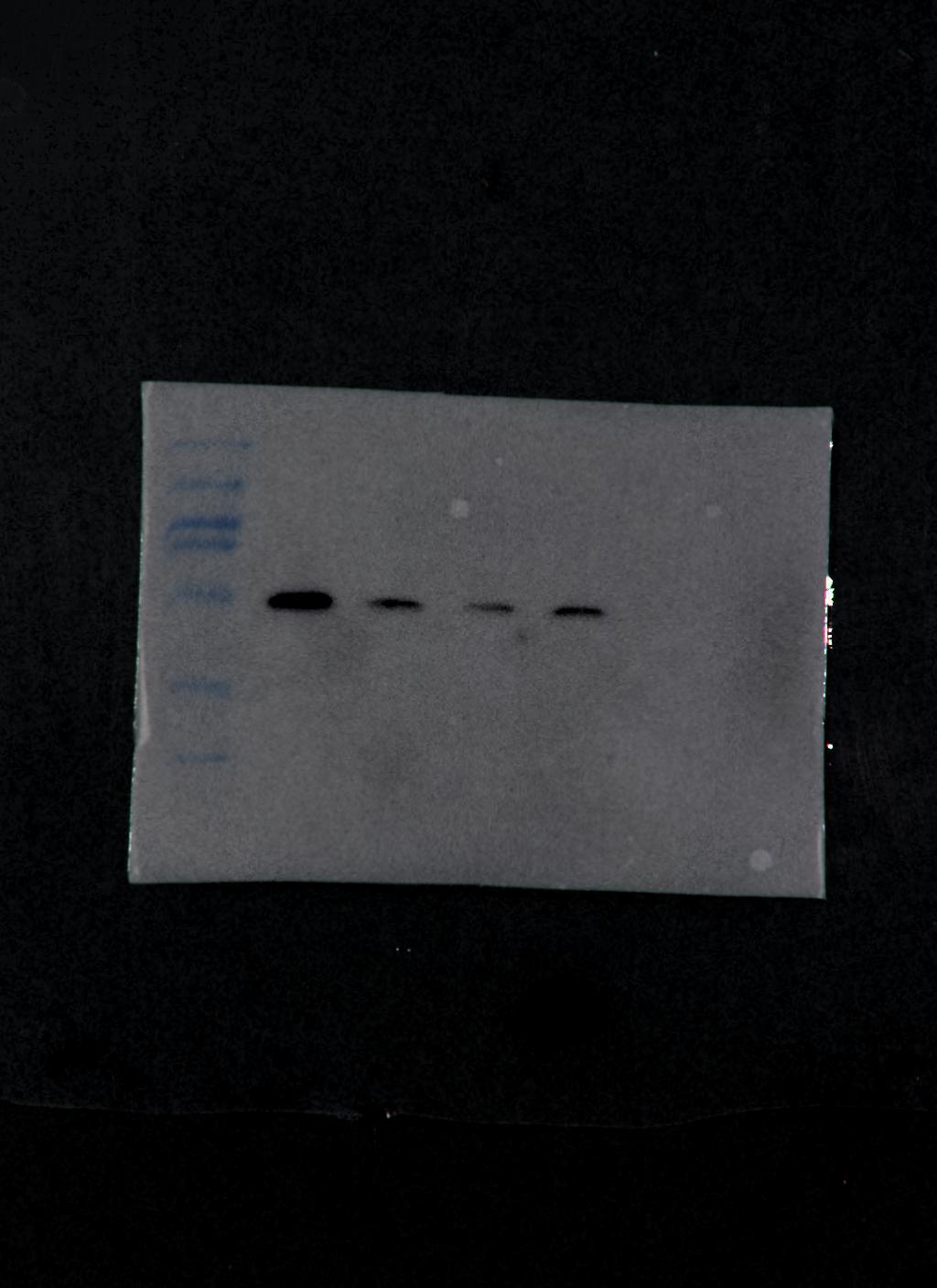

Supplement: Supplementary file 8 — Source Data for Figure 5 [file EMMM-13-e13790-s008.zip › EMM-2020-13790_SourceDataForFigure5/Fig 5E/Double_PD_Flag.jpg]

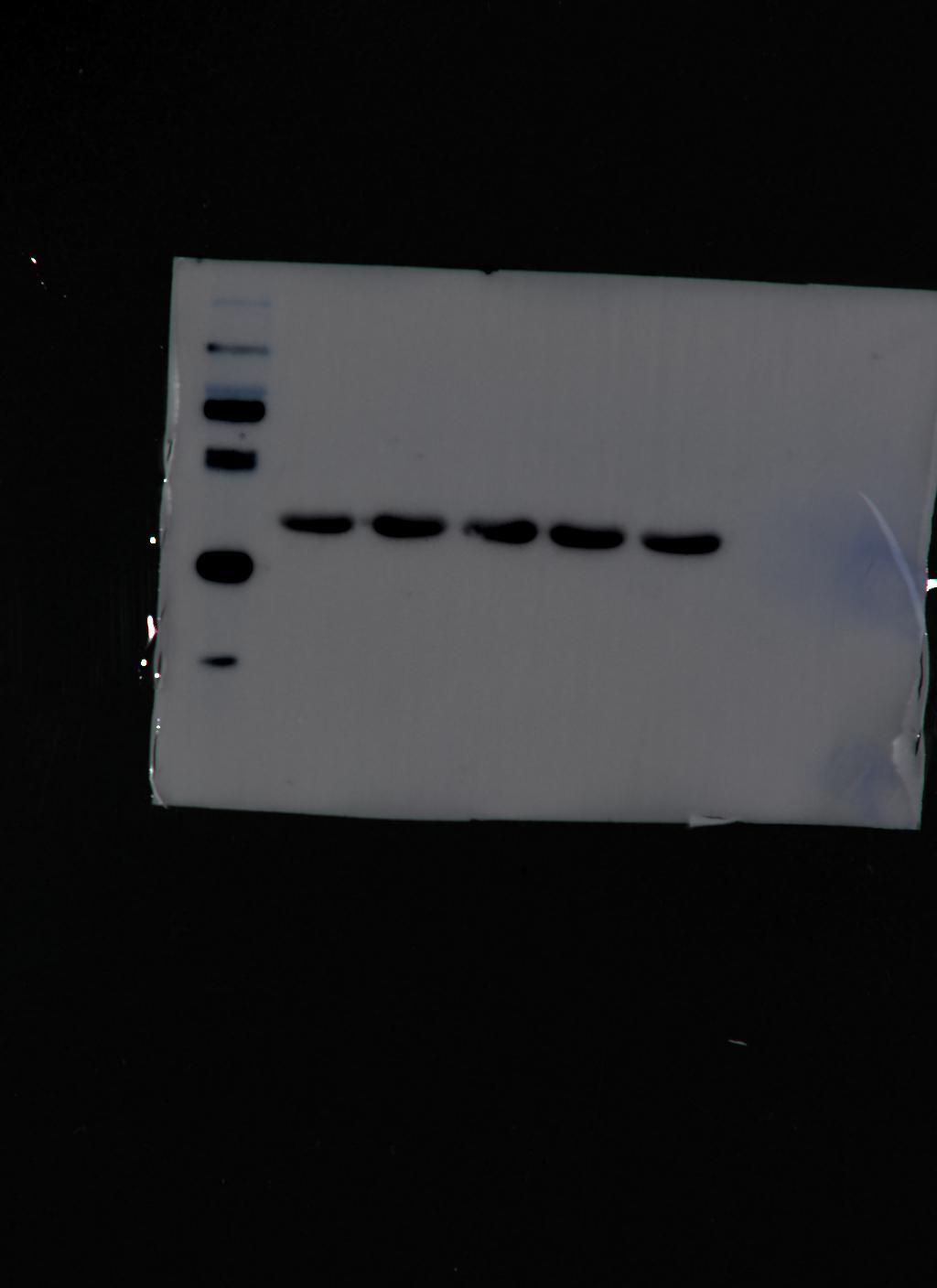

Supplement: Supplementary file 8 — Source Data for Figure 5 [file EMMM-13-e13790-s008.zip › EMM-2020-13790_SourceDataForFigure5/Fig 5E/Double_PD_GST.jpg]

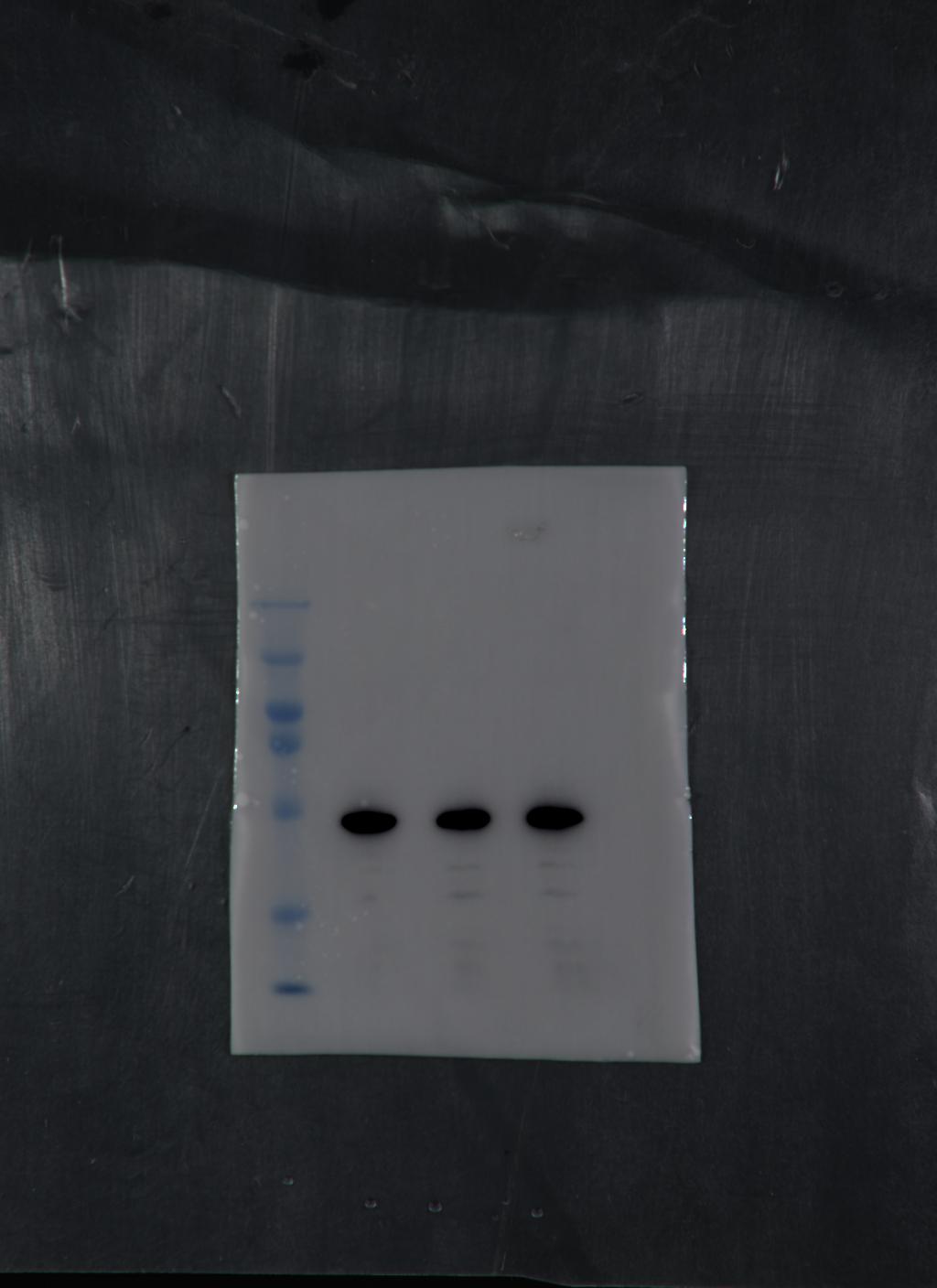

Supplement: Supplementary file 8 — Source Data for Figure 5 [file EMMM-13-e13790-s008.zip › EMM-2020-13790_SourceDataForFigure5/Fig 5F/peptides_input_Flag.jpg]

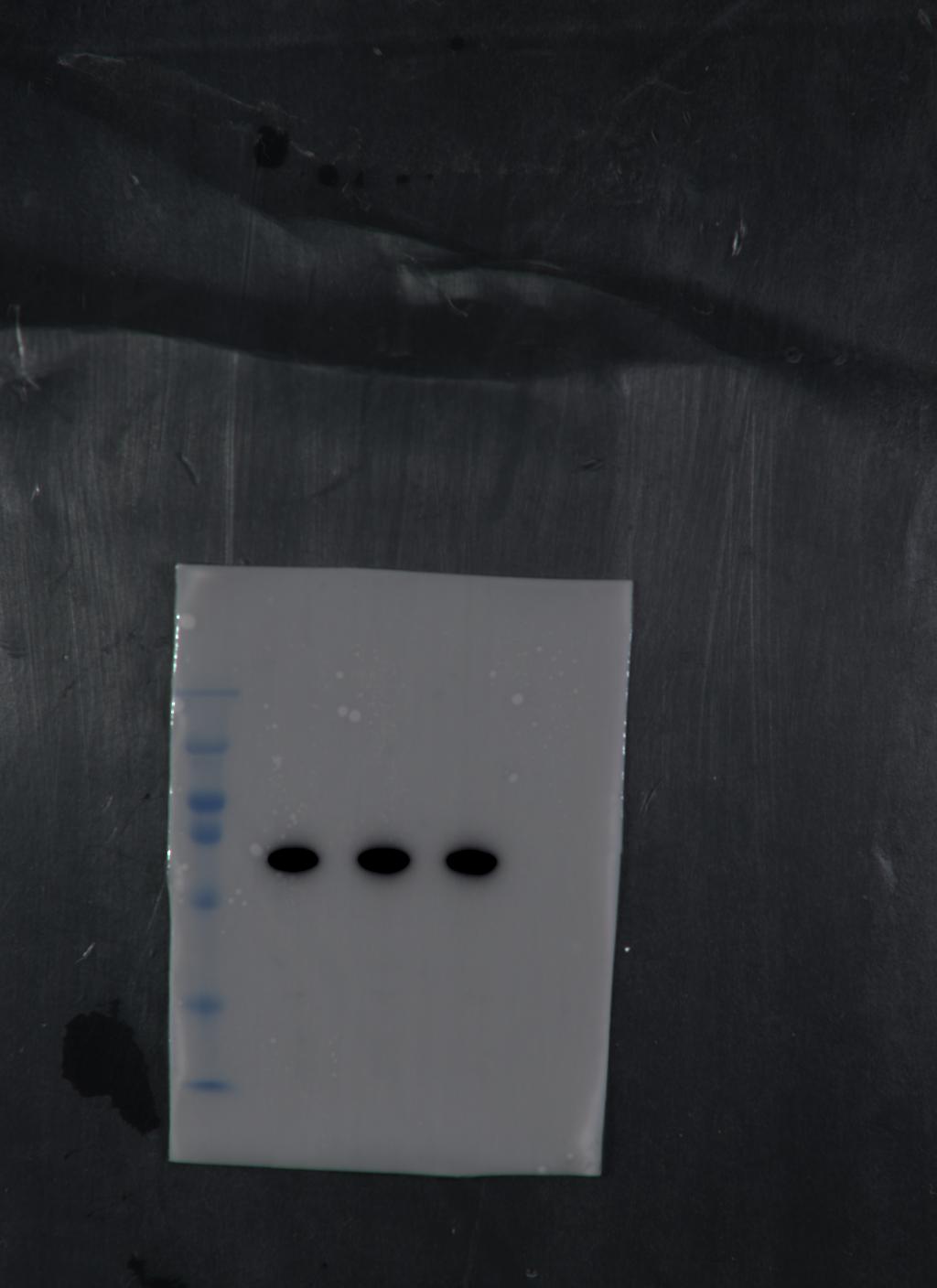

Supplement: Supplementary file 8 — Source Data for Figure 5 [file EMMM-13-e13790-s008.zip › EMM-2020-13790_SourceDataForFigure5/Fig 5F/peptides_input_GAPDH.jpg]

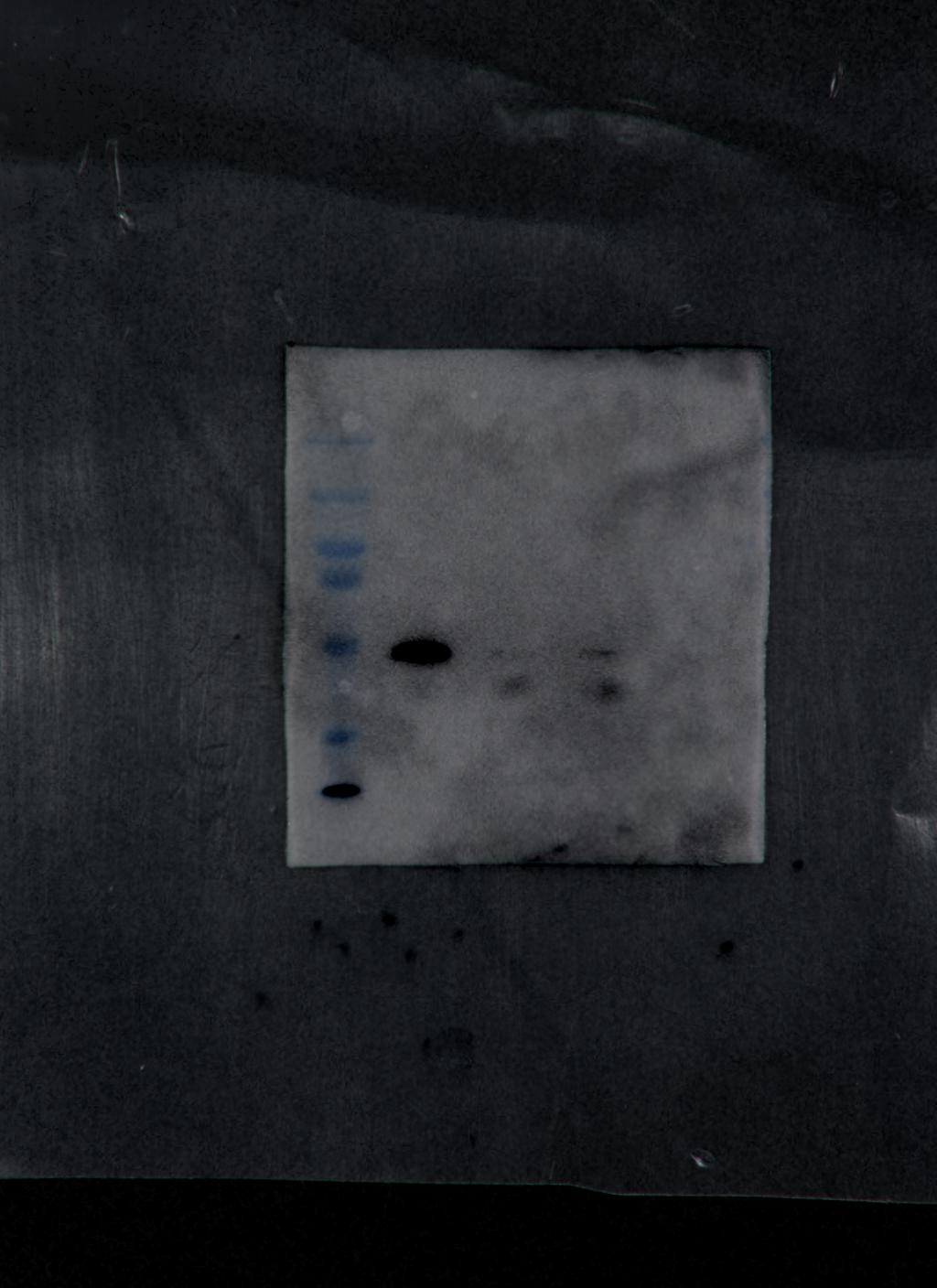

Supplement: Supplementary file 8 — Source Data for Figure 5 [file EMMM-13-e13790-s008.zip › EMM-2020-13790_SourceDataForFigure5/Fig 5F/peptides_PD_flag.jpg]

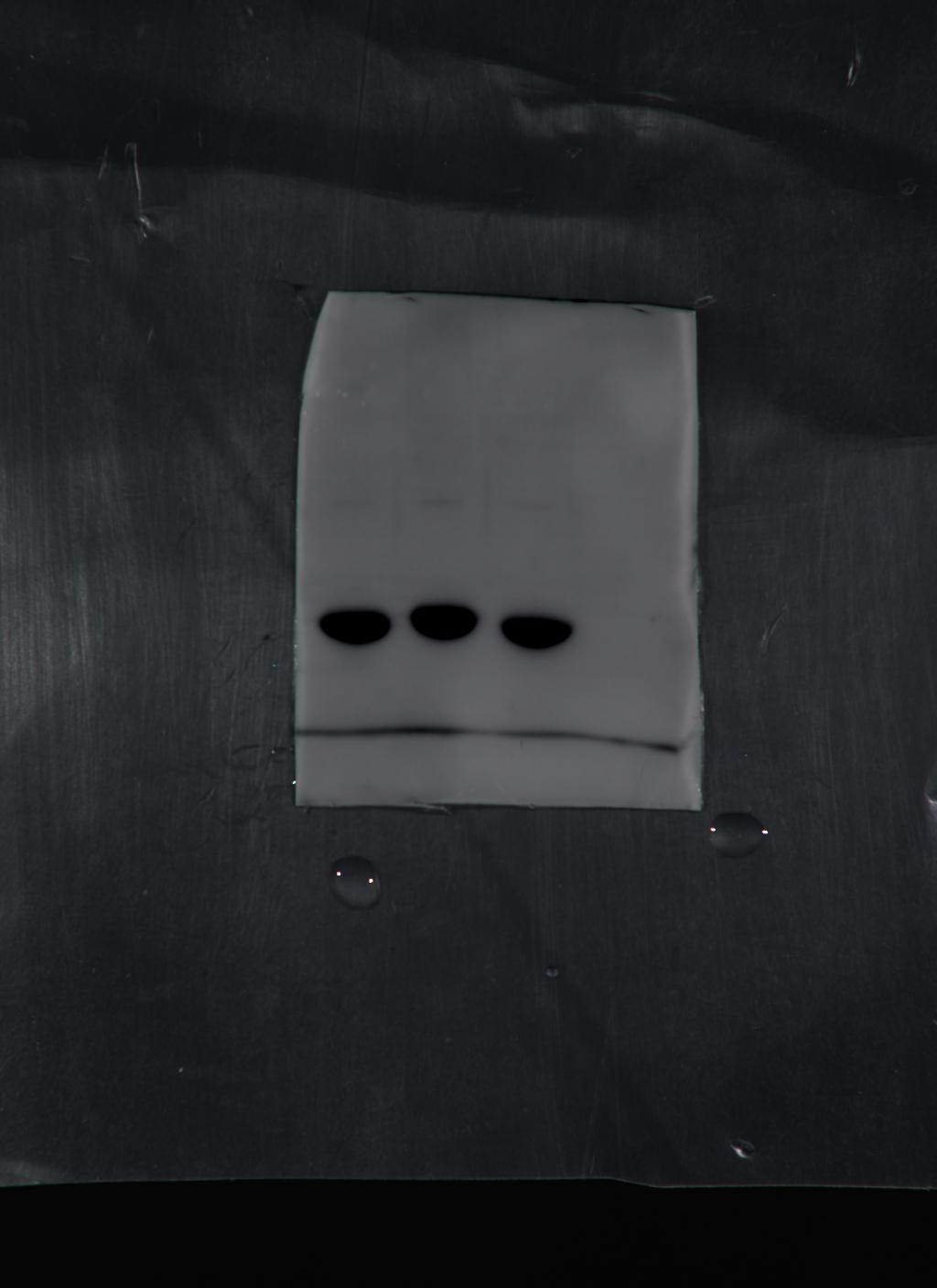

Supplement: Supplementary file 8 — Source Data for Figure 5 [file EMMM-13-e13790-s008.zip › EMM-2020-13790_SourceDataForFigure5/Fig 5F/peptides_PD_GST.jpg]
