# Supplementary material for: Discovery of a transdermally deliverable pentapeptide for activating AdipoR1 to promote hair growth
Source: EMBO Mol Med. 2021 Sep 6;13(10):e13790. doi: 10.15252/emmm.202013790 (PMC8495455; doi:10.15252/emmm.202013790)
Supplement: Supplementary file 9 [file EMMM-13-e13790-s009.pdf]

EMBO Molecular Medicine

Discovery of a transdermally deliverable pentapeptide for activating AdipoR1 to promote hair growth

Jungyoon Ohn, Kyung Wook Been, Jin Yong Kim, Eun Ju Kim, Taeyong Park, Hye Jin Yoon, Jeong Seok Ji, Miki Okada-Iwabu, Masato Iwabu, Toshimasa Yamauchi, Yeon Kyung Kim, Chaok Seok, Ohsang Kwon, Kyu Han Kim, Hyung Ho Lee, and Jin Ho Chung
DOI: 10.15252/emmm.202013790

Corresponding authors: Jin Ho Chung (jhchung@snu.ac.kr) , Hyung Ho Lee (hyungholee@snu.ac.kr)

Review Timeline:

Submission Date:	2nd Dec 20
Editorial Decision:	18th Jan 21
Revision Received:	7th Jul 21
Editorial Decision:	27th Jul 21
Revision Received:	30th Jul 21
Accepted:	20th Aug 21

Editor: Zeljko Durdevic

Transaction Report:

(Note: With the exception of the correction of typographical or spelling errors that could be a source of ambiguity, letters and reports are not edited. Depending on transfer agreements, referee reports obtained elsewhere may or may not be included in this compilation. Referee reports are anonymous unless the Referee chooses to sign their reports.)

18th Jan 2021

Dear Dr. Chung,

Thank you for the submission of your manuscript to EMBO Molecular Medicine, and please accept my apologies for the delay in getting back to you. We have received feedback from two of the three reviewers who agreed to evaluate your manuscript. Given that referee #3 will unfortunately not be able to return his/her report in a timely manner, and that both referees #1 and #2 gave similar recommendation, we prefer to make a decision now in order to avoid further delay in the process. Should referee #3 provide a report, we will send it to you, with the understanding that we will not ask for an additional revision. As you will see from the reports below, the referees acknowledge the interest of the study but also raise serious and partially overlapping concerns that should be addressed in a major revision.

Addressing the reviewers' concerns in full will be necessary for further considering the manuscript in our journal, and acceptance of the manuscript will entail a second round of review. EMBO Molecular Medicine encourages a single round of revision only and therefore, acceptance or rejection of the manuscript will depend on the completeness of your responses included in the next, final version of the manuscript. For this reason, and to save you from any frustrations in the end, I would strongly advise against returning an incomplete revision.

We realize that the current situation is exceptional on the account of the COVID-19/SARS-CoV-2 pandemic. Therefore, please let us know if you need more than six months to revise the manuscript.

I look forward to receiving your revised manuscript.

Yours sincerely,

Zeljko Durdevic
Editor
EMBO Molecular Medicine

***** Reviewer's comments *****

Referee #1 (Comments on Novelty/Model System for Author):

The authors use 2 models. I have no problems with the mouse model. but the data generated using the human ex vivo model are flawed for the reasons outlined below. I can speak with authority on this model as I am credited with having developed the ex vivo system of human hair follicle culture

The images of the hair follicles in panel (a) clearly show human hair follicles that have entered catagen. This includes both the control follicles and treated. This clearly shown by the receding pigment and also at the base of the follicle the DP is rounded up. This is classical pseudo catagen seen ex vivo. This does not therefore correlate with the Ki67 images shown in panel (c). Here the DP has not rounded. These Ki67 staining images do not therefore correlate with the gross morphology of the hair follicles shown in panel (a).
The authors need to present good histology of ex vivo hair follicles in order to convince the reader that these are not in catagen. It is well established that measurement of follicle elongation ex vivo can be misleading as follicles continue to elongate even though they no longer produce a hair fibre as the old fibre is pushed out in catagen.
If the authors have images of all their ex vivo hair follicle cultures they should go back and measure specifically hair fibre production and not whole follicle elongation

Referee #1 (Remarks for Author):

My main comment on this manuscript is with regards the data shown in Figure 3. P5 stimulated hair growth ex vivo and increased cell proliferation in the HF matrix. (A)

1. The images of the hair follicles in panel (a) clearly show human hair follicles that have entered catagen. This includes both the control follicles and treated. This clearly shown by the receding pigment and also at the base of the follicle the DP is rounded up. This is classical pseudo catagen seen ex vivo. This does not therefore correlate with the Ki67 images shown in panel (c). Here the DP has not rounded. These Ki67 staining images do not therefore correlate with the gross morphology of the hair follicles shown in panel (a).
The authors need to present good histology of ex vivo hair follicles in order to convince the reader that these are not in catagen. It is well established that measurement of follicle elongation ex vivo can be misleading as follicles continue to elongate even though they no longer produce a hair fibre as the old fibre is pushed out in catagen.
If the authors have images of all their ex vivo hair follicle cultures they should go back and measure specifically hair fibre production and not whole follicle elongation

2. I would like to see ELISA for the some of the growth factors IGF1, VEGF, HGF, PDGFA, 165FGF7, andFGF10 whose mRNA is increased by the P5 peptide treatment

Minor points

The authors state 'Based on the fact that AdipoRs are 86expressed in HF cells, including dermal papilla (DP) and outer root sheath (ORS) cells'

-This statement needs reference or the data to be shown

Referee #2 (Remarks for Author):

In the study, the authors narrowed down the sequences in the globular APN to a small skin-penetrable pentapeptide (P5), which can mimic APN's function to bind with AdipoR1. Interestingly, the P5 could accelerate human HF growth ex vivo and HF regeneration in mice in vivo via topical

application. The paper is potentially attractive to a wide range of readers. However some of the key conclusions were not well supported by the experimental data.

Major concerns:
1. The authors need to provide genetic evidence that AdipoR1 mediates the P5 function in promoting hair growth. So far the authors only provided in vitro overexpression co-IP experiments to show that P5 can interact with AdipoR1, but there is no evidence to support in their human hair follicle ex vivo, or mouse hair follicle in vivo functional experiments, P5 does indeed function through AdipoR1. For instance, in AdipoR1 cKO mice, the effect of P5 should be abrogated.
2. In Fig. 2, the authors showed that P5 activated AMPK signaling pathway in DP and ORS cell lines. However, the authors did not provide any evidence to support the same mechanism exists in human HF ex vivo or in mice in vivo after P5 application. The authors could provide p-AMPK staining in those systems to support their mechanistic claim. Without such data the related conclusion about how P5 function to promote hair growth should be modified to reflect the mechanism is only deduced.
3. The error bar in Fig.4G (with low variation) is clearly inconsistent with the data in Fig. 4F (with high variation, especially in Day14). And the statistical analysis lacks sufficient information about how many mice were quantified.

Minor concerns:
4. In the Fig.2A and 2B, the authors should compare the function of P5 and APN at the same molar concentration level to present the P5 ability to mimic the intact APN's ability in aspect of AMPK activation in vitro.
5. In Fig.2C, the siRNA K.D. efficiency for AdipoR1 should be quantified by qPCR.
6. In Fig. 5F, the relative pulldown efficiency should be quantified.

Referee #1 (Comments on Novelty/Model System for Author):

The authors use 2 models. I have no problems with the mouse model. but the data generated using the human ex vivo model are flawed for the reasons outlined below. I can speak with authority on this model as I am credited with having developed the ex vivo system of human hair follicle culture.

Response: Thank you for your positive review of this manuscript. We have carefully checked the details that you mentioned and revised the manuscript accordingly.

The images of the hair follicles in panel (a) clearly show human hair follicles that have entered catagen. This includes both the control follicles and treated. This clearly shown by the receding pigment and also at the base of the follicle the DP is rounded up. This is classical pseudo catagen seen ex vivo. This does not therefore correlate with the Ki67 images shown in panel (c). Here the DP has not rounded.

Response: Thank you for pointing this out. In the revised manuscript, we have totally revised Figure 3 with a new experiment data using *ex vivo* human hair follicle culture. The gross morphology of hair follicles in Figure 3A have been updated, which would be correlated with images in the Figure 3C and 3D. We appreciate your constructive comments.

These Ki67 staining images do not therefore correlate with the gross morphology of the hair follicles shown in panel (a). The authors need to present good histology of ex vivo hair follicles in order to convince the reader that these are not in catagen.

Response: We appreciate your constructive comments. In the revised manuscript and figures, the immunofluorescence Ki-67 images (Figure 3D) were recaptured to show a representative hair bulb structure of human hair follicles. Figure 3A and 3D correspond each other by showing the elongated shaft and the increased cell proliferation in the matrix of hair follicle treated by P5 or adiponectin.

It is well established that measurement of follicle elongation ex vivo can be misleading as follicles continue to elongate even though they no longer produce a hair fibre as the old fibre is pushed out in catagen. If the authors have images of all their ex vivo hair follicle cultures they should go back and measure specifically hair fibre production and not whole follicle elongation.

Response: We appreciate your informative comments. To avoid any potential bias in the length measurement from pseudo-catagen progression during the *ex vivo* hair follicle organ culture experiment, we re-performed this experiment and we measured the total length of pigmented hair shaft to calculate the net length of elongated hair shaft, for this revised manuscript. The results and new graph based on the new measurements were depicted in Figure 3A and Figure 3B. Thank you for thoughtful comments. The totally revised Figure 3 is presented as follows:

Figure 3

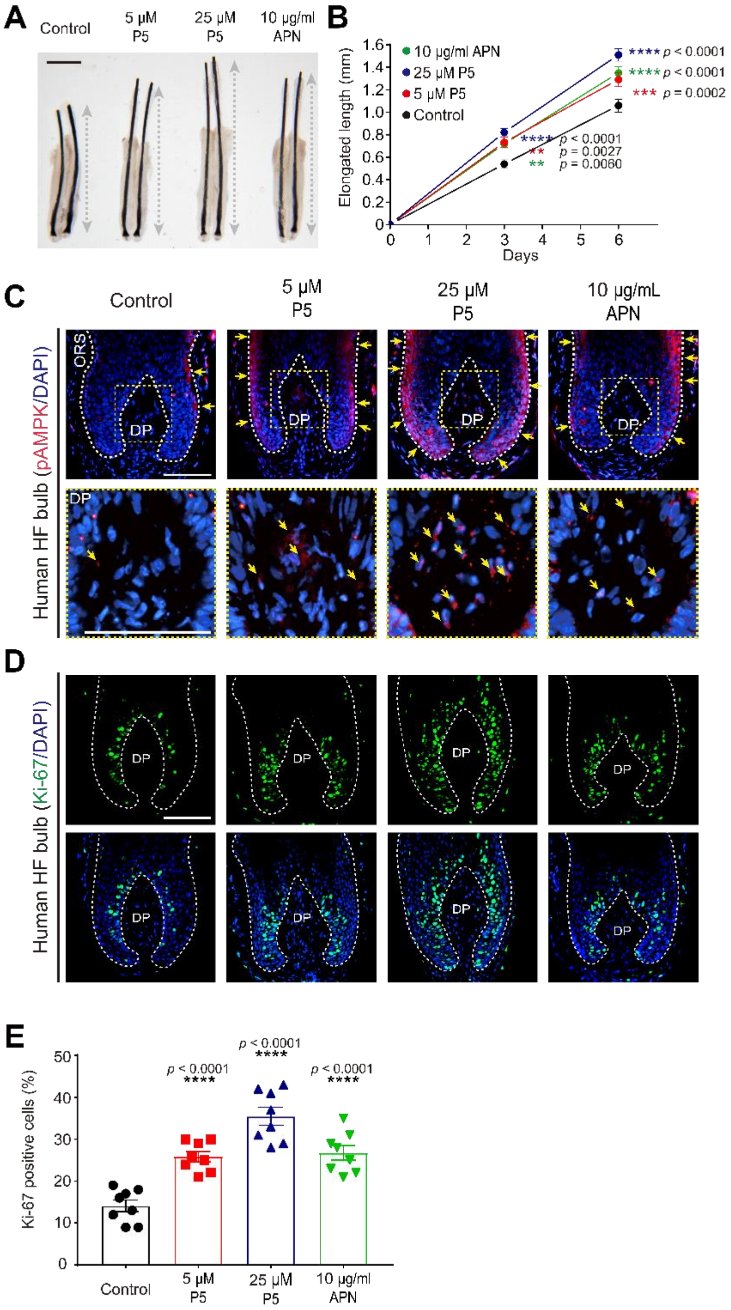

Figure 3. P5 promotes hair growth *ex vivo.*

A. *Ex vivo* cultured human HF treated with vehicle, P5, or APN for 6 days; scale bar: 1 mm; dotted gray bar: the length of pigmented hair shaft.

B. The net length of elongated hair shaft is measured and compared to the control group; Two-way ANOVA with Dunnett's test compared to the control group (38 HFs from three donors in each group).

C. IF staining (p-AMPK) of the HF bulb area. Yellow arrow indicates p-AMPK signal; scale bar: 100 μm.

D IF staining (Ki-67) of the HF bulb area; scale bar: 100 μm.

E. The numbers of Ki-67-positive cells normalized to DAPI-stained cells. One-way ANOVA with

Dunnett's test compared to the control group (*n* = 8 in each group).
Data information: In (B and E), data are presented as the mean ± SEM.

Materials and Methods

Human HF organ culture: The microdissected full-length scalp HFs in anagen VI were cut at the level of sebaceous duct and then *ex vivo* cultured for 6 days at 37°C in a 5% CO_2 atmosphere in Williams E medium (#MEPI500CA, Gibco) supplemented with hydrocortisone (10 ng/ml), insulin (10 µg/ml), L-glutamine (2 mM), and penicillin streptomycin solution (1×). To avoid any potential bias in the length measurement from pseudo-catagen progression, the total length of pigmented hair shaft of each hair follicle was measured every three days to calculate the net length of elongated hair shaft, and photographed in culture day 6 using a stereomicroscope (Olympus).

Referee #1 (Remarks for Author)

My main comment on this manuscript is with regards the data shown in Figure 3. P5 stimulated hair growth ex vivo and increased cell proliferation in the HF matrix. (A)

1. The images of the hair follicles in panel (a) clearly show human hair follicles that have entered catagen. This includes both the control follicles and treated. This clearly shown by the receding pigment and also at the base of the follicle the DP is rounded up. This is classical pseudo catagen seen ex vivo. This does not therefore correlate with the Ki67 images shown in panel (c). Here the DP has not rounded.

Response:

(This comment is duplicated to the previously addressed comments. Please refer to the response mentioned above. Thank you)

These Ki67 staining images do not therefore correlate with the gross morphology of the hair follicles shown in panel (a). The authors need to present good histology of ex vivo hair follicles in order to convince the reader that these are not in catagen.

Response:

(This comment is duplicated to the previously addressed comments. Please refer to the response mentioned above. Thank you)

It is well established that measurement of follicle elongation ex vivo can be misleading as follicles continue to elongate even though they no longer produce a hair fibre as the old fibre is pushed out in catagen. If the authors have images of all their ex vivo hair follicle cultures they should go back and measure specifically hair fibre production and not whole follicle elongation.

Response:

(This comment is duplicated to the previously addressed comments. Please refer to the response mentioned above. Thank you)

2. I would like to see ELISA for the some of the growth factors IGF1, VEGF, HGF, PDGFA, FGF7, and FGF10 whose mRNA is increased by the P5 peptide treatment.

Response: We quantified the hair growth factor proteins using the enzyme-linked immunosorbent assay (ELISA; RayBiotech, Human Cytokine Antibody Array C2000). The results were inserted in the Figure 2I-M of revised manuscript, as follows:

Figure 2

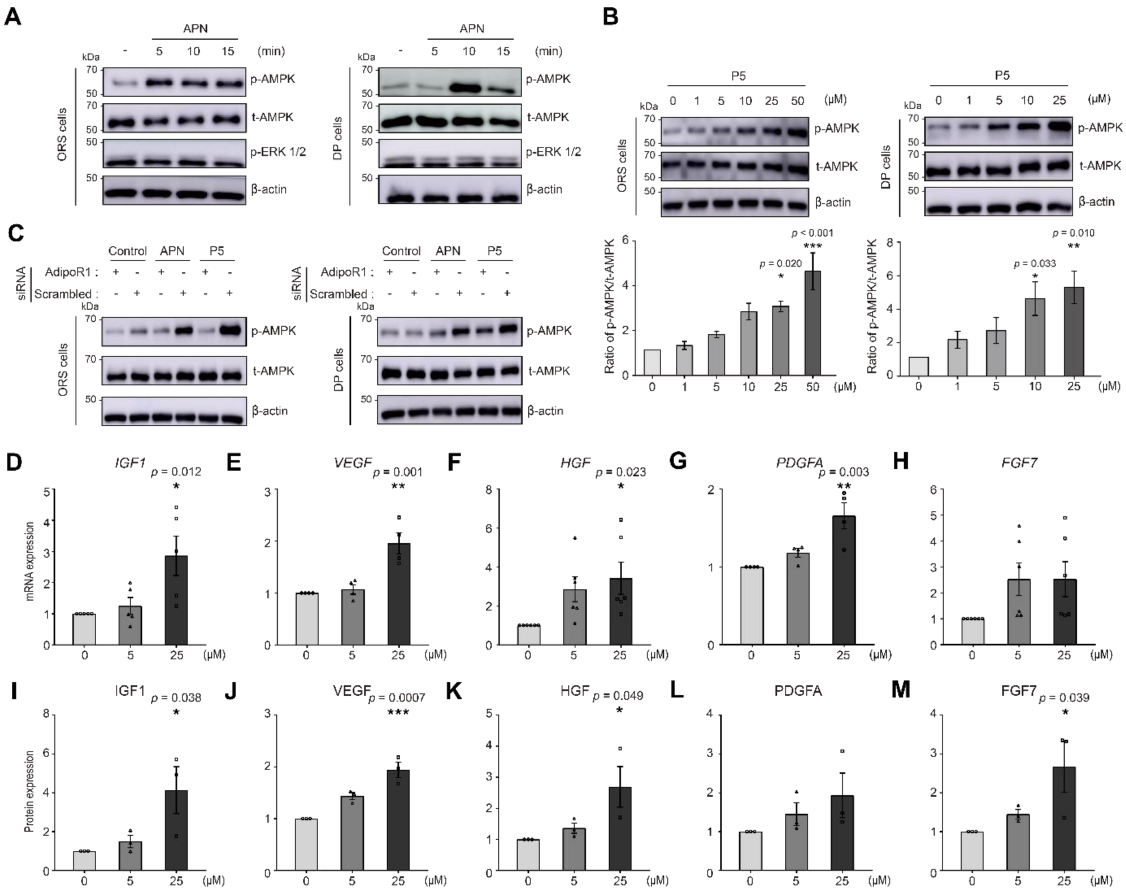

Figure 2. Both APN and P5 activate AMPK signaling pathway through AdipoR1 *in vitro*, and P5 induces hair growth factors in DP cells.

A. APN-treated ORS or DP cell lysates were analyzed for p-AMPK and p-ERK1/2.

B. P5-treated ORS or DP cells lysates were analyzed for p-AMPK and t-AMPK. Densitometric analysis for the ratio of p-AMPK protein to total AMPK protein; *n* = 5 or 4.

C. AdipoR1 siRNA-transfected ORS or DP cells were treated with APN or P5. The cell lysates were analyzed for p-AMPK.

D-H. The relative gene expression levels of growth factors in P5-treated DP cells; *n* = 4 for *VEGF* and *PDGFA*; *n* = 5 for *IGF1*; *n* = 6 for *HGF* and *FGF7*.

I-M. The relative protein levels of growth factors in P5-treated DP cells; *n* = 3.

Data information: In (B and D-M), One-way ANOVA with Tukey's test compared to each control group. data are presented as the mean ± SEM.

Materials and Methods

Growth factor protein quantitation: Human DP cells were grown on a 100-mm cell culture dish to 100% confluency. After washed by PBS three times, the cells were cultured with serum-free DMEM and treated with P5. After 48 h, the medium was collected and filtered by a 0.22-mm filter. The filtrate was then concentrated using a centrifugal concentrator (Vivaspin 6, 10-kDa molecular weight cut-off, centrifuged 2600g at 4 °C for 13 min, Sartorius Stedim Biotech). The signals of each protein in the concentrated media were detected and quantified, based on the enzyme-linked immunosorbent assay (Human Cytokine Antibody Array C2000, RayBiotech).

Your comments help us prepare our research for future readers more informatively. Thank you for thoughtful comments.

Minor points

The authors state 'Based on the fact that AdipoRs are expressed in HF cells, including dermal papilla (DP) and outer root sheath (ORS) cells'-This statement needs reference or the data to be shown

Response: We appreciate your constructive comments. It has been established in the previous study that the AdipoRs are expressed in dermal papilla cells and outer root sheath cells (*J. Invest. Dermatol.*132(12):2849-51:2849-2851 (2012)). In this line, we have inserted reference in the statement, as follow;

Based on the fact that AdipoRs are expressed in HF cells, including dermal papilla (DP) and outer root sheath (ORS) cells (Won et al, 2012),

We appreciate your informative comments.

Referee #2 (Remarks for Author):

In the study, the authors narrowed down the sequences in the globular APN to a small skin-penetrable pentapeptide (P5), which can mimic APN's function to bind with AdipoR1. Interestingly, the P5 could accelerate human HF growth ex vivo and HF regeneration in mice in vivo via topical application. The paper is potentially attractive to a wide range of readers. However, some of the key conclusions were not well supported by the experimental data.

Response: We appreciate your positive comments and interest in this manuscript. We have carefully checked your comments and clarified the questions in the revised version of the manuscript, with point-by-point.

Major concerns:

1. The authors need to provide genetic evidence that AdipoR1 mediates the P5 function in promoting hair growth. So far the authors only provided in vitro overexpression co-IP experiments to show that P5 can interact with AdipoR1, but there is no evidence to support in their human hair follicle ex vivo, or mouse hair follicle in vivo functional experiments, P5 does indeed function through AdipoR1. For instance, in AdipoR1 cKO mice, the effect of P5 should be abrogated.

Response: Thank you for constructive comments. We totally agreed that it is necessary to present the evidence that P5 works through AdipoR1 in *in vivo*. In this line, we added *in vivo* functional experiments in this revised manuscript, using a genetically AdipoR1 knock-out mouse (*Adipor1*$^{-/-}$ mouse) which was used in previous research (*Nature Medicine* 13(3):332–339 (2007) and *Nature* 28;503(7477):493–499 (2013)). It was confirmed that the anagen induction ability of P5 is abrogated in *Adipor1*$^{-/-}$ mouse *in vivo* in collaboration with Dr. Miki Okada-Iwabu, Dr. Masato Iwabu and Dr. Toshimasa Yamauchi in Tokyo University, Japan (the researchers updated in the author lists), as follows:

Appendix Figure S2

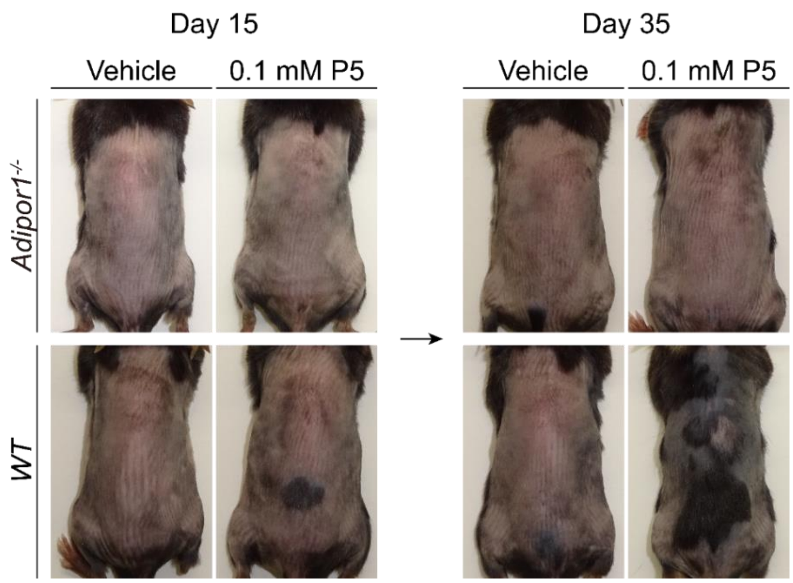

Appendix Figure S2. Topical P5 treatment on *Adipor1*$^{-/-}$ and *WT* mice

In *Adipor1*$^{-/-}$ mice, anagen hair cycle is not induced by P5 treatment.

Based on this newly added experimental data (the *in vivo* experiment data with *Adipor1*$^{-/-}$ mice), manuscript has been updated (Figure 4I to 4L). We have found that the hair cycle scores, an indicator of hair follicle anagen induction, were not different between the two groups (vehicle-treated and P5-treated *Adipor1*$^{-/-}$ mice) for 35 days (Figure 4I and 4J). The histologic evaluation for cutaneous tissue also confirmed that P5 treatment could not induce anagen cycle in hair follicle in the *Adipor1*$^{-/-}$ mice, similar to those in the vehicle-treated mice (Figure 4K). The anagen induction score was not different between two groups (Figure 4L), as follows:

Figure 4

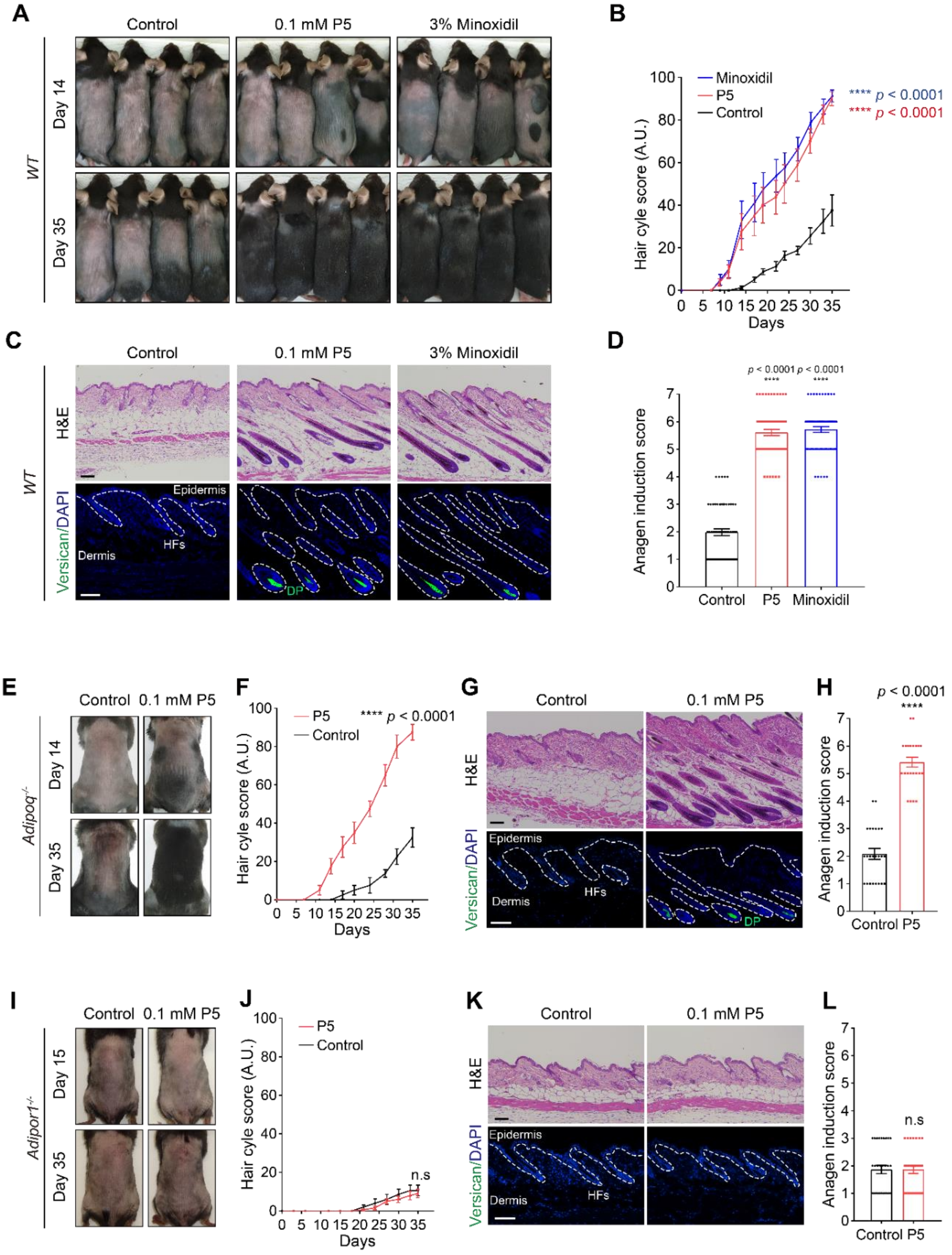

Figure 4. Topical P5 treatment induced the anagen hair cycle through AdipoR1 *in vivo*.

A. Gross morphology of vehicle-, P5-, or minoxidil- treated *WT* mice.

B. Hair cycle scores of vehicle-, P5-, or minoxidil- treated *WT* mice; n = 16 in each group; Two-way ANOVA with Dunnett's test compared to the control group.

C. H&E and IF for versican of skin tissue; scale bars: 100 µm.

D. Anagen induction scores of vehicle-, P5-, or minoxidil- treated *WT* mice; One-way ANOVA with Tukey's test compared to the control group.

E. Gross morphology of vehicle-, or P5- treated *Adipoq*$^{-/-}$ mice.

F. Hair cycle scores of vehicle-, or P5- treated *Adipoq*$^{-/-}$ mice; *n* = 4 in each group; Two-way ANOVA.

G. H&E and IF for versican of skin tissue; scale bars: 100 µm.

H. Anagen induction scores of vehicle-, or P5- treated *Adipoq*$^{-/-}$ mice; Unpaired t test.

I. Gross morphology of vehicle-, or P5- treated *Adipor1*$^{-/-}$ mice.

J. Hair cycle scores of vehicle-, or P5- treated *Adipor1*$^{-/-}$ mice; *n* = 5 in each group; Two-way ANOVA.

K. H&E and IF for versican of skin tissue; scale bars: 100 µm.

L. Anagen induction scores of vehicle-, or P5- treated *Adipor1*$^{-/-}$mice; Unpaired t test

Data information: All values are presented as the mean ± SEM.

Collectively, we believe that these additional data can convince future readers that our P5 functions through AdipoR1 in *in vivo*. Thank you for the insightful comments.

2. In Fig. 2, the authors showed that P5 activated AMPK signaling pathway in DP and ORS cell lines. However, the authors did not provide any evidence to support the same mechanism exists in human HF ex vivo or in mice in vivo after P5 application. The authors could provide p-AMPK staining in those systems to support their mechanistic claim. Without such data the related conclusion about how P5 function to promote hair growth should be modified to reflect the mechanism is only deduced.

Response: We appreciate your constructive comments. In this revised manuscript, Figure 3C and expanded View Figure 3 (Figure EV3) has been newly demonstrated to show the evidence that P5 activates AMPK signaling pathway in human hair follicle *ex vivo* and in mice *in vivo* after topical treatment.

In Figure 3C, the phosphorylation of AMPK is detected in the outer root sheath cells in the bulb of human hair follicles after P5 and adiponectin treatment (the upper panel). In addition to this, the phosphorylation of AMPK is detected in the dermal papilla cells in human hair follicles (the lower panel).

In addition, Figure EV3A provides the evidence that P5 activates AMPK signaling pathway in mice *in vivo* after topical P5 application. The immunofluorescence signal intensity of p-AMPK in hair follicle structure in telogen phase (indicated by yellow arrows) is induced by P5 treatment in the endogenous adiponectin-deficient mice (*Adipoq*$^{-/-}$). The Figure 3C and Figure EV3A are presented as below:

Figure 3C

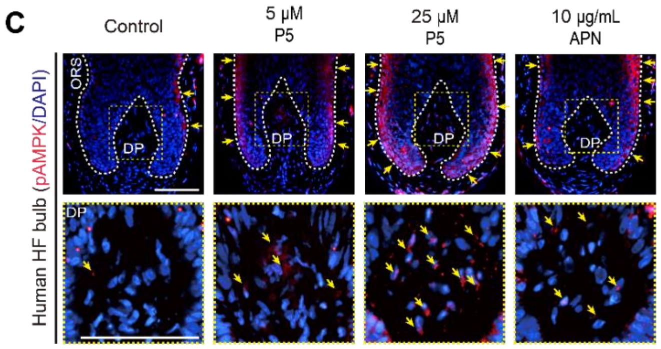

Figure EV3A

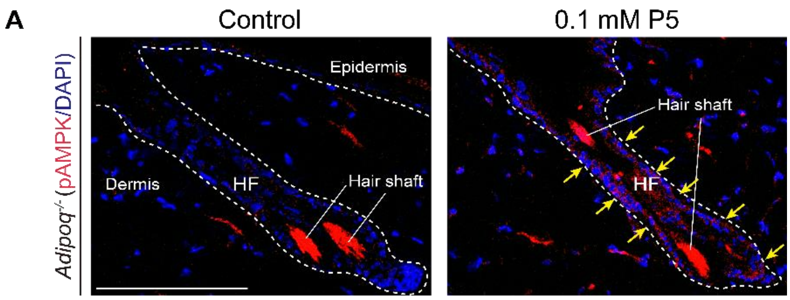

3. The error bar in Fig.4G (with low variation) is clearly inconsistent with the data in Fig. 4F (with high variation, especially in Day14). And the statistical analysis lacks sufficient information about how many mice were quantified.

Response: Thank you for constructive comment. To prevent any confusion on *in vivo* mouse studies, we revised Figure 4A and 4B to present clear images corresponding to the hair cycle score. Please note that all values are presented as the mean ± SEM. The source data is also submitted including the raw scores regarding the mouse anagen induction in this figure. Also, the total number of mice in each group was clearly denoted in the figure legend of revised manuscript, as follows:

Figure 4A and 4B

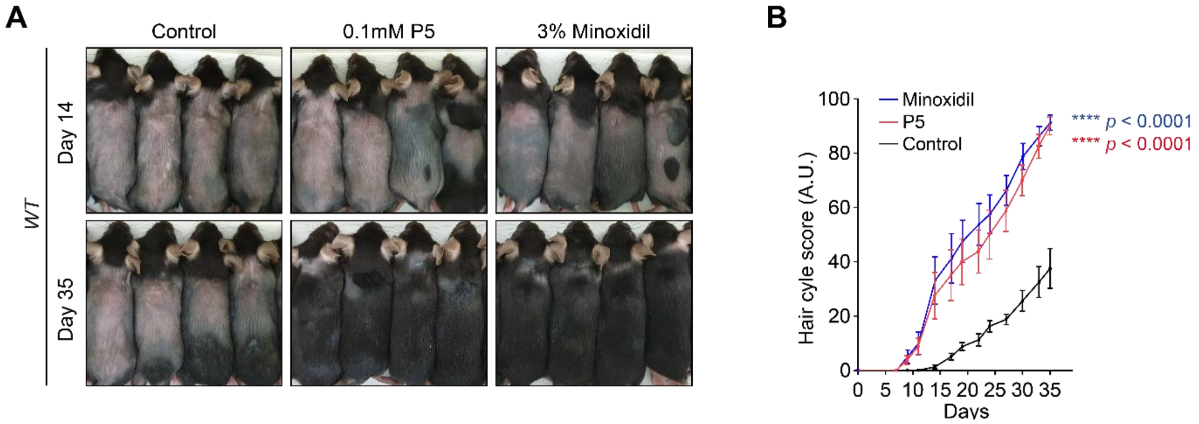

Figure 4. Topical P5 treatment induced the anagen hair cycle through AdipoR1 *in vivo*.

A. Gross morphology of vehicle-, P5-, or minoxidil- treated *WT* mice.

B. Hair cycle scores of vehicle-, P5-, or minoxidil- treated *WT* mice; n = 16 in each group; Two-way ANOVA with Dunnett's test compared to the control group.

Minor concerns:

4. In the Fig. 2A and 2B, the authors should compare the function of P5 and APN at the same molar concentration level to present the P5 ability to mimic the intact APN's ability in aspect of AMPK activation *in vitro*.

Response: As you mentioned, in Figure 2A and 2B, we examined the activation of AMPK signaling pathway using P5 (concentration: 0 - 50 μM) along with adiponectin protein (concentration: 2.5 μg/ml) as a positive control. Before we designed this experiment, we had agonized the treatment concentration due to the exactly same reason you commented here. However, it is difficult to directly quantify the efficacy based on the molar concentrations, considering the fact that adiponectin protein can exist in the form of a variety of polymers, each of which has distinct biochemical characteristic (comprehensively discussed in articles: *Biochem J*. 1;409(3):623-633 (2008) and *Endocrinology*. 149(5):2270–2282 (2008)). Monomeric adiponectin protein can interact with each other by the collagen-like domains to form stable oligomers: low-molecular-weight (trimeric adiponectin proteins) and middle molecular weight (hexameric adiponectin proteins). Furthermore, 12- or 18-mer high molecular weight isoforms can be generated during post-translational modifications (*Biochem J.* 1;409(3):623-633 (2008)). These different isoforms have distinct biochemical characteristics (*Endocrinology.* 149(5):2270–2282 (2008)). The different adiponectin protein oligomers act on different target organs and exert diverse biological functions.

Indeed, several studies in the literature evaluate the biological effects of adiponectin protein based on units of mass concentration, rather than molar concentration. The research has been conducted on mitochondria function, insulin resistance, and inflammatory diseases, with adiponectin protein in a unit of mass concentration (10 μg ml^{-1}, *Nature*. 29;464(7293):1313-1319 (2010); 0 - 50 μg ml^{-1}, *Nat Commun*. 15;6:7687 (2015); and 0 - 100 μg ml^{-1}, *Nat Med*. 7(8):941-946 (2001), respectively). A study for discovering a small-molecule *AdipoR* agonist also used the adiponectin protein concentration ranging from 10 to 50 μg ml^{-1} (*Nature* 28;503(7477):493–499 (2013)), whereas molar concentration was used for a small-molecule *AdipoR* agonist, which is the same strategy in this study. In this line, we investigate the activation of AMPK signaling pathway using P5 with molar concentration (0 - 50 μM), along with adiponectin protein in mass concentration (2.5 μg/ml) as a positive control. Thank you for the thoughtful comment.

5. In Fig. 2C, the siRNA K.D. efficiency for AdipoR1 should be quantified by qPCR.

Response: In this revised manuscript, we added the siRNA K.D. efficiency for *AdipoR1* in Appendix Figure S1, for ORS cells and DP cells, respectively. It was confirmed that siRNA targeting *AdipoR1* effectively regulated the gene expression levels.

Appendix Figure S1

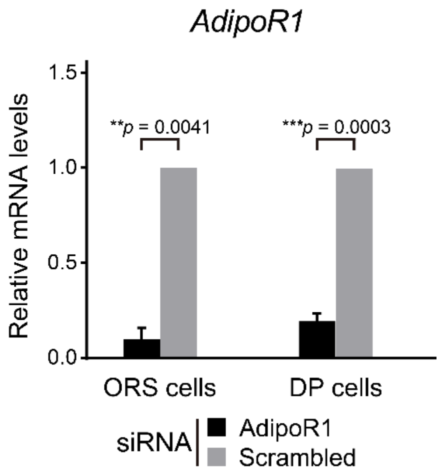

Appendix Figure S1. siRNA efficiency for *AdipoR1*

The siRNA efficiency for AdipoR1 in ORS cells (n = 3) and DP cells (n = 4)

Data information: All values are presented as the mean ± SEM. Statistical significance was determined using Welch's t test (** p < 0.01 and ***p < 0.001 compared to each scrambled siRNA treated group).

6. In Fig. 5F, the relative pulldown efficiency should be quantified.

Response: We quantified and plotted the relative pulldown efficiency of two mutant peptides in this revised manuscript. It was statistically confirmed that the two mutant peptides (GLYDF and GLYAF) showed a significantly lower pulldown efficiency for AdipoR1 compared to P5, as follows:

Figure 5F

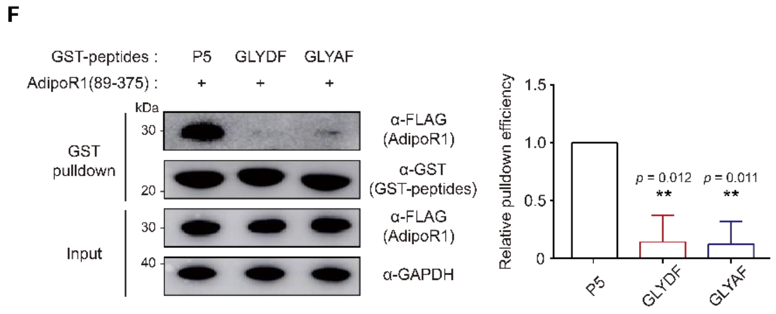

F. Pulldown of AdipoR1 (residues 89-375) with GST-P5 or GST fused to two mutant peptides (GLYDF and GLYAF). The relative pulldown efficiency is expressed as the ratio of the band intensities of bound AdipoR1 to those of AdipoR1 (residue 89-375) inputs, as indicated; n = 3.

1st Revision - Editorial Decision 27th Jul 2021

27th Jul 2021

Dear Prof. Chung,

Thank you for the submission of your revised manuscript to EMBO Molecular Medicine. I am pleased to inform you that we will be able to accept your manuscript pending the following final amendments:

1) In the main manuscript file, please do the following:
- Please check the track changes suggested by our data editors by working from the attached document.
- Make sure that all special characters display well.
- Remove text colour.
- In M&M, provide the antibody dilutions that were used for each antibody.
- In M&M, Include a statement that informed consent was obtained from all human subjects and that, in addition to the WMA Declaration of Helsinki , the experiments conformed to the principles set out in the Department of Health and Human Services Belmont Report.
- In M&M, statistical paragraph should reflect all information that you have filled in the Authors Checklist, especially regarding randomization, blinding, replication etc.
- Rename "Data and materials availability" to "Data availability" and move the sentence from p.24 "This study includes no data deposited in external repositories" to this section.
2) Synopsis:
- Please check your synopsis text and image, revise them if necessary and submit the final versions with your revised manuscript. Please be aware that in the proof stage minor corrections only are allowed (e.g., typos). Please submit synopsis text as a separate .doc file.
3) For more information: There is space at the end of each article to list relevant web links for further consultation by our readers. Could you identify some relevant ones and provide such information as well? Some examples are patient associations, relevant databases, OMIM/proteins/genes links, author's websites, etc...
4) As part of the EMBO Publications transparent editorial process initiative (see our Editorial at http://embomolmed.embopress.org/content/2/9/329), EMBO Molecular Medicine will publish online a Review Process File (RPF) to accompany accepted manuscripts. This file will be published in conjunction with your paper and will include the anonymous referee reports, your point-by-point response and all pertinent correspondence relating to the manuscript. Let us know whether you agree with the publication of the RPF and as here, if you want to remove or not any figures from it prior to publication. Please note that the Authors checklist will be published at the end of the RPF.
5) Please provide a point-by-point letter INCLUDING my comments as well as the reviewer's reports and your detailed responses (as Word file).

I look forward to reading a new revised version of your manuscript as soon as possible.

Yours sincerely,

Zeljko Durdevic

***** Reviewer's comments *****

Referee #2 (Remarks for Author):

All the questions I raised were fully addressed, I am happy with this final version of the manuscript and suggest to accept it for publication.

2nd Authors' Response to Reviewers **30th Jul 2021**

The authors performed the requested editorial changes.

2nd Revision - Editorial Decision 20th Aug 2021

We are pleased to inform you that your manuscript is accepted for publication and is now being sent to our publisher to be included in the next available issue of EMBO Molecular Medicine.

EMBO PRESS

YOU MUST COMPLETE ALL CELLS WITH A PINK BACKGROUND ↓

PLEASE NOTE THAT THIS CHECKLIST WILL BE PUBLISHED ALONGSIDE YOUR PAPER

Corresponding Author Name: Hyung Ho Lee, Jin Ho Chung
Journal Submitted to: EMBO Molecular Medicine
Manuscript Number: EMM-2020-13790

Reporting Checklist For Life Sciences Articles (Rev. June 2017)

This checklist is used to ensure good reporting standards and to improve the reproducibility of published results. These guidelines are consistent with the Principles and Guidelines for Reporting Preclinical Research issued by the NIH in 2014. Please follow the journal's authorship guidelines in preparing your manuscript.

A- Figures
1. Data
The data shown in figures should satisfy the following conditions:

- the data were obtained and processed according to the field's best practice and are presented to reflect the results of the experiments in an accurate and unbiased manner.
- figure panels include only data points, measurements or observations that can be compared to each other in a scientifically meaningful way.
- graphs include clearly labeled error bars for independent experiments and sample sizes. Unless justified, error bars should not be shown for technical replicates.
- if n< 5, the individual data points from each experiment should be plotted and any statistical test employed should be justified
- Source Data should be included to report the data underlying graphs. Please follow the guidelines set out in the author ship guidelines on Data Presentation.

2. Captions

Each figure caption should contain the following information, for each panel where they are relevant:

- a specification of the experimental system investigated (eg cell line, species name).
- the assay(s) and method(s) used to carry out the reported observations and measurements
- an explicit mention of the biological and chemical entity(ies) that are being measured.
- an explicit mention of the biological and chemical entity(ies) that are altered/varied/perturbed in a controlled manner.
- the exact sample size (n) for each experimental group/condition, given as a number, not a range;
- a description of the sample collection allowing the reader to understand whether the samples represent technical or biological replicates (including how many animals, litters, cultures, etc.).
- a statement of how many times the experiment shown was independently replicated in the laboratory.
- definitions of statistical methods and measures:
  - common tests, such as t-test (please specify whether paired vs. unpaired), simple χ2 tests, Wilcoxon and Mann-Whitney tests, can be unambiguously identified by name only, but more complex techniques should be described in the methods section;
  - are tests one-sided or two-sided?
  - are there adjustments for multiple comparisons?
  - exact statistical test results, e.g., P values = x but not P values < x;
  - definition of 'center values' as median or average;
  - definition of error bars as s.d. or s.e.m.

Any descriptions too long for the figure legend should be included in the methods section and/or with the source data.

In the pink boxes below, please ensure that the answers to the following questions are reported in the manuscript itself. Every question should be answered. If the question is not relevant to your research, please write NA (non applicable). We encourage you to include a specific subsection in the methods section for statistics, reagents, animal models and human subjects.

USEFUL LINKS FOR COMPLETING THIS FORM

http://www.antibodypedia.com
http://1degreebio.org
http://www.equator-network.org/reporting-guidelines/improving-bioscience-research-report
http://grants.nih.gov/grants/olaw/olaw.htm
http://www.mrc.ac.uk/Ourresearch/Ethicsresearchguidance/Useofanimals/index.htm
http://ClinicalTrials.gov
http://www.consort-statement.org
http://www.consort-statement.org/checklists/view/32-consort/66-title
http://www.equator-network.org/reporting-guidelines/reporting-recommendations-for-tumo
http://datadryad.org
http://figshare.com
http://www.ncbi.nlm.nih.gov/gap
http://www.ebi.ac.uk/ega
http://biomodels.net/
http://biomodels.net/miriam/
http://jjj.biochem.sun.ac.za
http://oba.od.nih.gov/biosecurity/biosecurity_documents.html
http://www.selectagents.gov/

B- Statistics and general methods

	Please fill out these boxes ↓ (Do not worry if you cannot see all your text once you press return)
1.a. How was the sample size chosen to ensure adequate power to detect a pre-specified effect size?	Sample sizes were determined according to a pilot study as well as on the basis of previous experimental experience. No statistical calculations were made to determine sample size.
1.b. For animal studies, include a statement about sample size estimate even if no statistical methods were used.	Sample sizes were determined according to a pilot study as well as on the basis of previous experimental experience. No statistical calculations were made to determine sample size.
2. Describe inclusion/exclusion criteria if samples or animals were excluded from the analysis. Were the criteria pre-established?	No data were excluded from the analysis.
3. Were any steps taken to minimize the effects of subjective bias when allocating animals/samples to treatment (e.g. randomization procedure)? If yes, please describe.	Animals/samples were allocated into experimental groups at random.
For animal studies, include a statement about randomization even if no randomization was used.	Mice were randomly allocated and shaved dorsally at 7.5 weeks of age
4.a. Were any steps taken to minimize the effects of subjective bias during group allocation or/and when assessing results (e.g. blinding of the investigator)? If yes please describe.	Animals/samples were allocated into experimental groups at random.
4.b. For animal studies, include a statement about blinding even if no blinding was done	The mouse hair growth score (a value from 0 to 100 based on the skin pigmentation and hair shaft density) was monitored and documented at designated days with the experimenters being blind to the conditions
5. For every figure, are statistical tests justified as appropriate?	Yes
Do the data meet the assumptions of the tests (e.g., normal distribution)? Describe any methods used to assess it.	No specific sumptions is used. The actual individual data from each experiment is plotted, alongside an error bar.
Is there an estimate of variation within each group of data?	No estimate of variation is used. The actual individual data from each experiment is plotted, alongside an error bar.
Is the variance similar between the groups that are being statistically compared?	Not evaluated. The actual individual data from each experiment is plotted, alongside an error bar.

C- Reagents

6. To show that antibodies were profiled for use in the system under study (assay and species), provide a citation, catalog number and/or clone number, supplementary information or reference to an antibody validation profile. e.g., Antibodypedia (see link list at top right), 1DegreeBio (see link list at top right).	Western blot analysis: Human ORS and DP cells or skin tissue were lysed (RIPA lysis buffer; #20-188; Merck Millipore). The obtained protein was then separated by 8% SDS-PAGE and transferred to a polyvinylidene fluoride membrane (Amersham). The protein transferred membranes were incubated overnight with the specific antibodies. The following antibodies were used: anti-β-actin (#MA5-15739, 1:5000; Thermo Fisher), anti-phospho-ERK1/2 (#9101, 1:1000; Cell Signaling), anti-total ERK1/2 (#9102, 1:1000; Cell Signaling), anti-phospho-AMPK (#2535, 1:1000; Cell Signaling), and anti-total AMPK (#2532, 1:1000; Cell Signaling). Then, the membranes were washed and incubated with anti-rabbit IgG or anti-mouse IgG antibodies (horseradish peroxidase-conjugated, GTX213110, GTX213111, 1:10000; GeneTex) at 25 °C for 1 h.<br>For IF staining, human ORS cells cultured in FiTC-P5, sectioned paraffin-embedded human HFs (7 μm thickness), and sectioned paraffin-embedded mouse cutaneous dorsal tissue or human skin tissue (5 μm thickness) were incubated at 4°C overnight with the following primary antibodies diluted in the diluent reagent (Invitrogen): phospho-AMPK (#2535, 1:100; Cell Signaling), Ki-67 (#M7240, 1:200; Dako), versican (#ab177480, 1:200; Abcam), or anti-AdipoR1 antibody (#ab126611, 1:200; Abcam). After three washes with PBS, the slides were incubated with the secondary Alexa Fluor 488-labeled anti-mouse or rabbit IgG antibody (#ab150077, ab150113, 1:200; Invitrogen), or Alexa Fluor 594-labeled anti-rabbit IgG antibody (#a11012) at 25°C for 1 h. Nuclei were counterstained with 4',6-diamidino-2-phenylindole (DAPI; #D1306, 1:1000; Invitrogen).
7. Identify the source of cell lines and report if they were recently authenticated (e.g., by STR profiling) and tested for mycoplasma contamination.	HEK293T cells (https://www.atcc.org/products/all/CRL-11268.aspx)

* for all hyperlinks, please see the table at the top right of the document

D- Animal Models

8. Report species, strain, gender, age of animals and genetic modification status where applicable. Please detail housing and husbandry conditions and the source of animals.	For the hair cycle modulation experiment, Adipoq-/- (stock #008195; JAX) (Ma et al, 2002), Adipor1-/- (Okada-Iwabu et al, 2013; Yamauchi et al, 2007) or C57BL/6 (KOATECH) female mice was used (Müller-Röver et al, 2001). They were housed (up to four animals per cage; 23 ± 2°C, 8:00 20:00, 12 h/12 h light/dark cycle). Mice were fed a standard chow diet and provided food and water ad libitum. Mice were randomly allocated and shaved dorsally at 7.5 weeks of age.
9. For experiments involving live vertebrates, include a statement of compliance with ethical regulations and identify the committee(s) approving the experiments.	All animal procedures were performed under the American Association for the Accreditation of Laboratory Animal Care guidelines and approved by the Institutional Animal Care and Use Committee of the Seoul National University Hospital (20-0037).
10. We recommend consulting the ARRIVE guidelines (see link list at top right) (PLoS Biol. 8(6), e1000412, 2010) to ensure that other relevant aspects of animal studies are adequately reported. See author guidelines, under 'Reporting Guidelines'. See also: NIH (see link list at top right) and MRC (see link list at top right) recommendations. Please confirm compliance.	Confirmed

E- Human Subjects

11. Identify the committee(s) approving the study protocol.	Seoul National University Hospital
12. Include a statement confirming that informed consent was obtained from all subjects and that the experiments conformed to the principles set out in the WMA Declaration of Helsinki and the Department of Health and Human Services Belmont Report.	All human research protocols conformed to the ethical principles of the WMA Declaration of Helsinki, and written informed consent was obtained from all human subjects. This experiments conformed to the principles set out in the Department of Health and Human Services Belmont Report. This study was approved by the Institutional Review Board of the Seoul National University Hospital (2003-031-1109 and 1603-114-750). All animal procedures were performed under the American Association for the Accreditation of Laboratory Animal Care guidelines and approved by the Institutional Animal Care and Use Committee of the Seoul National University Hospital (20-0037).
13. For publication of patient photos, include a statement confirming that consent to publish was obtained.	N/A
14. Report any restrictions on the availability (and/or on the use) of human data or samples.	N/A
15. Report the clinical trial registration number (at ClinicalTrials.gov or equivalent), where applicable.	N/A
16. For phase II and III randomized controlled trials, please refer to the CONSORT flow diagram (see link list at top right) and submit the CONSORT checklist (see link list at top right) with your submission. See author guidelines, under 'Reporting Guidelines'. Please confirm you have submitted this list.	N/A
17. For tumor marker prognostic studies, we recommend that you follow the REMARK reporting guidelines (see link list at top right). See author guidelines, under 'Reporting Guidelines'. Please confirm you have followed these guidelines.	N/A

F- Data Accessibility

18: Provide a "Data Availability" section at the end of the Materials & Methods, listing the accession codes for data generated in this study and deposited in a public database (e.g. RNA-Seq data: Gene Expression Omnibus GSE39462, Proteomics data: PRIDE PXD000208 etc.) Please refer to our author guidelines for 'Data Deposition'.<br><br>Data deposition in a public repository is mandatory for:<br>a. Protein, DNA and RNA sequences<br>b. Macromolecular structures<br>c. Crystallographic data for small molecules<br>d. Functional genomics data<br>e. Proteomics and molecular interactions	This study includes no data deposited in external repositories. The data that support the findings of this study are available from the corresponding author upon reasonable request.
19. Deposition is strongly recommended for any datasets that are central and integral to the study; please consider the journal's data policy. If no structured public repository exists for a given data type, we encourage the provision of datasets in the manuscript as a Supplementary Document (see author guidelines under 'Expanded View' or in unstructured repositories such as Dryad (see link list at top right) or Figshare (see link list at top right).	N/A
20. Access to human clinical and genomic datasets should be provided with as few restrictions as possible while respecting ethical obligations to the patients and relevant medical and legal issues. If practically possible and compatible with the individual consent agreement used in the study, such data should be deposited in one of the major public access-controlled repositories such as dbGAP (see link list at top right) or EGA (see link list at top right).	N/A
21. Computational models that are central and integral to a study should be shared without restrictions and provided in a machine-readable form. The relevant accession numbers or links should be provided. When possible, standardized format (SBML, CellML) should be used instead of scripts (e.g. MATLAB). Authors are strongly encouraged to follow the MIRIAM guidelines (see link list at top right) and deposit their model in a public database such as Biomodels (see link list at top right) or JWS Online (see link list at top right). If computer source code is provided with the paper, it should be deposited in a public repository or included in supplementary information.	N/A

G- Dual use research of concern

22. Could your study fall under dual use research restrictions? Please check biosecurity documents (see link list at top right) and list of select agents and toxins (APHIS/CDC) (see link list at top right). According to our biosecurity guidelines, provide a statement only if it could.	N/A
